# A systems biology approach to define mechanisms, phenotypes, and drivers in PanNETs with a personalized perspective

**DOI:** 10.1038/s41540-023-00283-8

**Published:** 2023-06-03

**Authors:** Silke D. Werle, Nensi Ikonomi, Ludwig Lausser, Annika M. T. U. Kestler, Felix M. Weidner, Julian D. Schwab, Julia Maier, Malte Buchholz, Thomas M. Gress, Angelika M. R. Kestler, Hans A. Kestler

**Affiliations:** 1 Institute of Medical Systems Biology, Ulm University, 89081 Ulm, Germany; 2 Faculty of Computer Science, Technische Hochschule Ingolstadt, 85049 Ingolstadt, Germany; 3 Institute of Pathology, University Hospital Ulm, 89081 Ulm, Germany; 4 Department of Gastroenterology, Endocrinology and Metabolism, Philipps-University Marburg, 35043 Marburg, Germany; 5 Department of Internal Medicine I, University Hospital Ulm, 89081 Ulm, Germany

**Keywords:** Regulatory networks, Dynamical systems, Logic gates

## Abstract

Pancreatic neuroendocrine tumors (PanNETs) are a rare tumor entity with largely unpredictable progression and increasing incidence in developed countries. Molecular pathways involved in PanNETs development are still not elucidated, and specific biomarkers are missing. Moreover, the heterogeneity of PanNETs makes their treatment challenging and most approved targeted therapeutic options for PanNETs lack objective responses. Here, we applied a systems biology approach integrating dynamic modeling strategies, foreign classifier tailored approaches, and patient expression profiles to predict PanNETs progression as well as resistance mechanisms to clinically approved treatments such as the mammalian target of rapamycin complex 1 (mTORC1) inhibitors. We set up a model able to represent frequently reported PanNETs drivers in patient cohorts, such as Menin-1 (MEN1), Death domain associated protein (DAXX), Tuberous Sclerosis (TSC), as well as wild-type tumors. Model-based simulations suggested drivers of cancer progression as both first and second hits after MEN1 loss. In addition, we could predict the benefit of mTORC1 inhibitors on differentially mutated cohorts and hypothesize resistance mechanisms. Our approach sheds light on a more personalized prediction and treatment of PanNET mutant phenotypes.

## Introduction

Pancreatic neuroendocrine tumors (PanNETs) are the second most common epithelial neoplasms of the pancreas, after pancreatic ductal adenocarcinoma (PDAC)^1–3^. PanNETs account for around 2% of all pancreatic neoplasms, but their incidence has doubled in the last 30 years^1,2^. In 2020, World Health Organization (WHO) classified PanNETs into two major subgroups, the well-differentiated PanNET G1 and G2, and the poorly differentiated NECs^4^. PanNETs are subclassified into grades (G) ranging from 1 to 3 based on their proliferative activity as assessed by the proliferation marker protein Ki-67 (Ki-67) labeling index and mitotic rate^1,2,5^. In 2017, the WHO revised the previous classification of pancreatic neuroendocrine neoplasms by transferring well-differentiated PanNETs with proliferation rates >20% from the undifferentiated tumor group (PNECs) to the well-differentiated group PanNET G1/G2 as a novel PanNET G3 subgroup^6^. This was necessary because a fraction of well-differentiated PanNETs with proliferation rates >20%, which would have been classified as NECs according to the WHO 2010 classification, have a tumor biology and require a treatment strategy that is closer to that of well-differentiated tumors than to that of NECs. Further classification can be made based on hormonal secretion into functional and non-functional tumors^5,7^.

The majority of PanNETs are indolent and slow-growing tumors that still preserve malignant potential. As a result, most of these tumors are moderately malignant^8^. There is a high degree of heterogeneity in tumor behavior, ranging from nearly benign to extremely aggressive^1^. Most PanNETs grow and eventually metastasize to the liver, making this progression the most common cause of death for these tumors^2,8^. Overall, the biological behavior of PanNETs is still largely unpredictable^8^. As with their progression, the pathogenesis of PanNETs is also largely unexplored^8^. Approximately 10% of PanNETs are part of familiar endocrine tumor syndromes and are therefore generated by germline mutations. The most common germline mutation is menin-1 (MEN1). Sporadic PanNETs arise from somatic mutations. Here again, 44% of cases harbor a MEN1 mutation, 43% a death-domain associated protein/ATRX Chromatin Remodeler (DAXX/ATRX) mutation, and 14% report mutations in the mechanistic target of rapamycin (mTOR) pathway (e.g., tuberous sclerosis protein (TSC))^2,5,8^.

Overall, the pathogenesis and progression of PanNET is largely unknown mechanistically. This lack of knowledge directly reflects the difficulties in the diagnosis and treatment of these tumor types^8^. For functional PanNETs (around 10% of cases) that secrete endocrine hormones, diagnosis requires extensive experience^2,8^. A large proportion of well-differentiated PanNETs are diagnosed at more advanced stages when they have metastasized^1,2,9^. Well-differentiated non-functional PanNETs, particularly G1 and low G2 (<5%), can be addressed with a “watch and wait” strategy that attempts to understand the specific tumor progression before initiating treatment strategies^8^. A more aggressive approach considers surgical resection, which is currently recommended for functionally active PanNETs, well-differentiated non-functional G1/low G2 tumors^10^. In the case of targeted therapies, mTOR inhibitors have shown prolonged progression-free survival in clinical trials and have been approved for clinical use^11^. However, the administration of these inhibitors has been associated with resistance, adding another challenge to the therapeutic management of these tumors^11–14^.

First, we studied two publicly available PanNETs datasets for their ability to be categorized using a static classification approach. Next, to unravel the behavior of PanNETs, we set up a logical model to study the dynamic behavior of the molecular pathways involved in PanNET progression. Given the largely unknown mechanisms involved in PanNETs, we chose Boolean networks as a dynamic model. Boolean models allow the reconstruction of interactions even when only qualitative knowledge is available^15^. Regulatory dependencies are constructed using logical gates, such as AND, OR, and NOT^15,16^. The final regulatory interactions for each node are summarized in Boolean functions^15,16^. In addition, as a rather simple modeling approach, they allow to model large networks and to study their dynamic behavior. The simplicity of this model derives from the fact that nodes in the network can have either active (1/ON) or inactive (0/OFF) states^15,16^. Despite this simple setup, Boolean models have been successful in recapitulating and predicting novel regulatory mechanisms in a variety of applications, such as cancer, physiology, and aging^17–21^.

By considering the most frequently mutated genes in PanNETs and the pathways involved, we were able to construct a large Boolean model through literature searches that was able to recapitulate the main phenotypic activities shown in PanNETs. By investigating the dynamics of our model, we were able to predict disease drivers and evaluate their impact in different mutational landscapes. We integrated our model predictions with patient profiles using a tailored foreign feature classifier approach. Furthermore, we set up a tumor driver identification strategy that could suggest first- and second-hit mutations after MEN1 loss capable of inducing aggressive PanNETs. Finally, we tested the application of mTORC1 inhibitors in various mutant conditions and evaluated a potential mechanism behind mTORC1 resistance emergence.

**Fig. 1:**
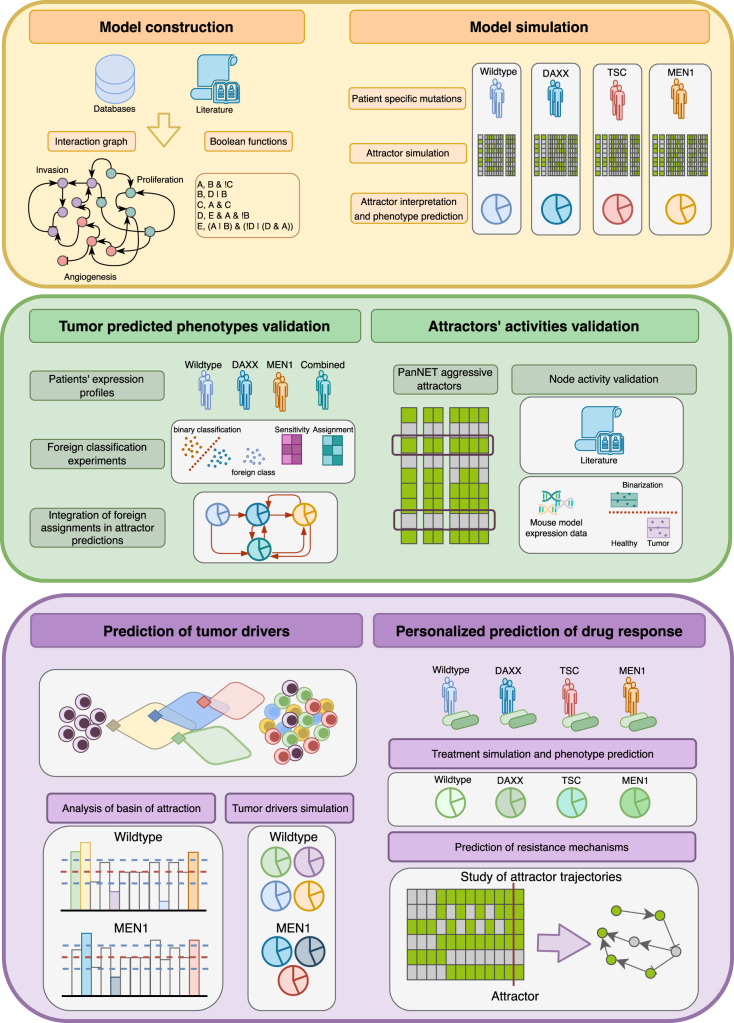
Workflow of the modeling strategy and experiments performed. Steps of the modeling approach of PanNET. Initially, a model is constructed based on literature and database search for PanNET specific regulations, which are then summarized in Boolean functions (yellow upper box, left side). Next, attractors are simulated and interpreted based on commonly mutated genes in PanNET patients (yellow upper box, right side). Predicted phenotypes are then validated via a foreign classification approach, comparing similarities of expression profiles in mutated PanNET patients´cohorts to those found in the attractor patterns (green middle box, left side). Attractors were also validated node-wise via both literature search and comparison to binarized expression profiles of the RIP-TAG2 mouse model (green mid box, right side). Additionally, the model was applied to predict tumor drivers via a study of the basins of attraction of cancer-matched attractors in both WT and MEN1 conditions (purple lower box, left side). Finally, the differential response of alternatively mutated PanNETs to a clinically approved target were studied, and a mechanism of resistance could be hypothesized via investigating attractor trajectories (purple lower box, right side).

Overall, our model comprehensively describes the heterogeneous behavior observed in the mutational landscape of PanNETs and could be applied to predict markers and resistance to gold-standard treatments with a focus on a potential personalized medicine approach. A workflow of the systems biology strategy used to model PanNETs is depicted in Fig. 1.

## Results

### Studying the classification of NETs in patient cohorts

**Fig. 2:**
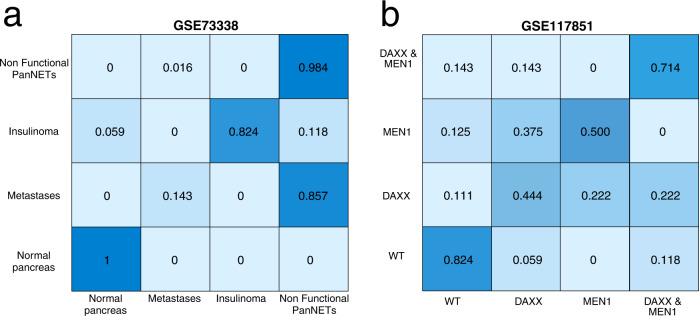
Multi class classifier experiments on NETs human expression datasets. The Figure depicts the results of a multi class classifier experiment performed on two PanNETs human expression profiles (GSE73338, GSE117851) (**a**, **b**). Results are depicted in form of a confusion matrix. Each cell of the matrix reports the probability of that group to be assigned to the corresponding one in the matrix. Color intensities reflect these probabilities, where the higher the probability the more intense the color.

A molecular mechanistic understanding of the development and progression of PanNETs is paramount for both an early diagnosis and effective treatment. In addition, experimental models to study this type of tumor entities are also scarce and controversial in terms of representativeness of the clinical condition^22^. Hence, to gain some insights into the characteristics of PanNETs, we performed first a multiclass classifier experiment on different molecular profiles of patients with pancreatic neuroendocrine tumors. The results are visualized in the form of a confusion matrix (Fig. 2). Within the confusion matrix, each cell reports the probability of that specific group to be classified as the corresponding one in the matrix. Hence, if the multiclass classifier would perfectly separate the different groups, a matrix with a diagonal of ones would be expected, meaning that each group is perfectly distinguished from the others in the system.

Here, we utilized two datasets (GSE73338 and GSE117851) reporting expression profiles of PanNETs patients grouped by functionality and metastatic status or by reported mutations, respectively. In both cases, we observed that the multiclassification experiment generally failed to reliably classify all the different groups, regardless of the consideration of driving mutations or tumor histo-pathological features. However, some groups were classified more reliably than others (e.g., insulinomas or WT tumors) and some assignments could be excluded (e.g., metastases are mostly classified as normal tissue) (Fig. 2a). Interestingly, only normal pancreas could always be correctly classified (Fig. 2a). Similarly, DAXX-mutant PanNETs show an almost evenly distributed probability of being classified in one of the other groups (Fig. 2b).

Despite the exclusion of misclassification for some classes, the multiclassification experiment could not provide a reliable classification of all tumor entities involved (Fig. 2). These results based on patient expression profiles support the high heterogeneity observed in neuroendocrine malignancies and the difficulty in predicting tumor characteristics. In this scenario, static modeling approaches, such as classification systems that only consider expression profiles, may require the integration of information from dynamic models to further understand these tumor entities. Hence, we established a Boolean model describing the main altered pathways in PanNETs and their crosstalk.

### Establishing a model to unravel the biological behavior of PanNETs

**Fig. 3:**
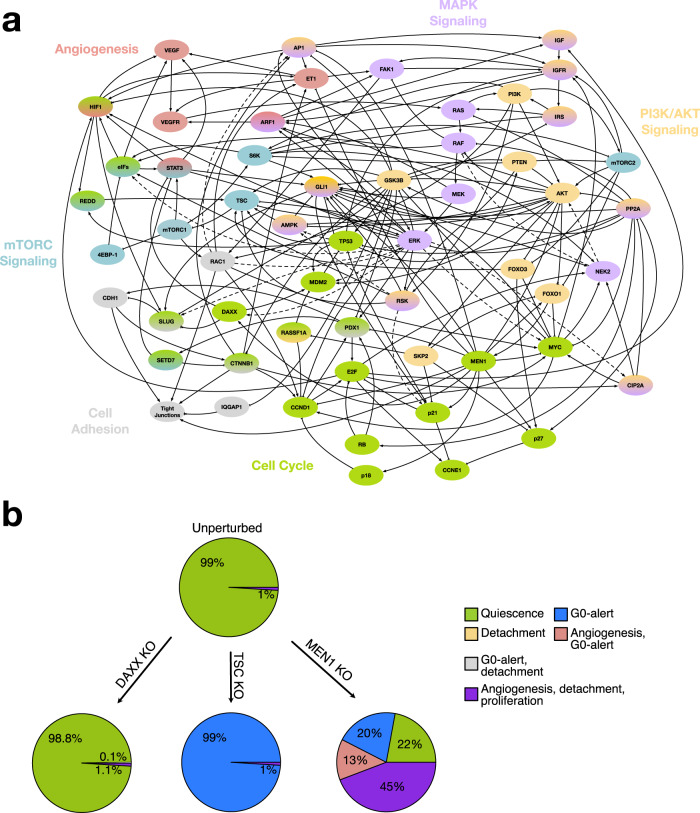
Interaction graph and dynamic behavior of the PanNETs Boolean model. **a** The 198 directed edges in the static interaction graph depict the regulatory dependencies between the 56 nodes (genes/proteins). Here, arrows show activating regulations while bar-headed lines depict inhibitory interactions. Linear cascades of interactions are represented by dashed lines between the starting and the end node. The color of each node depicts its association to a specific pathway. **b** Percentage distribution of PanNET phenotypes in the wild type (unperturbed) as well after introducing common mutations like DAXX, TSC, or MEN1 knockout (KO). The complete phenotypical landscape with the activity of each node can be found in the Supplementary Figs. 6–9.

As shown above, the simply knowing that certain genes or proteins are up- or down-regulated in a tumor entity may not be sufficient to understand and characterize tumor phenotypes. For this reason, we established a Boolean model and investigated its dynamic behavior (Fig. 3a and Supplementary Tables 1 and 2). The model is based on an extensive literature search and database screening, including nearly 200 published experimental results in its final version. We included nodes (genes/proteins) found to be mutated in PanNETs, together with their involved pathways and crosstalk. We considered regulations involving the mTOR, and phosphoinositid-3-kinase (PI3K)/protein kinase B (AKT) pathway, as well as MEN1, and DAXX/ATRX signaling. Given that PanNETs are related to angiogenesis and Insulin growth factor (IGF) signaling^2^, we also included regulatory dependencies involving VEGF, IGF, and mitogen-activated protein kinase (MAPK) signaling. Finally, to understand the mechanistic nature of PanNETs progression, we also included the regulation of cell cycle and cell adhesion pathways in our model (Fig. 3a and Supplementary Tables 1 and 2). The final model consists of 56 nodes and 198 regulatory interactions including the above-mentioned pathways and their effects on proliferation and invasion (Fig. 3a and Supplementary Tables 1 and 2). Before simulating the model, we assessed whether it reflected well-known features of biologically motivated networks. Indeed, biologically motivated models exhibit scale-free topology and resistance to noise. We were able to show that both properties are fulfilled by our constructed model, further demonstrating its potential correctness (see Supplementary Figs. 1 and 2).

In the following, we will investigate the dynamic behavior of our established model. First, we simulated the model under different mutational conditions and related the output activities to commonly reported PanNET behaviors in terms of proliferation, angiogenesis, and invasiveness. Second, we performed two validation approaches. We deepened our analysis of tumor mutations by integrating our attractor results with a foreign classification approach. In addition, we investigated node-wise the predictions of our attractor patterns related to PanNET phenotypes. Finally, we applied the model for prediction of tumor drivers and personalized evaluation of state-of the art drug targets. This workflow is depicted in Fig. 1.

### Dynamic simulation of known mutational landscapes reveals heterogenous phenotypic behavior of PanNETs

After establishing our model, we analyzed its dynamic behavior. It is known from the literature that the most frequently reported mutations for PanNETs are alterations in MEN1, DAXX, and TSC^13^. Hence, we reproduced these mutational landscapes by simulating in-silico knockouts for these genes. This was achieved by constantly setting the activity of these nodes to 1 or 0, without further consideration of their underlying mechanistic regulation. In addition to the mutations, we also simulated a wild-type (WT) tumor condition by simulating the model without any external adaptation (Fig. 3b).

To evaluate and compare the obtained attractor patterns with the experimental phenotypic data available for these mutations, we selected the activity of certain nodes in the attractors to assign a particular phenotype. To do this, we focused on phenotypes that are also described in the experimental results, such as proliferation, quiescence, G0-alert, detachment, and angiogenesis (for details on the attractor interpretation, see Supplementary Fig. 3).

Our simulations indicate that unperturbed (=WT) and DAXX knockouts have the highest percentage of quiescent cells (Fig. 3b and Supplementary Figs. 4 and 5 for complete attractor patterns). For the DAXX knockout specific simulation, we could also detect a small percentage (0.1%), showing more aggressive trades such as G0-alert and detachment (Fig. 3b and Supplementary Fig. 5). These results are in line with the known indolent behavior of PanNETs, which are known to be slow-growing tumors^8^. Interestingly, we are still able to retrieve a certain percentage of proliferative and invasive phenotypes, which may account for the malignant potential observed in these tumors.

The in-silico TSC knockout simulation resulted in 1% of cases showing a phenotype of angiogenesis, detachment, and invasion. The remaining 99% of cases show a G0-alert associated phenotype, connected to the activation of mTORC1 in the attractor pattern (Fig. 3b and Supplementary Fig. 6). Activation of the mTOR pathway is a common event after TSC loss in patients^23^. Additionally, the presence of a genetic loss of TSC does not correlate with aggressive PanNETs in most cases^23^, in accordance with our in-silico results.

The MEN1 in-silico knockout simulation showed the most severe combination of phenotypes (Fig. 3b and Supplementary Fig. 7). 44% of the cases had attractors showing angiogenesis, detachment, and proliferation. The remainder of the attractor landscape showed either complete quiescence (22%) or G0-alert alone or in combination with angiogenesis (20% and 13%, respectively). 40% of sporadic PanNETs display abnormally low nuclear staining of MEN1^24^, thus showing a severe phenotype matching our observed attractor patterns. In accordance, our in-silico knockout for MEN1 shows a more prominent proliferation and invasion potential compared to the other simulation conditions. However, most of the estimated cases still suggest an overall non or low-proliferating tumor. In addition, our results support the suggestion that MEN1-alterations play a crucial role in PanNETs tumorigenesis, while additional mutations are needed for the development of aggressive, highly proliferating tumors.

### Foreign classification experiments integrate the dynamic simulation results to elucidate differences in MEN1, DAXX, and combined mutations

**Fig. 4:**
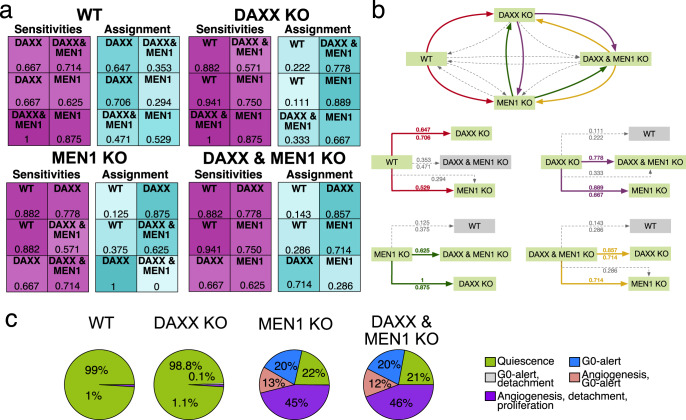
Integration of model simulation results with patients’ expression data via foreign classifier experiments. The foreign classifier experiments were performed on the GSE117851 dataset for each PanNETs patient cohort with either MEN1 or DAXX single or double mutations, and for patients without any mutations in the two genes (WT) (**a**). For each mutated group the sensitivities of the binary classifications are reported in the tables in purple on the left. On the right side, in the blue tables, instead, the assignment of the foreign class within the binary classification experiments is reported. Color intensities follow the increasing sensitivities and assignment frequencies. The results of the foreign classifier experiments are depicted in form of an interaction-like graph (**b**). Here, the direction of the arrows indicate the class to which each starting foreign class was assigned, while their colors correspond to the underlying foreign classes in the classification experiments (red for WT, green for MEN1, purple for DAXX, and orange for DAXX & MEN1). The complete graph is depicted above, summarizing all assignments taken by the foreign classification experiments. Subcircuits for each foreign class experiments are also depicted below, indicating the frequencies of assignment for each decision. In the subcircuits, if a class is never most frequently assigned, this is depicted in a gray box. Blue and thick arrows indicate assignments with higher frequencies in foreign classification experiment. Instead, while thin dashed light gray arrows indicate the less frequent assignments for each foreign classification experiment. Frequencies of assignment for all foreign classification experiment are reported in proximity of each arrow. When an assignment is equal to zero, no arrow is depicted. Attractor phenotypical landscapes are depicted based on closeness of one landscape to the other (**c**). These results were integrated to compare the molecular crosstalk arising from the dynamic model to the assignment frequencies of the classification experiments. E.g., WT is always more frequently assigned to DAXX mutated tumors and the assignment frequencies for each experiment are reported in the corresponding arrows. Finally, frequencies of assignment for all foreign classification experiment are reported in proximity of each arrow.

As shown previously, correlating PanNETs mutations with patient outcome and tumor aggressiveness can be a rather difficult task, especially considering their rarity of occurrence and the challenges of building large cohorts and models. For this reason, we have collected existing information on mouse models and patient cohorts for our described mutations (Supplementary Table 3). In addition, we developed a foreign classifier experiment to evaluate assignments between alterations in PanNETs (general strategy depicted in Supplementary Fig. 8). We used the GSE117851 dataset, which reports mRNA expression profiles of patients harboring MEN1, DAXX, or MEN1-DAXX combined mutations together with WT profiles (Fig. 4a). Our aim was not only to compare assignments retrievable from expression profiles and dynamic landscapes, but also to potentially integrate results inferred from both approaches.

Within our foreign classifier approach, binary classification experiments are performed for classes present in the dataset, always excluding one (see Supplementary Fig. 8). Afterwards, the initially excluded group is classified as a foreign class within all performed binary classification tasks and the percentage of assignment to one of the two original classes is calculated. Based on the percentage of assignment of the foreign class it is possible to deduce potential similarities (or identity) between groups. The results of this approach are presented in Fig. 4a, b.

When the WT group is considered as a foreign class, it is always preferentially assigned to DAXX mutant samples. Instead, when the decision is between doubly mutated tumor samples, the assignment is almost equally distributed with a slight preference on MEN1 mutated samples (Fig. 4b). For single mutated samples, both always avoid a preferential assignment to WT tumors. Thus, they are assigned either to the other single mutation or to the double mutated samples. Finally, a similar assignment distribution is observed for double mutant tumor samples, with a preferential assignment to the single mutant counterparts.

Next, we considered the results coming from our dynamic simulations (Fig. 4c). To fully compare the two approaches, the attractor landscape of the double mutation of DAXX and MEN1 was also computed (Supplementary Fig. 9). Consistent with the foreign classifier experiments, WT attractor simulations share more common properties with DAXX knockout simulations. In the same direction, MEN1 knockouts and double mutant simulations with MEN1 and DAXX show common attractor landscape properties. Interestingly, the DAXX attractor landscape has a high potential of quiescent properties, but it still preserves a potential of an aggressiveness through the arising of a new phenotype compared to the WT. Thus, the DAXX attractor landscape results in between of the WT and the MEN1/double mutated landscapes.

### The attractor pattern of proliferating, angiogenic, and invading PanNETs matches experimental results for tumor progression

**Fig. 5:**
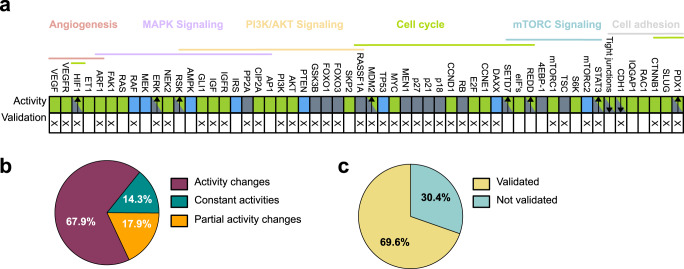
Model validation. Comparison of node activities between the quiescent single state attractor and the two severe eight states attractors (Supplementary Figures 4–7) is depicted. **a** Nodes are grouped by their pathway association. The colored rectangles show the nodes’ activities changes in the severe attractors with proliferative, angiogenic, and detachment properties compared to the quiescent one. Green rectangles represent nodes that are completely upregulated from quiescent to aggressive attractors. Gray rectangles represent nodes that are down regulated from quiescent to aggressive attractors. Rectangles with both activities represent oscillatory behaviors. Here, the arrows indicated if the overall resulting activity is up (up pointing arrow) or down (down pointing arrow) in the compared attractor patterns. Blue rectangles indicate unchanged activities. A detailed analysis of this validation can be found in Supplementary Table 3. **b** Distribution of alterations in the activity levels between the quiescent and the two severe WT attractors is shown. **c** Among the 56 nodes of the network, we were able to validate the activity of 39 nodes (69.6%).

**Fig. 6:**
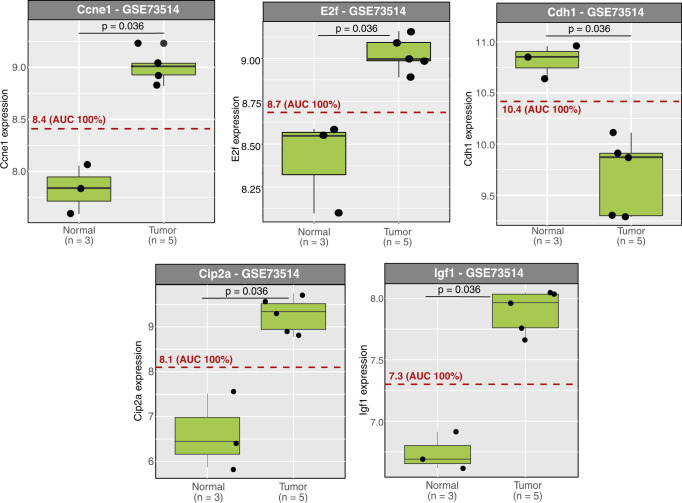
The gene expression level of normal and tumoral tissues derived from RIP-TAG2 mice in the dataset GSE73514 26. A Wilcoxon test was applied, and significant values were considered for *p* < 0.05. The dashed lines depict the binarization threshold, defining the value which separates normal and tumor tissue with simultaneously the highest sensitivity and highest specificity. The area under the curve (AUC) indicates the performance of the classifier. In all the depicted binarized expression values, an AUC of 100% was measured. Ccne1, E2f, Cip2a, and Ifg1 were significantly up-regulated in tumors, while Cdh1 was significantly down-regulated. These results matched our observed attractors patterns (Supplementary Figs. 6–9). Boxplots depict median and first and third quartiles.

After investigating the mutation-specific tumor phenotypes, we further focused on deeper studies of the specific attractor pattern associated with the angiogenesis, detachment, and proliferation-related phenotype. While the distribution of this phenotype varied between different in-silico mutational landscape simulations (WT, MEN1 loss, TSC loss, DAXX loss), the attractor pattern associated with this behavior remained highly conserved (Supplementary Figs. 4–7). Thus, we hypothesized that this attractor pattern could be further validated by matching its activities with those observed experimentally in PanNETs (Fig. 5 and Supplementary Table 4). To do so, we set up a validation procedure that compares the activities of quiescent and aggressive phenotypes in a node-wise manner (Fig. 5 and Supplementary Figs. 4–7). Given that these two phenotypes are well preserved among differentially mutated simulation conditions, we consider the comparison generally holding for all of them. Specifically, we considered the quiescent single state attractors and the aggressive eight states ones. These attractors represent the two most extreme different phenotypes that we could record in our simulations and were found in all our simulated conditions (Supplementary Figs. 4–7). For simplicity, given the less complex attractor landscape, we will from now on refer in the comparison to the quiescent and aggressive phenotype of WT (Supplementary Fig. 4). Note that this setup aims to validate activities during PanNET progression in general, thus taking away the focus from single mutations and moving it to preserved quiescent and aggressive phenotypes as the two most extreme patterns observed in our simulations. We focused both on an intensive literature search and on the analysis of a dataset from the RIP1-TAG2 mouse model^25^, which reports a clear PanNET staging in vivo (Fig. 6). Specifically, nodes mostly regulated at a transcriptional level were selected to be validated via expression data (Fig. 6), while nodes mostly regulated at the protein level (e.g., phosphorylation) were validated via published literature experimental results. First, we evaluated the changes in node activity between quiescent and cancer-related attractors, with a total of 48 nodes (85.8%) changing their activity by either up- or down-regulation (Fig. 5a, b). Next, we were able to validate the activities of key regulators in all signaling pathways and processes modeled, with a final amount of 39 validated node activities (69.6%) (Fig. 5a, c and Supplementary Table 4). We were able to correlate the activity of proteins critically involved in the progression of PanNETs, such as AKT, mTORC1, and TSC, all of which belong to the PI3K/AKT and mTORC signaling pathways (Fig. 5 and Supplementary Table 4). Additionally, Focal adhesion kinase 1 (FAK1) and Extracellular signal regulated kinase (ERK) activities as members of the MAPK signaling pathway were also matched (Fig. 5 and Supplementary Table 4). Activation of receptors and secretion of ligands, such as VEGF/VEGFR and IGF/insulin growth factor receptor (IGFR), are primary causes of aberrant activation of downstream signaling cascades^2^. These activations are also observed in our attractor landscapes, concomitant with proliferation and invasion. S-phase entry markers such as CCND1, CCNE1, and E2F in the attractor landscape were also shown to be consistent with experimentally observed activities in RIP1-TAG2 mice during the progression of PanNETs (Fig. 6). E-cadherin (CDH1) was downregulated at the tumor stage (Fig. 6), in accordance with the observed decrease in the attractor patterns and the subsequent loss of tight junctions (Supplementary Figs. 4–7).

Altogether, we could show that our attractor pattern describing an aggressive behavior in PanNETs is in agreement with published experimental data collecting tumor-related activities of the same proteins. This result also further sustains our interpretation of attractor landscapes in the phenotypic readouts.

### Model-based simulations help unravel disease drivers capable of exacerbating the PanNETs attractor landscape

**Fig. 7:**
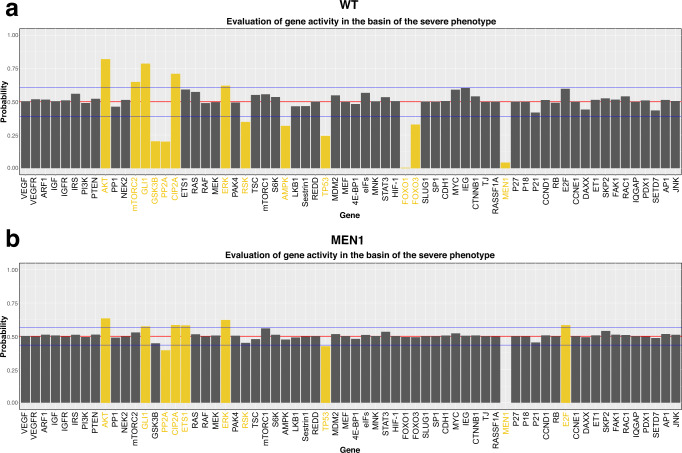
Distribution of node activities in basin states leading to the severe attractor phenotype. Probability of a certain node in the network to be active or inactive in the basin of the severe PanNETs related phenotype was computed by considering the times the node of interest was found active in 100 million examined states. The analysis was performed in both WT (**a**) and MEN1 knockout (**b**) simulations. Nodes highlighted in yellow are the ones whose activities exceeded one standard deviation (blue line) from the mean (red line) in both directions.

**Fig. 8:**
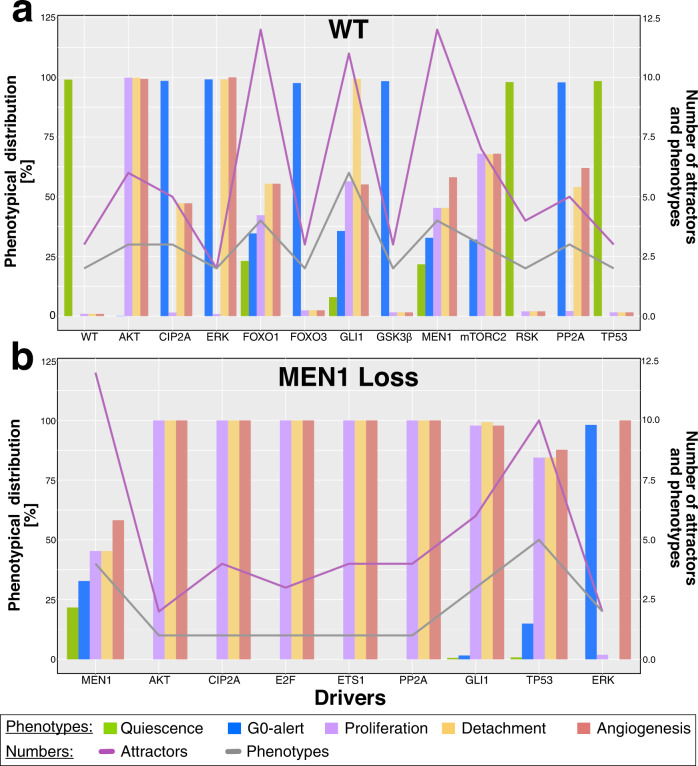
Attractor landscape of PanNETs drivers for WT and MEN1 loss tumors. The attractor related phenotypes for the PanNETs drivers identified via in-silico analyses are reported for WT (**a**) and MEN1 (**b**) simulations. Phenotypes are reported as percentages of the basin of attraction and color coded as shown in the legend. The number of attractors and total number of retrieved phenotypes is also reported below.

Predicting the progression of PanNETs is often a challenging task. While these tumors are mostly indolent, they preserve malignant potential along with a highly heterogeneous behavior^8^. In addition, while mutations such as MEN1 are known to be crucial for tumorigenesis, they are not sufficient to lead to a fully aggressive phenotype^26^, as also seen in our attractor distribution. These results eventually lead to the hypothesis that a cumulative set of mutations may be required for PanNETs to progress into aggressive, highly invasive tumors. On these grounds, we investigated whether our established model could be applied to predict tumor drivers. To this end, we investigated the network starting states leading to the aggressive tumor attractors in both WT and MEN1 conditions (starting from a seed of 100 million randomly drawn starting states) (Fig. 7 and Supplementary Fig. 10). Here, we examined activities that would exceed one standard deviation from the mean in both directions and considered these initial states as potentially relevant to the final attractor landscape. Hence, if a node has a low probability of being active in the start states leading to the severe attractor pattern, we will consider this as a hint that this node will be lost during tumor progression. Following this hypothesis, genes that met our criteria for selection as driver genes were further analyzed by performing in silico knockins and knockouts. The resulting attractor landscape was analyzed (Fig. 8).

In general, we observe a greater heterogeneity of node activities in the WT simulation compared to the MEN1 loss one (Fig. 7). This result is accompanied by a larger set of selected drivers in the WT simulation (13 in total) compared to the MEN1 loss (9 in total). Looking at the attractor landscapes, the drivers of the MEN1 loss condition lead to a severe phenotype in most cases, accompanied by a decrease in the number of attractors (Fig. 8). On the contrary, WT-related drivers generally lead to more heterogeneous and less severe phenotypes, supporting the hypothesis that additional mutations are required to induce tumor progression. In the following, the attractor landscape of each driver is presented in both WT and MEN1 loss simulations.

In the WT driver setting, no driver except for AKT in-silico knockin (constantly active AKT) was able to induce the worse phenotype related to proliferation, detachment, and angiogenesis. Activation of AKT and of the mTOR pathway is a hallmark of PanNETs^13,27–29^. Thus, this result corroborates the correctness of our simulations. In the same line, also mTORC2 was selected as a driver^30,31^. MEN1 and the Forkhead box O (FOXO)s proteins 1 and 3 were also included in the driver set. MEN1 loss is the most common mutation in PanNETs and has been connected to tumor initiation and progression^23,24,32^. Interestingly, FOXO1 is a direct and master regulator of MEN1^33^. Hence, its loss mimics the loss of MEN1 (Fig. 8a). Cellular inhibitor of PP2A (CIP2A) was shown to be downregulated in PanNETs in our expression data analysis (Fig. 6), and Protein phosphatase 2 A (PP2A) is its direct target^34^. GLI family zinc finger 1 (GLI1) was shown to be highly expressed in BON1 cells, consistent with its identification as a knockin tumor driver^35^.

Compared to the WT simulation analysis, the set of identified drivers in addition to MEN1 was smaller, but had a stronger impact on the attractor landscape (Figs. 7b and 8b). This means that the percentual distribution of simulation cases showing proliferation, detachment, and angiogenesis in the attractor increased. Although the set of additional drivers for MEN1 was smaller, most of the nodes in the driver set overlap with the WT set, with the exception of ETS protooncogene 1 (ETS1) and E2F (Figs. 7b and 8b). Nodes in common to both sets show a more severe aggressive phenotype with higher proliferative, invasive, and angiogenic properties when coupled with a MEN1 mutation (Fig. 8). A striking example of this is TP53, which had virtually no influence on the landscape of the WT simulation. However, under MEN1 loss conditions, the loss of TP53 causes a sensible worsening of the attractor landscape, suggesting that TP53 might be a good candidate as a second hit mutation. Interestingly, this hypothesis was confirmed in an experimental setup showing that TP53 alteration is associated with further tumor development in the combination with other mutations^5^. E2F and ETS1 were only found as drivers in the MEN1 loss condition. While E2F is directly connected to an increase in proliferation^36^, ETS1 is still poorly studied in PanNETs.

In summary, we have shown that our model is able to predict driver genes whose altered activities could exacerbate both WT and MEN1 loss under in-silico conditions. These drivers could be further investigated and validated in future laboratory validations, potentially uncovering markers of tumor progression.

### Model-based simulations provide efficacy of mTORC1 inhibition in different mutational landscapes and show mechanisms involved in treatment resistance

After investigating potential tumor drivers, we were also interested in identifying molecular targets for therapeutic applications. Given the relevance of the PI3K/AKT pathways in the progression of PanNETs, several studies and clinical trials have pointed out the potential relevance of the therapeutically targeting of mTORC1^11,37^. These results have led to the FDA approval of the mTORC1-specific inhibitor everolimus for the treatment of PanNETs^38^. However, mTORC1 inhibitors have often failed to yield objective responses leading to AKT reactivation^12,13,37^. Additionally, few studies have reported responses to mTORC1 inhibitors mutatant PanNET cohorts. For this reason, we investigated the mTORC1 intervention in our model to predict its efficacy in differently mutated PanNETs, resembling the potentially differential response in different patient cohorts.

**Fig. 9:**
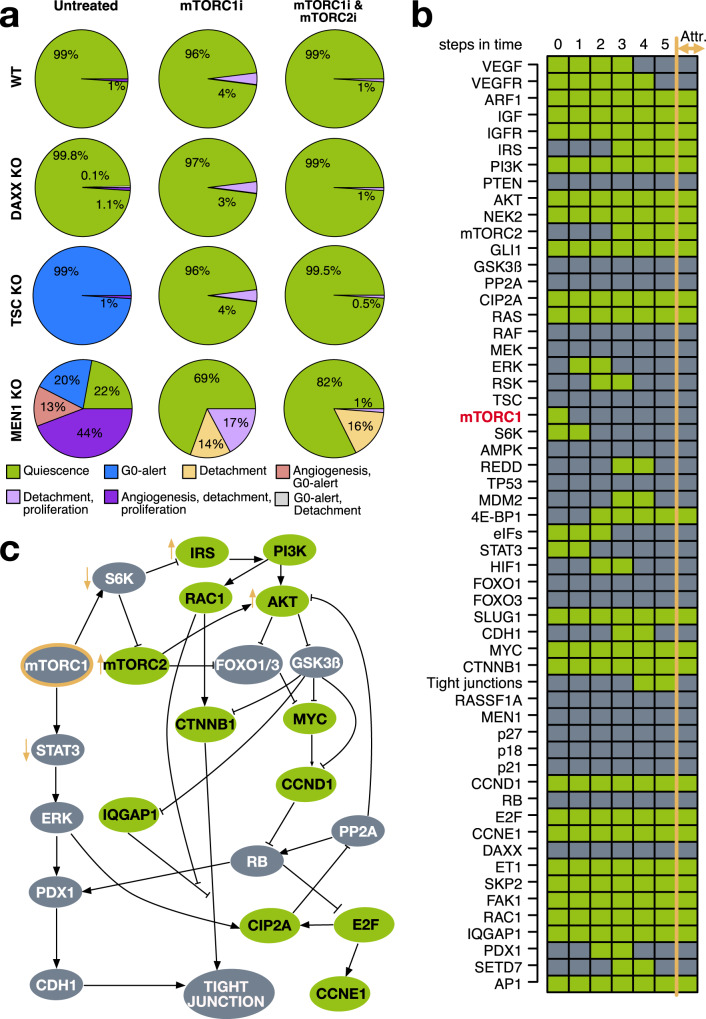
mTORC1 inhibition in PanNETs. mTORC1 knockout and its combination with mTORC2 knockout was simulated and evaluated in both WT and MEN1 loss conditions (**a**). Based on the in-silico results a resistant phenotype could be observed after single mTORC1 inhibition or together with mTORC2. The cascade from the severe cancer phenotype to the mTORC1 resistant phenotype was studied to infer its mechanistic behavior (**b**). Here, genes are listed on the side. mTORC1 is highlighted in red to indicate it was targeted via knockout in the cascade. Node activities are depicted via colored boxes. Green indicates active while gray indicates inactive nodes. A summary of the hypothesized mechanism of mTORC1 resistance is represented in an interaction graph (**c**). Here mTORC1 is highlighted in red. Activities of nodes directly downstream of mTORC1 are depicted with red arrows pointing upwards (activated during resistance) or downwards (inhibited during resistance). The color of the nodes reflects the activities in the attractor cascade.

The knockout simulation was performed on both the WT and the other previously investigated alterations frequently reported in PanNETs, namely DAXX, TSC, and MEN1 loss conditions (Fig. 9a and Supplementary Figs. 11–14). The distribution of the basins of attraction in each condition was estimated by simulating 10,000 randomly drawn starting states. In the WT simulation, we observe an increase from 1% to 4% of the combined phenotype associated with proliferation and detachment. The same scenario regarding the proliferation and detachment phenotypes was also shown in the DAXX and TSC loss conditions (Fig. 9a). However, in the TSC loss condition, an improvement of the phenotypic landscape was observed by reversion of the G0-alert phenotypes to a quiescent after mTORC1 intervention (Fig. 9a and Supplementary Fig. 13). Regarding the MEN1 knockout, we observed a beneficial effect by increasing the quiescent phenotype (from 22% to 69%), accompanied by the loss of the G0-alert related phenotype (Fig. 9a and Supplementary Fig. 14). Still, 17% of the starting states lead to a detachment and proliferation phenotype. Additionally, we did not observe the angiogenic phenotype in any of the mTORC1 interventions (Fig. 9a and Supplementary Figs. 11–14). Overall, based on our simulations, mTORC1 intervention showed a more beneficial effect in the context of MEN1 and TSC mutations.

We interpreted the persistence of proliferative and detachment phenotypes after mTORC1 inhibition in all simulated conditions as emerging resistance to the treatment. Thus, we next studied the potential mechanism leading to resistance after mTORC1 inhibition. To do so, the molecular cascade leading to the resistant mTORC1 phenotype was investigated starting from the aggressive PanNET tumor attractor (Fig. 9b). The cascade was simulated and gave identical results starting from any state of the cyclic attractors depicting an aggressive tumor with proliferative, invasive, and angiogenic traits for all investigated conditions (cyclic attractors reported in Supplementary Figs. 6–9). In Fig. 9b, we report as an example the cascade starting from the first state of the aggressive cancer phenotype of the WT condition. Based on this simulation, we could mechanistically follow the molecular cascade connecting the two attractors (Fig. 9c). The results indicate that after mTORC1 inhibition, there is a simultaneous inactivation of S6K and the reactivation of IRS, which further stabilizes AKT. The inactivation of S6K also affects mTORC2, leading to its activation and further enforcing the positive feedback on AKT. The latter shows downstream effects affecting both tumor proliferation and invasiveness (Fig. 9c), further causing the activation of CTNNB1 and MYC, and inducing the maintenance of active cyclins (CCND1 and CCNE1) (Fig. 9b, c). The same axis also causes the sustained activation of RAC1 and IQGAP1 associated with the complete loss of tight junctions. The loss of STAT3 after mTORC1 inhibition further sustained the inactivation of tight junctions via the loss of PDX1 (Fig. 9b, c). This general regulatory mechanism is further supported by the efficacy in decreasing PanNET progression when double mTOR inhibitors targeting both mTORC1 and mTORC2 are used. In accordance with our hypothesized mechanism, in-silico double inhibition of mTORC1 and mTORC2 also showed a substantial decrease of the resistant phenotype (Fig. 9a and Supplementary Figs. 15–18).

Altogether, we were able to recapitulate observed general behaviors in mTORC1 targeting for PanNETs and additionally predict its efficacy on differently mutated PanNETs. Additionally, we were able to formulate a model-based hypothesis on the regulatory mechanism behind the resistance to mTORC1 resistance in PanNETs. Our results are of particular relevance when considering the targeting of specific patient cohorts harboring different mutations.

## Discussion

Most of the PanNETs are indolent and slow-growing tumors. However, they preserve a malignant potential that is difficult to predict^8^. In addition, a high heterogeneity in tumor biological behavior is observed, ranging from indolent to extremely aggressive^1^. This fact also reflects the difficulty in developing experimental models that can capture the heterogeneous characteristics of PanNETs. BON1 and QGP cell lines have been widely applied to study PanNETs in vitro. However, their relevance as models has been questioned, mainly due to the absence of MEN1 mutations^22^. Nevertheless, a downregulation of MEN1 has been observed in BON1 cells^39^. In the RIP-TAG2 mouse model, PanNETs are induced by expression of the SV40 T-antigen oncogenes in insulin-producing islet $\alpha$-cells^40,41^. This model has been proven to be a valuable prototype for studying the multistep tumorigenesis, with some similarities to the characteristics of human PanNETs^25^. Nevertheless, a major limitation in the understanding of PanNET development and progression is the largely unknown molecular mechanisms and drivers that lead to the development of these heterogeneous tumors. For this reason, we have developed a Boolean model aimed at understanding and unraveling the molecular mechanisms and phenotypes connected with the development and treatment of PanNETs. Our mathematical modeling approach allows us to independently study different mutational landscapes in combination with different therapeutic interventions. Furthermore, we have integrated our modeling framework into a larger systems biology-based setup, where our study includes different classification strategies and comparisons with expression data in both patients and mouse models. Hence, our approach has the potential to complement and integrate the already available phenotypic description of PanNETs from experimental data to capture the extensive crosstalk of PanNET tumorigenesis and progression. Although Boolean network models are rather simple models that rely on extensive simplifications of complex molecular dependencies, several authors have shown that these models are able to capture the same key dynamics of Ordinary Differential Equation (ODE) models describing the same process^42,43^. In this sense, it should be obvious that Boolean networks, cannot predict changes in concentrations due to their binary state values. As with any other manually generated model, a subjective perspective of the modeler cannot be excluded. To account for this, the results of the PanNET model were compared with independent data not used in the model building process. While Boolean network models are much more scalable in size than ODEs^44^, and the presented model with 56 nodes is already large in size, they have the disadvantage that exhaustive simulations become NP-hard^45^. Nevertheless, to estimate the distribution of attractors and thus phenotypes, we estimated basin sizes of up to 1 million starting states. Knowing that very rare attractors may not be found, it is still an adequate method to represent the attractor space (see also Supplementary Figs. 19, 20), also in light of the fact that other authors have chosen the same strategy^46^. On these grounds, we focused on the analysis of rather large differences in the attractor patterns.

The heterogeneity of PanNETs was also captured in our model simulations. We simulated and characterized different mutational landscapes involved in the progression of PanNETs (Fig. 3). We compared our attractor landscapes with “foreign feature selection experiments”, which allow to identify assignments on the gene expression profiles of patient to a cohort selected as “foreign”, e.g., based on the mutation status of DAXX or MEN1. We obtained comparable results regardless of the static (classification) or dynamic (Boolean model) modeling approach. Although the DAXX-mutated patient cohort was closest to the WT cohort in the foreign classification, we could detect a small percentage, showing more aggressive trades within the model, in line with DAXX-mutated tumors also being more likely to be assigned to MEN1-mutated or double-mutated tumors in the foreign feature experiments. These results are consistent with the known indolent behavior of PanNETs, which are known to be slow-growing tumors^8^. Nevertheless, a certain percentage of proliferative and invasive phenotypes can be obtained, which could explain the malignant potential observed in these tumors. In line with this, several authors have shown that staining for Ki-67 at levels higher than 2–10% correlates with metastatic behavior and malignancy grade^47^. Considering specifically the DAXX-mutant tumors, our systems biology approach could capture the conflicting results reported in models and patient cohorts^48^. Two mouse models have been published for DAXX as a driver in PanNETs^49^. In both cases, DAXX mutations were well tolerated in the pancreatic neuroendocrine tissue^49^. Consistent with this, we also observed a predominant quiescent phenotype in DAXX knockout simulations. Additionally, Sun et al.^50^. were able to show that the main effects of DAXX and MEN1 double mutations are driven by MEN1. Again, this result is in accordance with our attractor landscape. Wasylishen et al.^49^. were able to link the loss of DAXX to a permissive transcriptional state that favors the genomic instability and the emergence of additional mutations. Accordingly, DAXX mutations have been associated with chromosomal instability in patient cohorts^51^. The prognostic effect of DAXX in patient cohorts is controversial, with studies showing different effects of the alteration^48^ (see also Supplementary Table 3). These differences have been hypothesized to be due to the histopathologic criteria used to evaluate the DAXX status or related to the metastatic status of the patients included in the cohorts^48^. Based on our classification approaches, we could see that in the multiclass classifier DAXX mutated patients are almost evenly distributed (Fig. 2). However, in the foreign classifier approach, DAXX mutated tumors were mainly associated with other mutated tumors (Fig. 4a, b). Given the relationship between DAXX mutations and chromosomal instability, this may reflect the heterogeneous assignment of DAXX mutated patients to WT and other mutated tumors in our classification strategies. While it is difficult to model chromosomal instability with Boolean models, we could still capture additional adverse effects related to DAXX at the molecular crosstalk level. In conclusion, the results obtained by including patient expression profiles in the PanNETs model simulations highlight the predictive power of the dynamic model, especially in light of the limited information available on the influence of these mutations on the biological behavior of PanNETs. The PanNETs model thus has the potential to capture mutational landscapes that may lead to personalized treatment strategies for PanNET patients.

Interestingly, even in the most severe attractor landscape corresponding to the MEN1 loss simulation, we could not reach the most aggressive phenotype. Instead, we observed a heterogeneous set of phenotypes combining quiescence, proliferation, detachment, and angiogenesis. This reflects the experimental observations of the PanNET tumor heterogeneity^25^.

The cancer driver screening also highlighted general differences in the attractor landscapes when investigating WT and MEN1 loss conditions (Figs. 7 and 8). This in-silico screening was designed and tailored for the present work. However, analyzing initial states that lead to the same attractor is a common practice in systems biology research to evaluate phenotype distribution and identify drivers or treatments^17,21,46^. In general, drivers identified from the WT condition showed more heterogeneous phenotype distributions. This was concomitant with the presence of multiple combinations of phenotypes and high attractor numbers. In contrast, more homogenous phenotypes and low attractor numbers were observed for drivers in combination with a MEN1 loss. These results reflect the experimental evidence that WT tumors are more heterogeneous in their gene expression^25^. Our results also support the hypothesis of multiple-hit tumor development, leading to more severe and stable cancer phenotypes as mutations increase^25^. In addition, we were able to identify MEN1 as a driver from the WT condition, confirming its experimentally established role as a PanNETs driver^13^. In contrast, neither DAXX nor TSC were identified as drivers in our screening (Figs. 7 and 8). This result can be explained by the structure of our screening. In fact, our screening searches for biased activities of nodes that are represented in the basin of the aggressive cancer phenotype. Therefore, the lack of identification of DAXX or TSC as drivers could be interpreted in the context that the activities of these nodes are not highly effective in the induction of an aggressive phenotype. This hypothesis is also supported by the phenotypic landscape of DAXX and TSC loss simulations, which showed less severe biological behavior (Fig. 3). TP53 was identified as a driver in both WT and MEN1 loss simulations. However, when combined with MEN1, the loss of TP53 shows a more severe phenotype, indicating that this gene could be a good candidate as a second hit mutation in PanNETs. In accordance, Yamauchi and colleagues^5^ showed that a TP53 mutation alone did not affect endocrine and exocrine pancreatic tissues in the mouse. In contrast, when combined with a retinoblastoma (RB) mutation, mice developed aggressive PanNET with a Ki-67-labeling index of nearly 30%^5^. Additionally, TP53 alterations were predominantly found in more aggressive types of neuroendocrine neoplasia, highlighting their potential role in the late tumorigenesis^52^. Although RAS is included in our model and KRAS is a well-known tumor marker also for PDAC, we did not find RAS as a first or second hit tumor driver. Consistent with this observation, no RAS mutation is known to be a driver in PanNET patients^53^. This fact supports the hypothesis that different crosstalk affects the development of these two tumor entities, ultimately leading to different progression.

After studying the potential drivers of tumor progression, the focus shifted to targeted therapy. Results from simulations targeting mTORC1, a clinically approved molecular target in PanNETs, confirmed the presence of resistant phenotypes that still induced tumor proliferation and invasion in all simulated PanNETs mutational conditions (Fig. 9). The resistant phenotypes did not exhibit angiogenesis (Fig. 9). The absence of angiogenesis is supported by the anti-angiogenic effect observed in experimental settings for the mTORC1 inhibitor everolimus^38,54,55^. The persistence of the anti-angiogenic effect may be related to the central effect of mTORC1 in regulating angiogenesis via multiple regulatory dependencies^56^. In addition to the presence of the resistant phenotype, a beneficial effect of mTORC1 knockout was observed in MEN1 and TSC loss conditions (Fig. 9a). Whether MEN1 mutations may be clinically associated with a differential response to mTOR inhibitor therapy remains still to be definitively established^57^. Nevertheless, a recent study in 31 patients with advanced PanNETs reported a higher disease control rate, progression-free survival, and time to treatment failure in MEN1 (germline) mutated PanNETs after treatment with everolimus^58^. However, the study included only 6 patients with MEN1 germline mutations and thus the results should be considered as preliminary evidence of a potential beneficial effect of everolimus on MEN1-mutated PanNETs^58^. Our model-based simulations support this beneficial effect of mTORC1 inhibition on PanNETs with MEN1 loss. Loss of TSC induces the hyperactivation of mTORC1^59^. Thus, the beneficial effect of further suppressing mTORC1 in PanNETs with TSC loss is not surprising. Accordingly, mTORC1 inhibition has been suggested as a treatment option in TSC-mutant PanNETs^59^. Again, the small number of cases reported for this mutation in PanNETs does not allow an extensive and valid evaluation of different treatment options. However, Schrader et al.^60^. were able to show encouraging results with everolimus treatment in a patient with a PanNET harboring a TSC mutation. This case study on the mTORC1 targeting could also be extended to individual patients in a more personalized manner by simulating the mutation patterns of a specific patient.

In conclusion, our systems biology approach could support the understanding of PanNET mutational landscapes and predict the effects of therapeutic interventions in specific patient cohorts. Given the limited experimental and patient information on PanNETs, our model encourages the further development of more personalized approaches to understand these tumor entities.

In its current state, our model can support the detection of disease exacerbation by providing a focus on disease drivers and key players in mTORC resistance. However, in its current state, the model will not be able to support early diagnosis, as it was built based on cancer data. However, it could be integrated into the post-diagnosis tumor characterization process by predicting the impact of identified mutations represented in the model. In this direction, the in-silico modeling approach can be easily extended to incorporate new discoveries in the field and be continuously refined. In the future, the model could be extended to study trajectories of disease initiation from healthy tissues as well as for drug screening.

## Methods

### Datasets

In our work, we utilized three publicly available datasets, which are briefly described below.

The GSE117851^61^ dataset contains 47 PanNETs tumor specimens (8 MEN1 mutant, 9 DAXX mutant, 7 double mutant, and 17 wild-type (WT) for the two mutations). The dataset includes human PanNETs specimens, from which RNA was extracted and hybridized to HGU133A2 (Affymetrix) for gene expression profiling.

Second, the GSE73338^25,62^, another human PanNETs dataset grouped by histo-pathological features was used. The dataset contains 95 human PanNETs specimens (63 non-functional, 17 insulinoma, 7 metastasis, 9 normal pancreas) from which RNA was extracted and hybridized to a custom 18.5 K human oligo microarray.

Finally, the GSE73514^25^ contains gene expression data from different tumor stages of the RIP1-TAG2 mice, a genetically engineered PanNET mouse model. Here, we utilized normal (3 samples) and tumor stages (5 samples) that were dissected or isolated from the animal. RNA was extracted and hybridized on Affymetrix GeneChip Mouse Gene 1.0 ST arrays.

For what concerns data processing, the first datasets were already provided as normalized by the original authors. Here GSE117851 was gcRMA normalized and GSE73338 was normalized according to Huber et al.^63^. Instead, GSE73514 was RMA normalized.

### Classification experiments

We conducted two types of experiments based on binary classification models (further on referred as base classifiers). In both cases linear, support vector machines (SVM) were chosen as base classifiers. The training process of these binary SVMs were coupled to an internal feature selection^64^. The 200 top features according to the Threshold Number of Misclassification (TNoM) scoring^65^ were chosen. All classification experiments were conducted with R-packages ORION and TunePareto^66^.

#### Multi-class classifier

A multiclass classifier experiment was performed to investigate the potential of different PanNETs with neuroendocrine properties datasets to be classified. Multiclass classifier applied was a one-against-one SVM. To construct the decision over all classes to be separated, a base classifier (SVM) is computed for each pair of classes. Each classifier predicts one of two classes, and then a majority vote is performed for the ensemble decision. In case a consensus on the majority is not found, multiple solutions are computed based on the equally voted classes. This was the case for the GSE117851 dataset for which we provide the complete set of confusion matrices as Supplementary Fig. 21. The output of the multiclass classifier was presented in the form of a confusion matrix, where each entry represents the probability of being assigned in the corresponding class. The experiments were conducted as leave one out cross-validation (CV) analyses. Two datasets were analyzed using this approach: GSE117851^61^, and GSE73338^25,62^.

#### Foreign classification experiments

A foreign classifier approach (see also Supplementary Fig. 8) was designed to compare and integrate the dynamic model-based predictions^67^. Here, an external, or foreign class, to the one on which a binary classification experiment is performed is reclassified using the decision boundaries of that binary classification. The assignment of foreign classes to binary classification tasks for differently mutated patient cohorts was interpreted as a measure of similarity or purity between the different cohorts. Hence, a foreign class assigned with higher frequency to one of the original classes is assumed to have certain similarities in the molecular expression profile to one of the two classes. By performing this combination of binary classification experiments and reclassification of the foreign class for all possible combinations of classes in the dataset, it is possible to infer a general interpretation of the similarities of the expression profiles present in the examined dataset. The distribution of assignments is then compared to the similarity of attractor landscapes.

We applied this strategy to investigate different driving mutations in PanNETs, creating a parallel between phenotypic landscapes hypothesized by model simulations and common features shared by cohorts of differentially mutated patients. In the specific scenario, the GSE117851^61^ dataset was utilized. For the binary classification tasks, the analysis was performed via SVMs, focusing on 200 top features based on the Threshold Number of Misclassification (TNoM) scoring^65^ determining the informativeness of a feature. Based on the TNoM, the top 200 features with the smallest error rates were used for training the binary SVM. The experiments were conducted as leave one out CV.

### Boolean network models

Boolean network models are among the simplest dynamic models. Their simplicity arises through the assumption that nodes representing genes or proteins can have two activity states^15,16^. They are either active (ON/1) or inactive (OFF/0). Thus, the state of a network at a given point in time is defined by the activity of all nodes at that point in time^15,16^. Qualitative information derived from the literature is sufficient to construct the regulatory interactions of this type of model^16^. Therefore, this information is transferred into mathematical formulas using logical connectives such as AND, OR, and NOT^16^. Together, this set of mathematical formulas, called Boolean functions, is used to simulate the model. To do so, all Boolean functions are updated simultaneously to create a state transition. This process is repeated until the system enters a sequence of recurrent state(s), called attractor(s). Attractors describe the long-term behavior of a system and can be associated with biological phenotypes^15,16^. Attractors can be either single states (fixed points) or cyclic sequences of states. All initial states leading to the same attractor are part of its basin of attraction, which depicts the relevance of that attractor to the whole system^16^.

The exhaustive simulation of the dynamics of a Boolean network is an NP-hard problem^45^. Using the R package BoolNet^68^, such an exact determination of basin sizes is limited to networks with a maximum of 29 genes. For larger networks, such as the model presented here, a random sampling of states is used instead. The convergence of the basin sizes obtained with increasing sample size is highlighted in Supplementary Figs. 19, 20.

### PanNET model construction

The model presented is based on an extensive literature search. The general modeling strategy followed that presented in Ikonomi et al.^69^. First, we collected review papers on PanNETs to get an overview of the pathways involved and common germline mutations. Based on this, we collected information on regulatory interactions for the mTOR, MAPK, and PI3K/AKT pathway, as well as the genes and proteins that are involved in MEN1 or DAXX/ATRX signaling and their crosstalk. Moreover, we considered regulations of the cell cycle, for cell adhesion and angiogenic traits to include well-known phenotypes of PanNET tumors. Whenever possible, we considered regulations that are described for pancreatic tissue. To ensure that our model includes reciprocal crosstalk between the individual nodes, we also extend our literature data collection by taking the curated database MetaCore^TM^ (Clarivate) into account. The Boolean functions of the established model can be found in Supplementary Table 1 and are available as an SBML-file in our git repository (https://github.com/sysbio-bioinf/PanNET). In addition, we provide a detailed description of the underlying mechanistic regulations in Supplementary Table 1, and a list of the cellular localization and function of our nodes in Supplementary Table 2.

### Model simulation

Model simulations were performed using the R-package BoolNet^68^. We used synchronous updating of all Boolean functions^16^. We simulated different mutational landscapes of PanNETs by constantly setting the node involved in the change to 1 or 0^16^. This relates to an experimental knockin or knockout. For this reason, such perturbation experiments are of interest for predicting intervention targets and further guiding future laboratory experiments.

To evaluate and compare the obtained attractor patterns with publicly available experimental data for these mutations, we selected the activity of certain nodes in the attractors to assign a particular phenotype. To do so, we focused on describing phenotypes that are also described in experimental results: proliferation, quiescence, Gap zero (G0)-alert, detachment, and angiogenesis. To evaluate the proliferation status, we considered the activity of cyclin E1 (CCNE1) and E2F transcription factor (E2F), which mark the entry into the synthesis (S)-phase. Thus, the absence of their activity in the attractor pattern was assigned to quiescence. Attractors with any activity in CCNE1 and E2F but with mTORC1 activity were assigned to a G0-alert phenotype. This state was first described in stem cells and related the activity of mTORC1 to an “alert” state that primes cells to enter the cell cycle^70,71^. We interpret the presence of this combination of attractor states as priming for proliferation, and therefore we distinguish this state from complete quiescence. Detachment was evaluated based on the inactivity of the node tight junctions. Instead, angiogenesis was considered to be present when both Vascular endothelial growth factor (VEGF) and Vascular endothelial growth factor receptor (VEGFR) were active in the attractor landscape (Supplementary Fig. 3).

Finally, we could quantify the presence of these phenotypes, taken singly (or in combination) in our attractor landscape, based on the corresponding basin of attraction of the attractors in which they appeared. The basin of attraction represents the number of initial states that end up in a certain attractor after simulation^16^. Hence, the larger the basin, the more the attractor is preponderant in the landscape, and the more the phenotype is relevant^72^. For a given input condition, we obtained the attractor landscape of the PanNETs model by randomly sampling 10,000 initial states. Note that our result was not significantly altered by the sampling size of the initial states (Supplementary Figs. 19 and 20). In accordance, Cho and colleagues^46^ also showed similar basin behavior by sampling the same number of tates in a large model of colorectal cancer.

### Stability assessment and scale freeness

We applied noise to our PanNETs Boolean network model to evaluate the robustness—and thus the significance—of our model and the applied simulations. We analyzed the impact of noise in the model and compared it to randomly generated Boolean networks.

A total of 1000 random networks were generated using a “TestNetworkProperties” function that calls the generateRandomNKNetwork() function in the R package BoolNet^68^. The parameters *n* and *k* of this function refer to the total number of nodes in the network (*n*) and the number of inputs of a given node (*k*), respectively. These paramters are selected according to their values in the PanNET network to ensure a comparable topology.

To apply noise, we utilized the widely applied random bit flips (the assignments of selected nodes were toggled from 1 to 0 and vice versa)^73^. Once a bitflip is applied to a network state, the corresponding successor state of both the original and the perturbed state is computed. The distance between the two successor states is then measured using the normalized Hamming distance, which measures the number of different bits in two state vectors. The distance is an indicator of the ability of the Boolean network to maintain its functionality under noisy conditions. Thus, a Hamming distance of zero indicates that the applied mutation has no effect on the evaluated network behavior. The procedure was repeated for 1000 randomly drawn states. The number of bit flips for the perturbation was set to one. Finally, the results were compared with those of 1000 randomly generated Boolean networks. For this computationally intensive test, we computed a *p*-value to evaluate whether our null hypothesis“ the Hamming distance for the constructed network is greater than or equal to the distance for the random networks” could be rejected. We considered *p* < 0.05 to be significant. This test procedure is included in the R-package BoolNet^68^.

Scale freeness is another relevant property of biologically motivated networks. If a Boolean network has a scale-free network architecture, it can be described by the power-law distribution $P(k)\propto \,{k}^{-\alpha },$ where $\alpha$ is the power-law scaling parameter and k is the number of edges in the network. To identify scale-freeness, we tested whether the power- law distribution can plausibly describe the degree distribution of the network (*p* > 0.1) by using the R-package poweRlaw^74^. For the model presented here, a *p*-value of 0.68 was obtained for the network without time delays. The corresponding degree distribution is shown in Supplementary Fig. 1.

### Model validation

Validation of the attractor activities associated with the most aggressive phenotype showing proliferation, detachment, and angiogenesis was performed both by literature search and comparison with expression data (Fig. 5 and Supplementary Table 4). The gene expression levels of normal and tumor tissues derived from RIP-TAG2 mice in the GSE73514 dataset^25^ were binarized by a threshold defined by a ROC curve using the R-package pROC^75^. According to this threshold, expression levels above the threshold were considered as active and expression levels below the threshold were considered inactive.

### Tumor driver screening

The tumor driver screening was based on the analysis of the basin of attraction of the aggressive attractor pattern. Here, we designed a strategy based on the hypothesis that nodes with a biased activity in the basin of attraction of a phenotype/attractor of interest could ultimately influence the systems towards that attractor when perturbed. An example of the approach in a small ‘toy model’ is depicted in Supplementary Figure 10. We evaluated the activity of nodes in our network in the 100 million randomly drawn initial states of the basin of attraction that lead to the aggressive attractor pattern in both the WT and MEN1 simulation setups. Here, we examined activities that would exceed one standard deviation from the mean in both directions and considered these initial states as potentially relevant to the final attractor landscape. Hence if a node has a low probability of being active in the start states leading to the severe attractor pattern, we will consider this as a hint for that this node will be lost during tumor progression. For each node identified as a driver, the corresponding attractor landscape was computed via the knockin/knockout of the driver node. Again, simulations were performed starting from 10,000 randomly sampled start states under synchronous update schemes. Drivers whose activity was unaltered between quiescent and aggressive attractor patterns were not further evaluated for the attractor landscape (namely AMP-activated protein kinase (AMPK) and Tumor protein 53 (TP53) in the WT condition). In other words, a node selected as a low active driver, but always inactive in quiescent and aggressive attractor patterns, is not considered to influence the overall behavior. An exception was made for the TP53 knockout in the WT condition, where the attractor landscape simulation was performed as a comparison to the TP53 knockout in the MEN1 loss condition.

### Statistics

For statistical analysis, the Wilcoxon rank-sum test was performed using the open-source statistical software R (https://www.r-project.org). Here, *p*-values of 0.05 were considered statistically significant. The data were also visualized in R.

### Reporting summary

Further information on research design is available in the Nature Research Reporting Summary linked to this article.

## Supplementary information

Supplement

Reporting Summary

## Data Availability

All data supporting this research are included in the published article, are included in its supplement file, or are available on git (https://github.com/sysbio-bioinf/PanNET).

## Code availability

All simulations have been performed via the software R (https://www.r-project.org) (Version 4.0.0). Specific packages and information to reproduce the simulations are provided within the methods and supplement of the manuscript, or are available on git (https://github.com/sysbio-bioinf/PanNET).
