# Supplementary material for: A systems biology approach to define mechanisms, phenotypes, and drivers in PanNETs with a personalized perspective
Source: NPJ Syst Biol Appl. 2023 Jun 3;9:22. doi: 10.1038/s41540-023-00283-8 (PMC10239456; doi:10.1038/s41540-023-00283-8)
Supplement: Supplementary file 1 — Supplement [file 41540_2023_283_MOESM1_ESM.pdf]

A systems biology approach to define mechanisms, phenotypes, and drivers in PanNETs with a personalized perspective – Supplementary Information –

Silke D. Werle [1,†], Nensi Ikonomi [1,†], Ludwig Lausser[1,2],
Annika M. T. U. Kestler [1], Felix M. Weidner [1], Julian D. Schwab [1], Julia Maier[1,3], Malte Buchholz [4],
Thomas M. Gress [4], Angelika M. R. Kestler[5], and Hans A. Kestler [1,*]

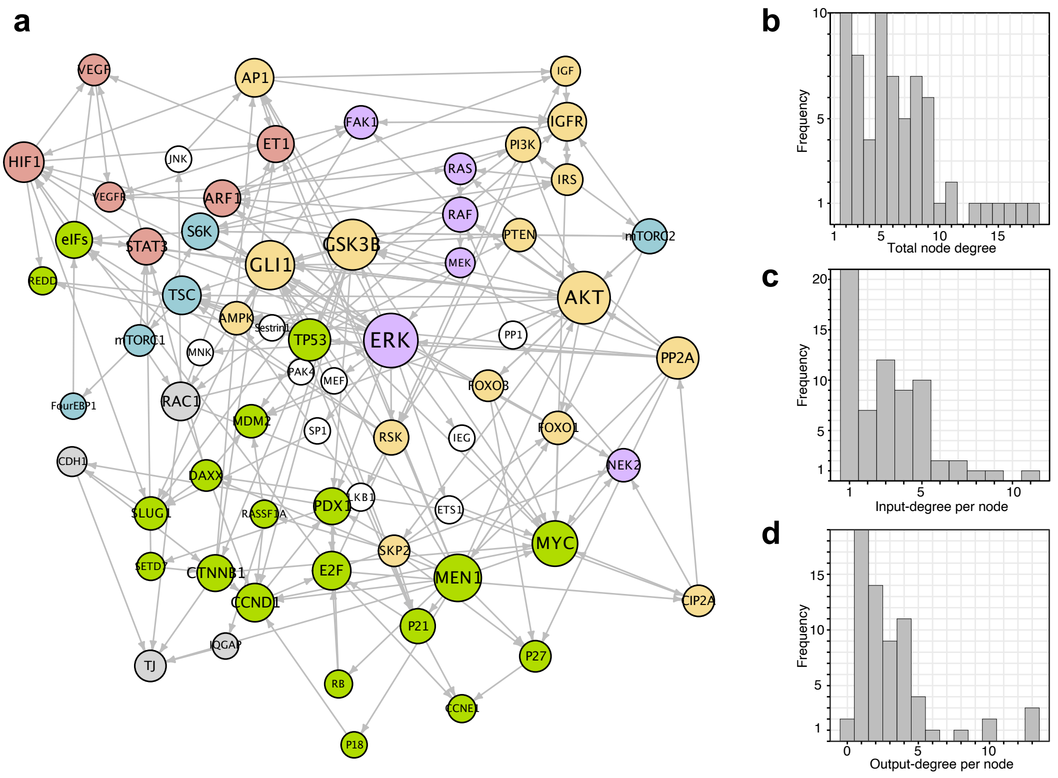

Supplementary Figure 1: Model scale-freeness. (a) Automatically generated interaction graph using the R-package igraph [1]. The node size correlates with the z-score transformed total node degree of the nodes within the PanNET model. Colors depict pathway association - red = angiogenesis; purple = MAPK signaling; yellow = PI3K/AKT signaling; blue = mTORC signaling; grey = cell adhesion; green = cell cycle; white = multiple pathway associations. (b) Total node degree distribution of the PanNET model. (c) Input-degree distribution of the PanNET model. (d) Output-degree distribution of the PanNET model.

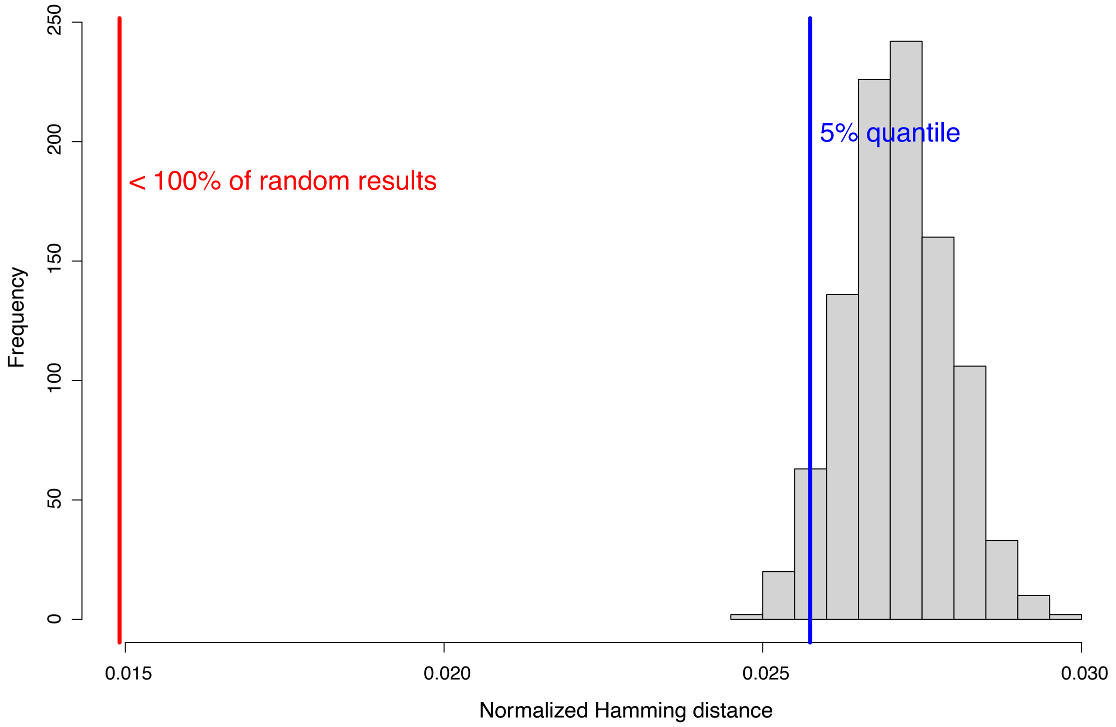

Supplementary Figure 2: Stability assessement of the PanNETs model. One thousand randomly drawn states of the HSC model were mutated by bit flip and their successor states were computed. The successor states of the mutated and the original states were then compared using the normalized Hamming distance (red line, intercept at 0.0149). The same analysis was performed for 1,000 randomly generated networks of the same size (histogram). The blue line shows the 5% quantile (intercept at 0.0257). The calculate p-value between the model and the randomly generated networks is below 0.05. Hence the model is significantly more robust than noise.

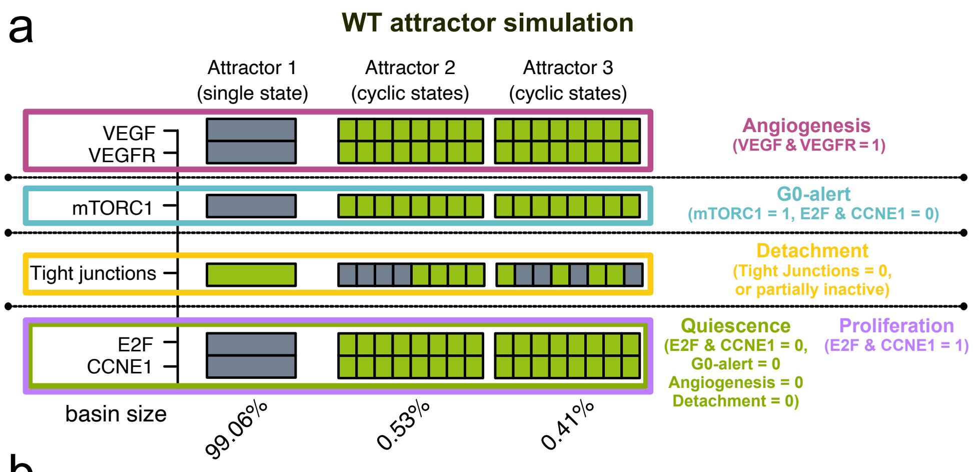

b

Phenotype	VEGF	VEGFR	mTORC1	Tight junctions	E2F	CCNE1
Quiescence	0	0	0	1	0	0
G0-alert	0	0	1	1	0	0
Proliferation	0	0	0	1	1	1
Detachment	0	0	0	0	0	0
Angiogenesis	1	1	0	1	0	0
G0-alert & detachment	0	0	1	0	0	0
G0-alert & angiogenesis	1	1	1	1	0	0
Proliferation & detachment	0	0	0/1	0	1	1
Proliferation & angiogenesis	1	1	0/1	1	1	1
Detachment & angiogenesis	1	1	0	0	0	0
G0-alert & detachment & angiogenesis	1	1	1	0	0	0
Proliferation & detachment & angiogenesis	1	1	0/1	0	1	1

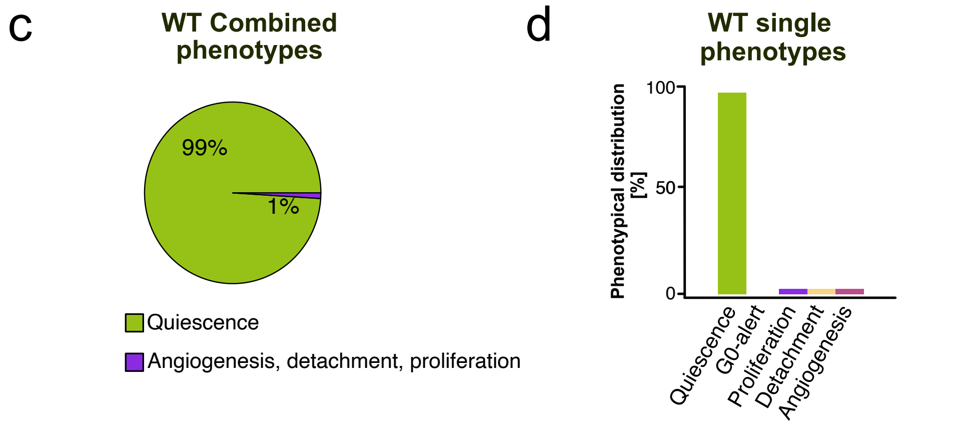

Supplementary Figure 3: Example of attractor interpretation and visualization. The interpretation of read-out phenotypes and their representation is presented based on the WT simulation of the PanNET model. Nodes used to interpret the PanNET model phenotypes are extracted from the attractor pattern (a). On the side of each node, the criteria for assigning the specific phenotypes are reported. The estimated basin sizes are reported under each attractor. In the attractor pattern, green boxes indicate activation, while dark purple ones indicate inactivation. The phenotype encoding is also exhaustively depicted in a tabular form (b). Phenotypes present in each attractor then are combined and weighted based on their basin of attraction (c). In the pie chart, 99% of the phenotype is represented by quiescence, represented by the single state attractor. Instead, the two cyclic attractors cover 1% of the basin connected to angiogenesis, detachment, and proliferation. The two combined phenotypes can be broken down independently into single phenotypes (d). Again percentages for each phenotype overall the attractor landscape corresponds to the estimated basins of attraction. In this case, overall the attractor landscape, 99% is represented by quiescence, while angiogenesis, detachment, and proliferation cover the 1%. This visualisation is considered more intuitive when comparing the effect of many perturbations on the attractor landscape.

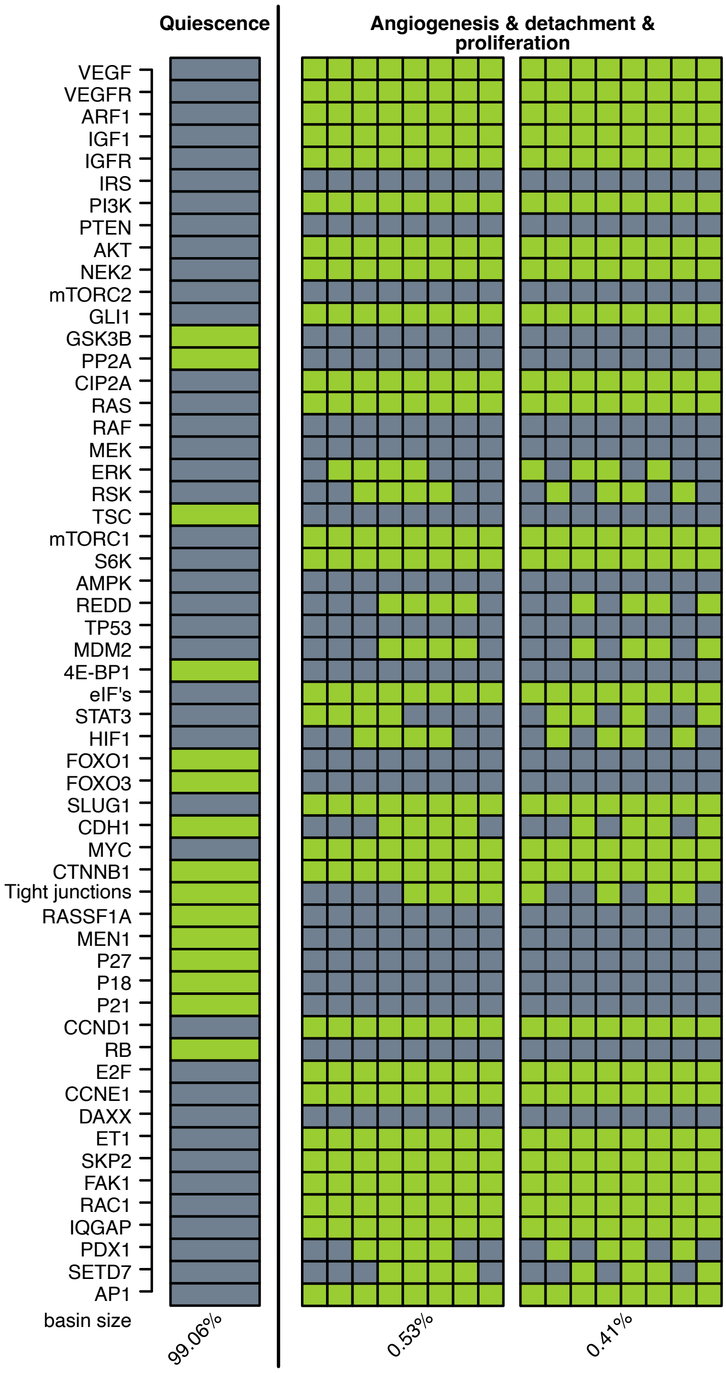

Supplementary Figure 4: Unperturbed attractor patterns of the PanNETs model. The attractor landscape of the unperturbed (WT) PanNETs network is reported. Attractors are grouped based on their matched phenotypes. Below each attractor the basin size is reported. Activities are reported in the colored boxed. Here, green indicates active (1) and grey indicates inactive (0) genes/proteins.

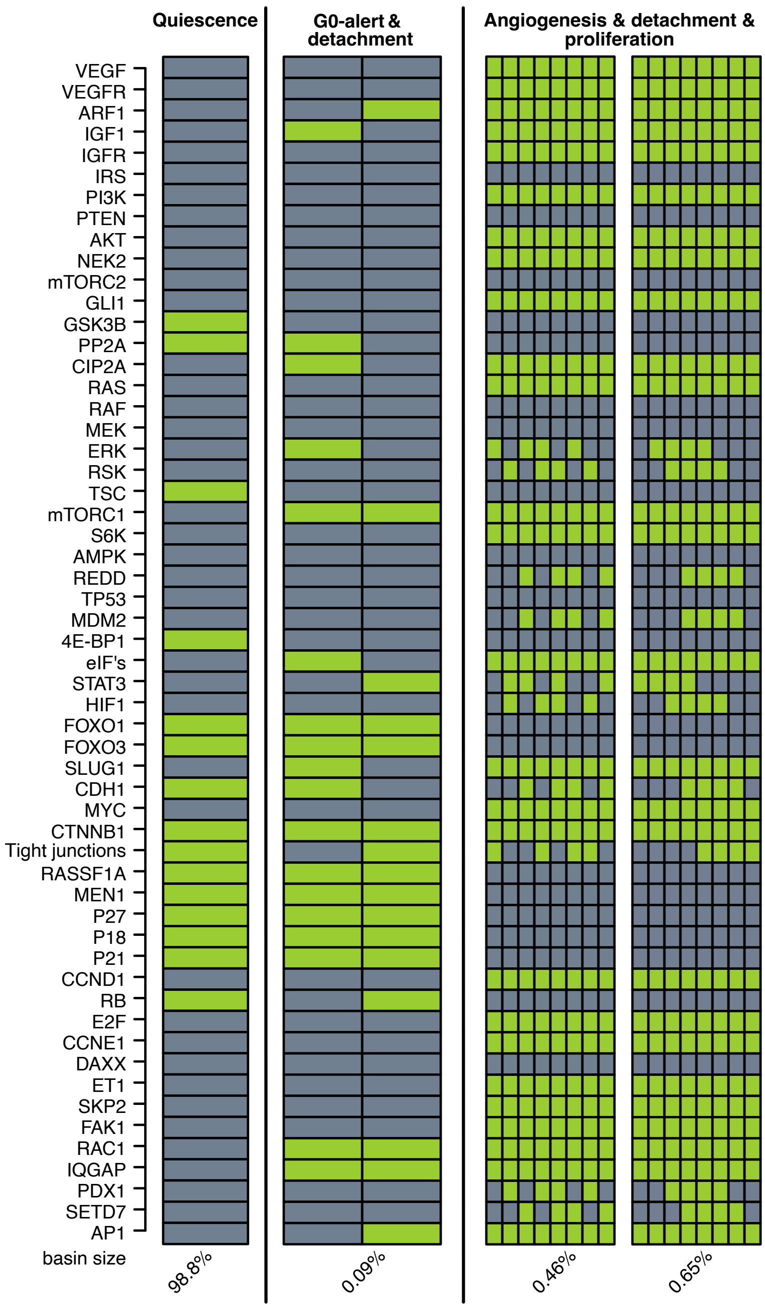

Supplementary Figure 5: DAXX loss attractor patterns of the PanNETs model. The attractor landscape of the DAXX loss (DAXX KO) PanNET network is reported. Attractors are grouped based on their matched phenotypes. Below each attractor the basin size is reported. Activities are reported in the colored boxed. Here, green indicates active (1) and grey indicates inactive (0) genes/proteins.

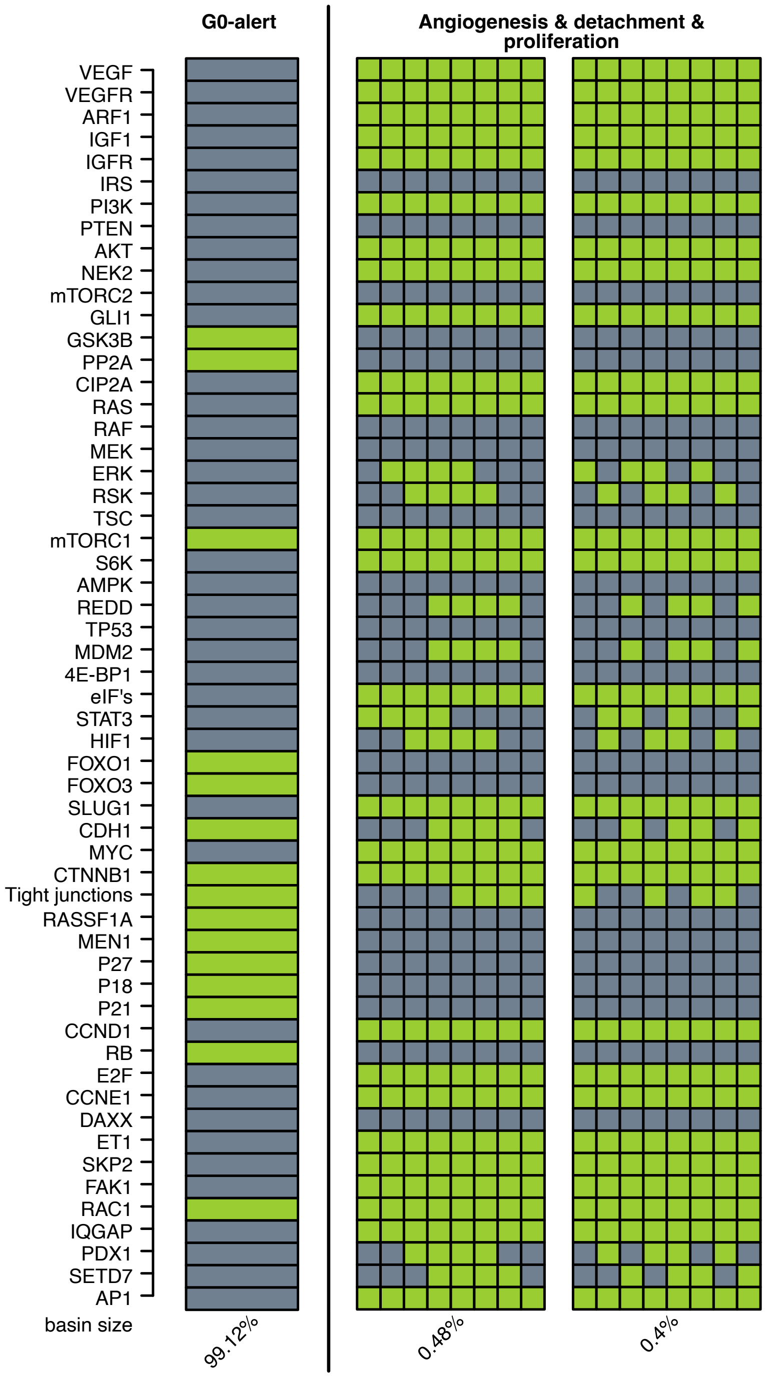

Supplementary Figure 6: TSC loss attractor patterns of the PanNETs model. The attractor landscape of the TSC loss (TSC KO) PanNET network is reported. Attractors are grouped based on their matched phenotypes. Below each attractor the basin size is reported. Activities are reported in the colored boxed. Here, green indicates active (1) and grey indicates inactive (0) genes/proteins.

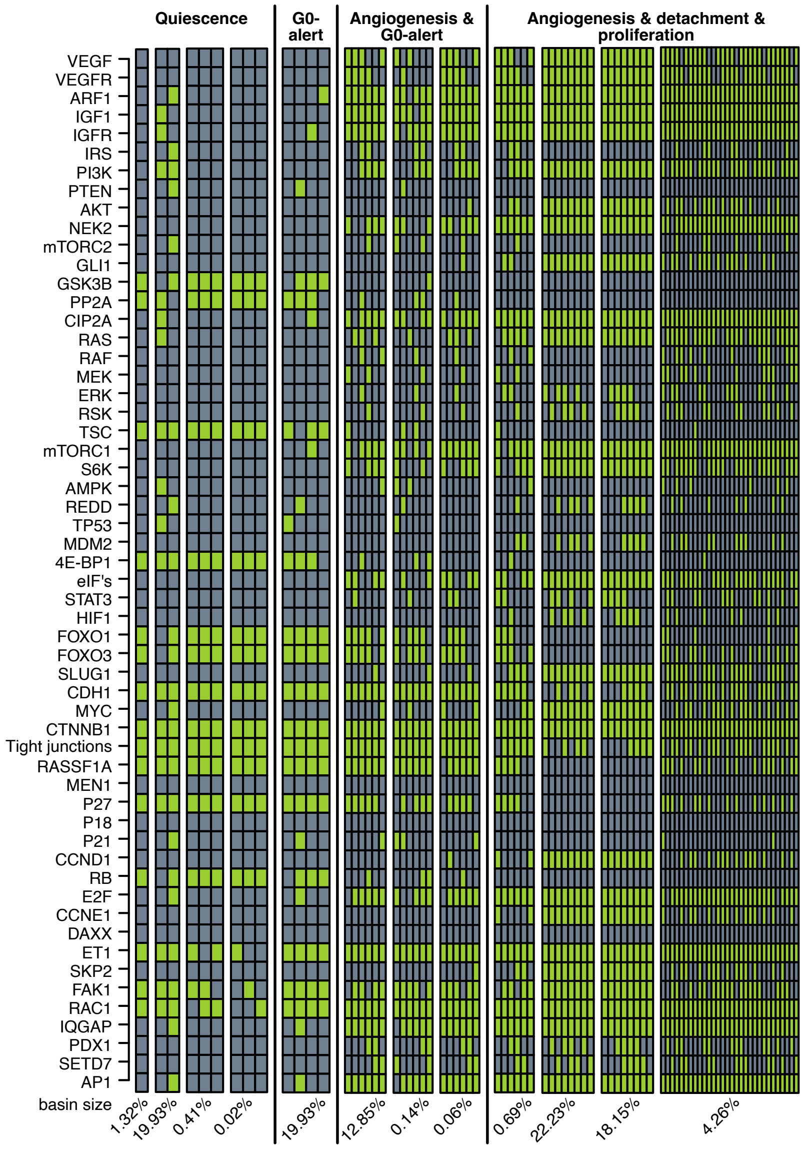

Supplementary Figure 7: MEN1 loss attractor patterns of the PanNETs model. The attractor landscape of the MEN1 loss (MEN1 KO) PanNET network is reported. Attractors are grouped based on their matched phenotypes. Below each attractor the basin size is reported. Activities are reported in the colored boxed. Here, green indicates active (1) and grey indicates inactive (0) genes/proteins.

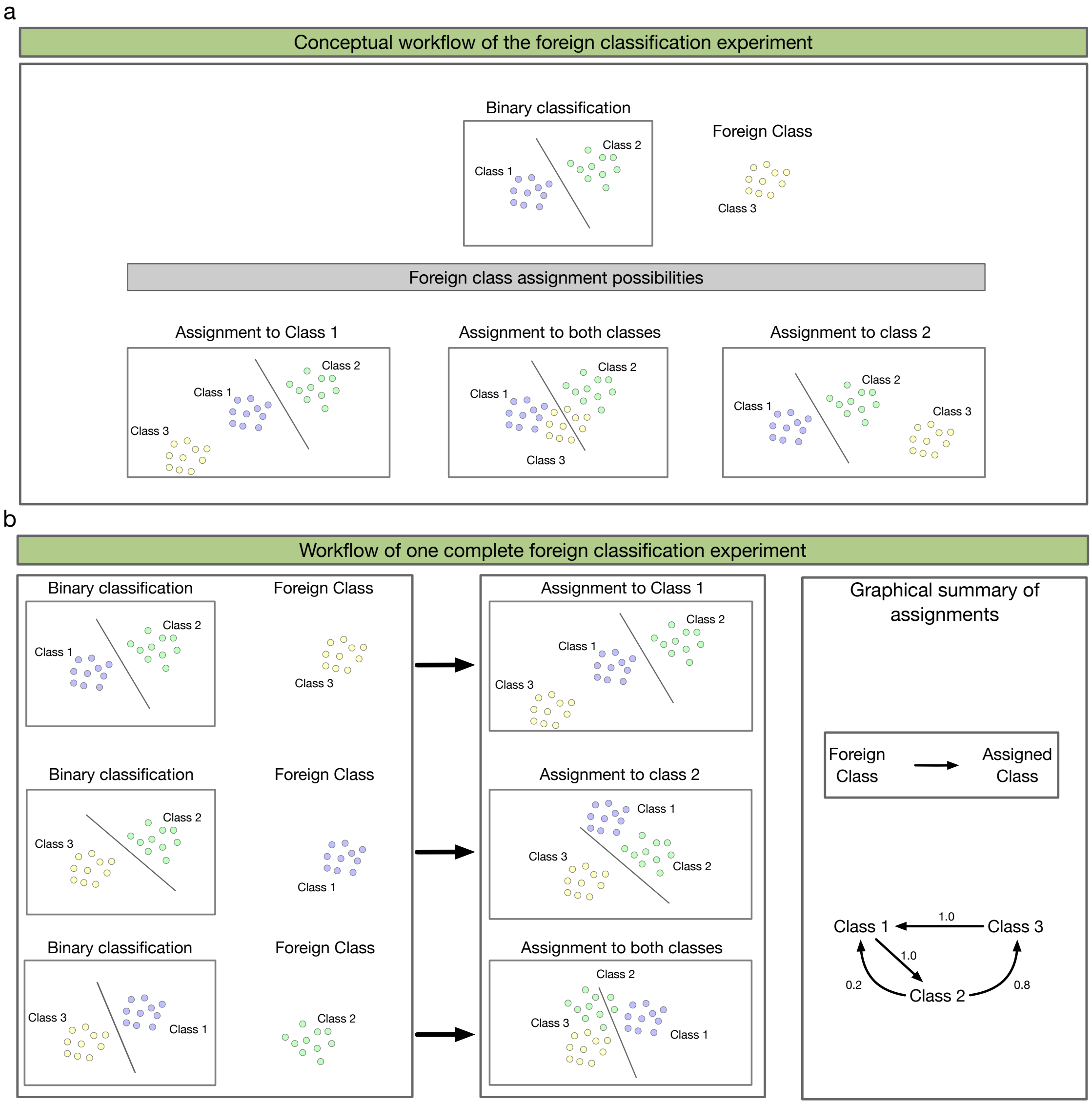

Supplementary Figure 8: Workflow foreign classification. The structure of the foreign classification experiments is depicted. (a). First two classes in a dataset are chosen to perform a linear classification. A third class is left out, and represents the foreign class of the experiment. The latter is assigned to one of the two classes, or both of them, based on the linear classification previously performed (lower pannel). The workflow of the of a complete foreign classification experiment is depicted below, in pictorial dataset composed of three classes (b). All classes in the are iteratively used in couples to perform the first binary classification. Each foreign class is assigned based on the the first linear classification. Finally, the results of the experiment are summarized graphically in an interaction graph, where the direction of edges indicates the assignment of the foreign class to its assigned class. Numbers over the edges indicate the assignment fraction of the foreign class in exam.

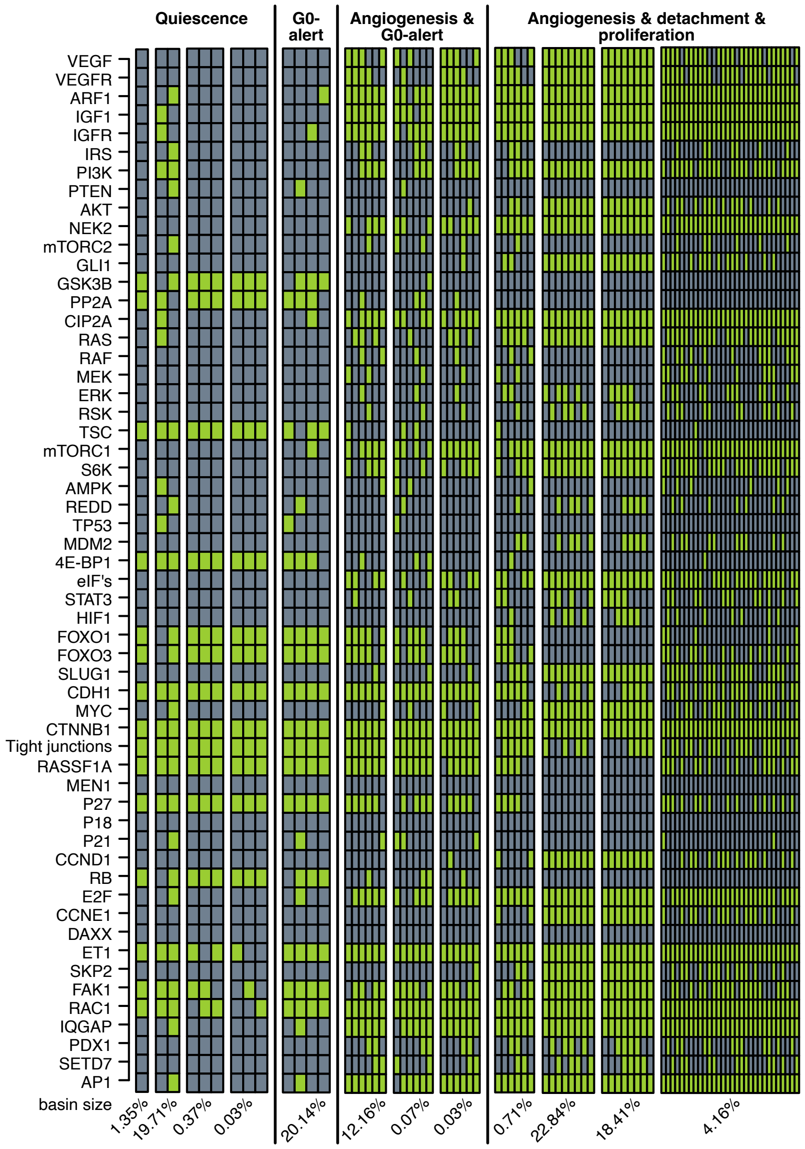

Supplementary Figure 9: MEN1 and DAXX loss attractor patterns of the PanNETs model. The attractor landscape of the MEN1 and DAXX loss (MEN1 & DAXX KO) PanNET network is reported. Attractors are grouped based on their matched phenotypes. Below each attractor the basin size is reported. Activities are reported in the colored boxed. Here, green indicates active (1) and grey indicates inactive (0) genes/proteins.

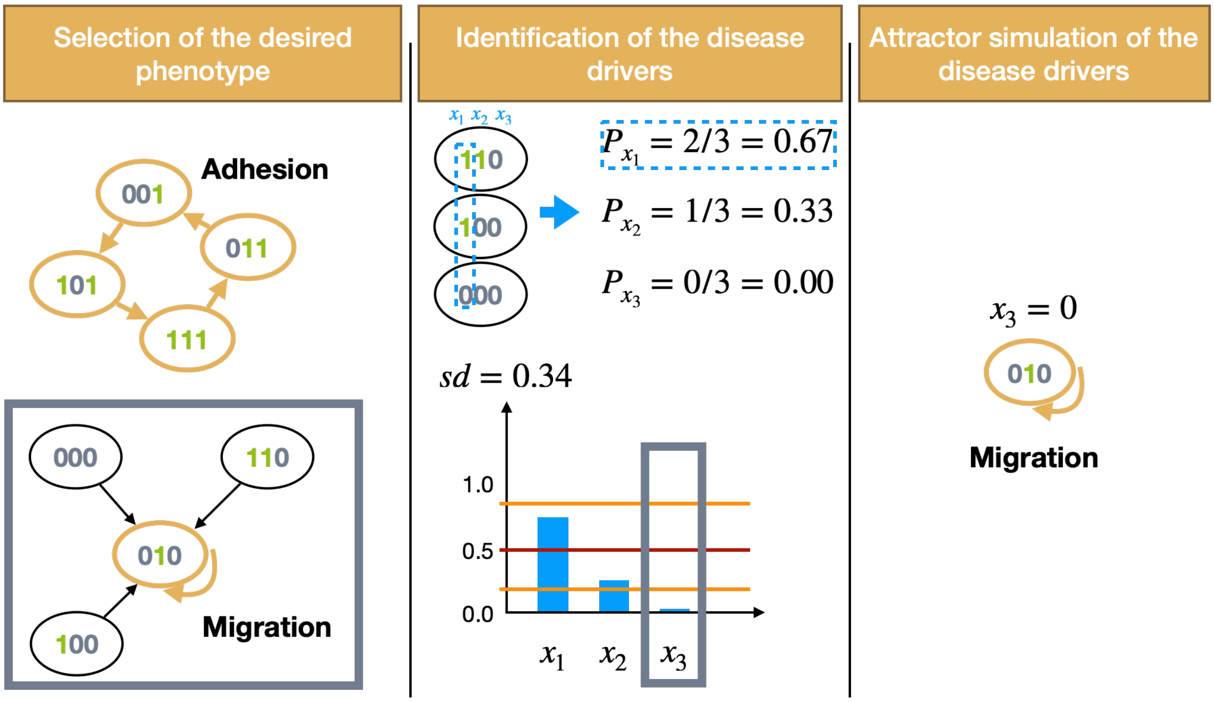

Supplementary Figure 10: Brief visualization for the disease driver screening. A toy model of the disease driver screening strategy is depicted. First an attractor of interest is selected, in this case the migration inducing one (left panel). The basin of attraction of the attractor of interest is then screened and frequency of activities of each node is calculated (middle panel). Then, nodes whose frequency of activities is higher or lower of one standard deviation are selected. Finally, the corresponding perturbation is simulated and the attractor related phenotype is evaluated (right panel).

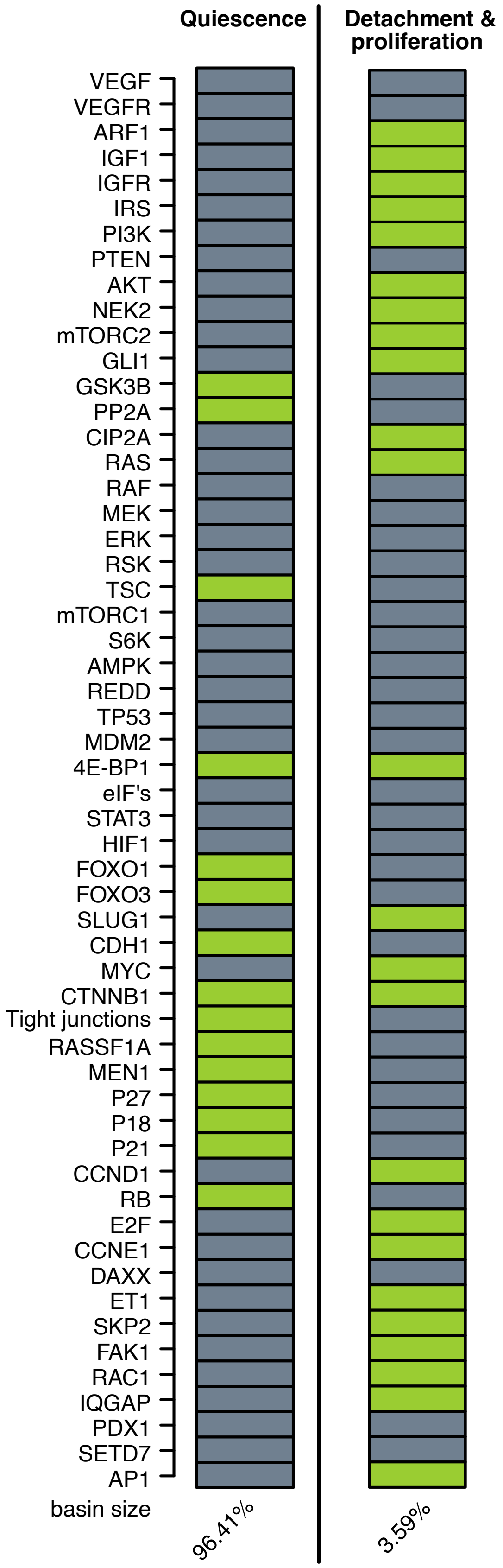

Supplementary Figure 11: mTORC1 intervention attractor patterns in WT PanNETs. The attractor landscape of the mTORC1 intervention on the WT PanNET network is reported. Attractors are grouped based on their matched phenotypes. Below each attractor the basin size is reported. Activities are reported in the colored boxed. Here, green indicates active (1) and grey indicates inactive (0) genes/proteins.

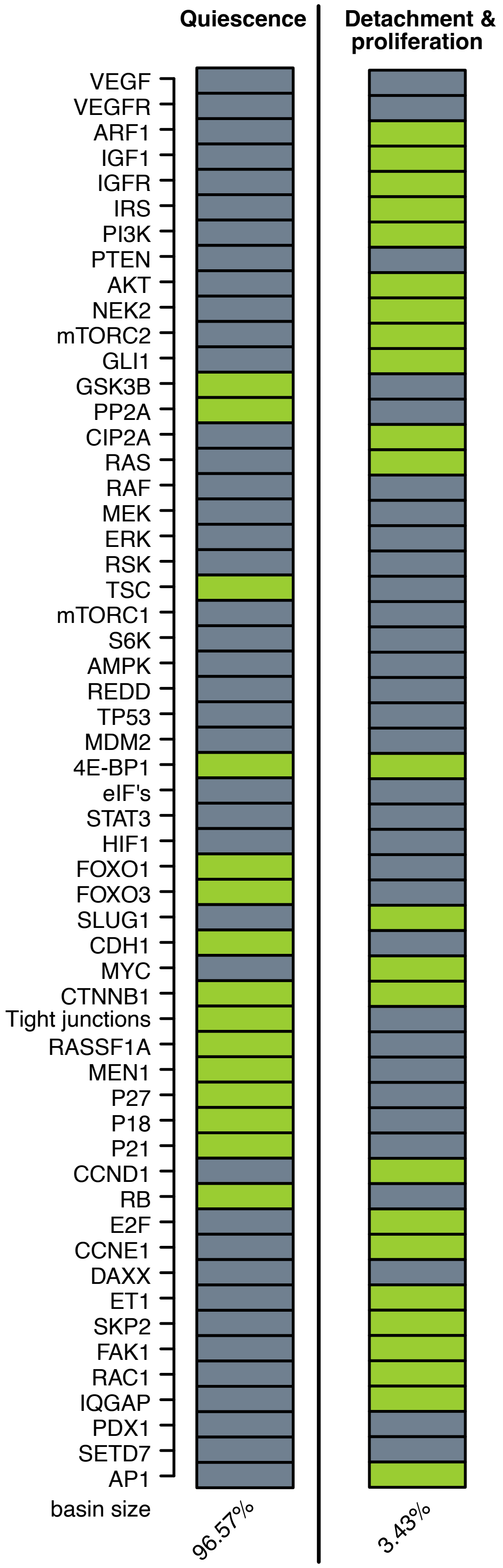

Supplementary Figure 12: mTORC1 intervention attractor patterns in DAXX loss PanNETs. The attractor landscape of the mTORC1 intervention on the DAXX loss PanNET network is reported. Attractors are grouped based on their matched phenotypes. Below each attractor the basin size is reported. Activities are reported in the colored boxed. Here, green indicates active (1) and grey indicates inactive (0) genes/proteins.

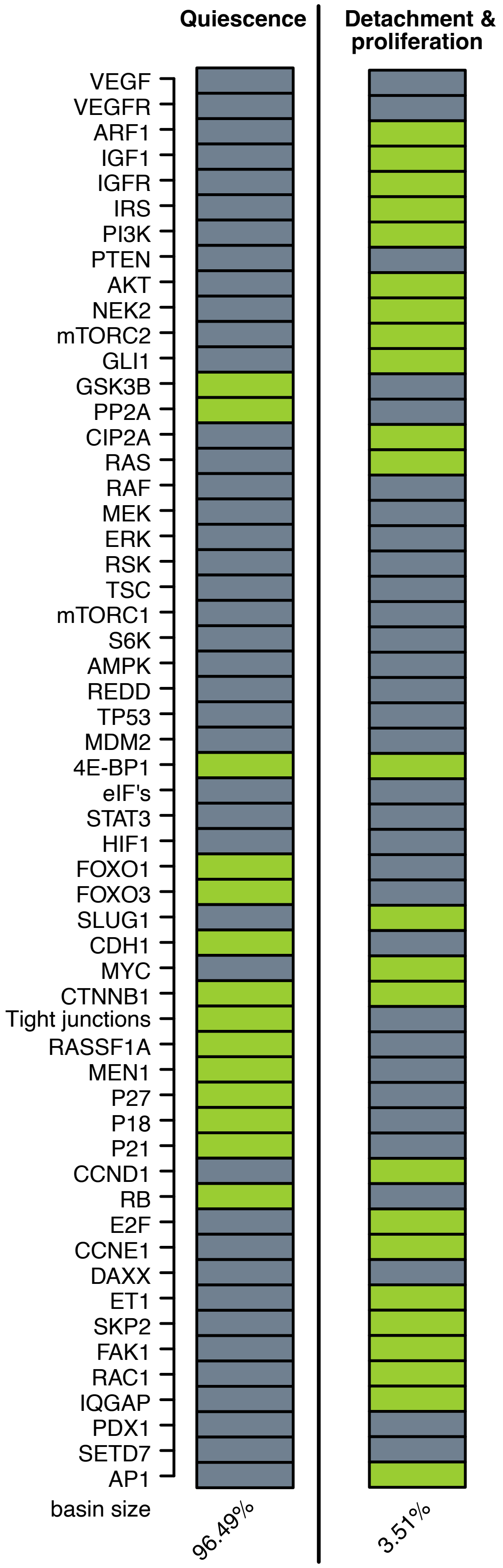

Supplementary Figure 13: mTORC1 intervention attractor patterns in TSC loss PanNETs. The attractor landscape of the mTORC1 intervention on the TSC loss PanNET network is reported. Attractors are grouped based on their matched phenotypes. Below each attractor the basin size is reported. Activities are reported in the colored boxed. Here, green indicates active (1) and grey indicates inactive (0) genes/proteins.

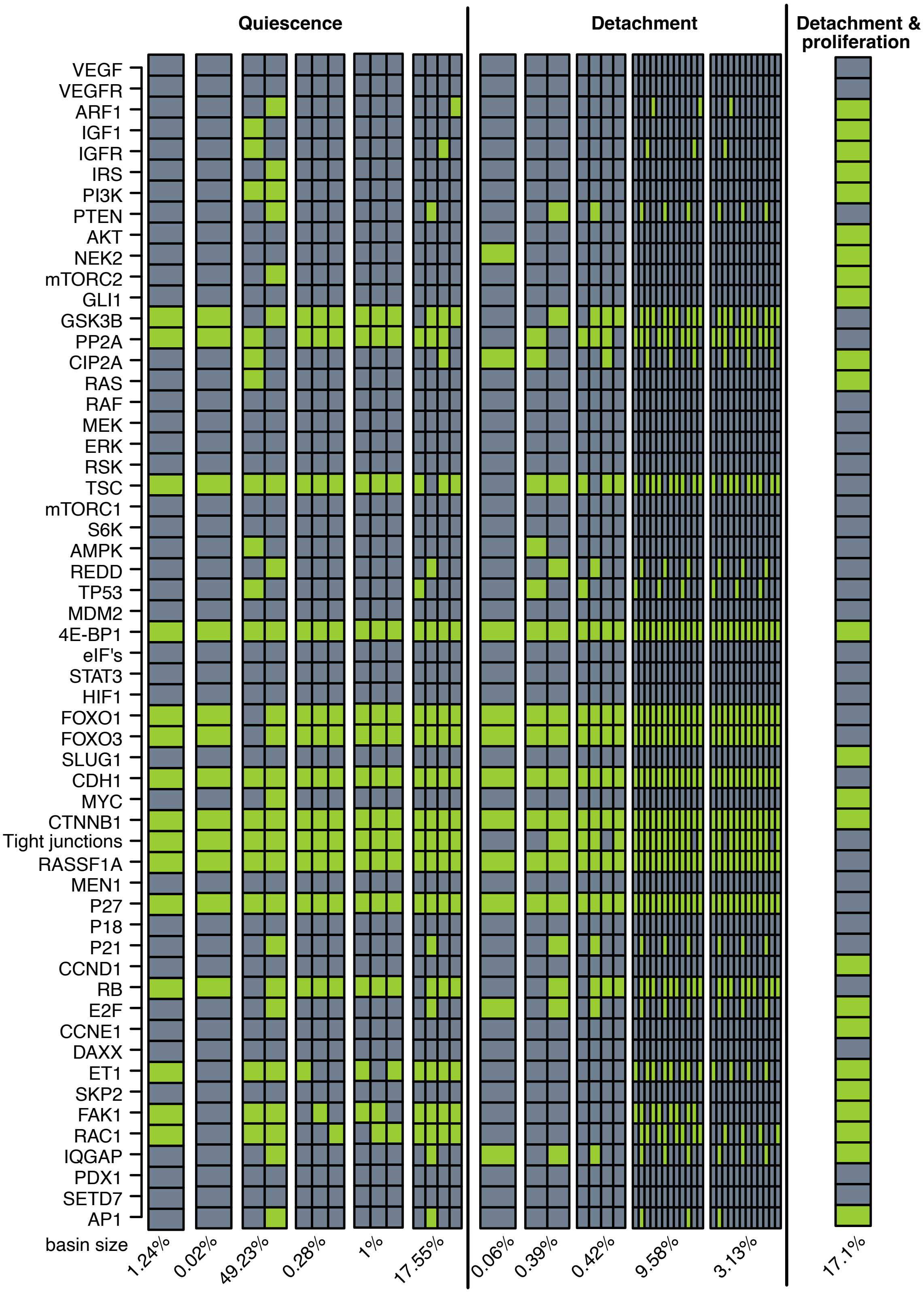

Supplementary Figure 14: mTORC1 intervention attractor patterns in MEN1 loss PanNETs. The attractor landscape of the mTORC1 intervention on the MEN1 loss PanNET network is reported. Attractors are grouped based on their matched phenotypes. Below each attractor the basin size is reported. Activities are reported in the colored boxed. Here, green indicates active (1) and grey indicates inactive (0) genes/proteins.

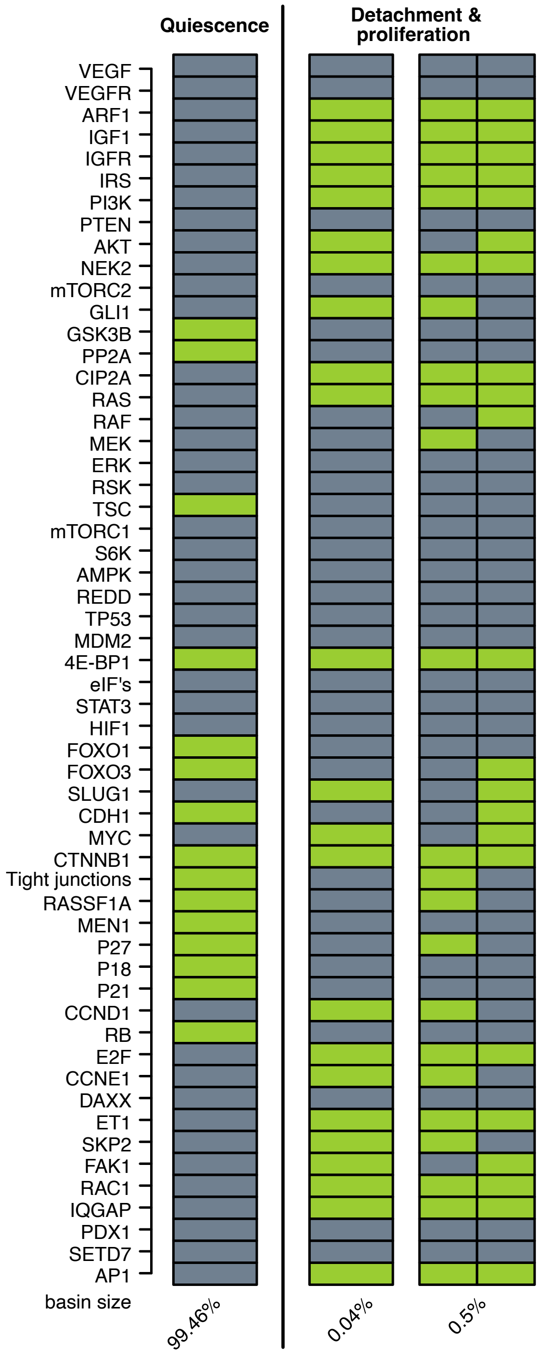

Supplementary Figure 15: mTORC1 and mTORC2 intervention attractor patterns in WT PanNETs. The attractor landscape of the mTORC1 and mTORC2 intervention on the WT PanNET network is reported. Attractors are grouped based on their matched phenotypes. Below each attractor the basin size is reported. Activities are reported in the colored boxed. Here, green indicates active (1) and grey indicates inactive (0) genes/proteins.

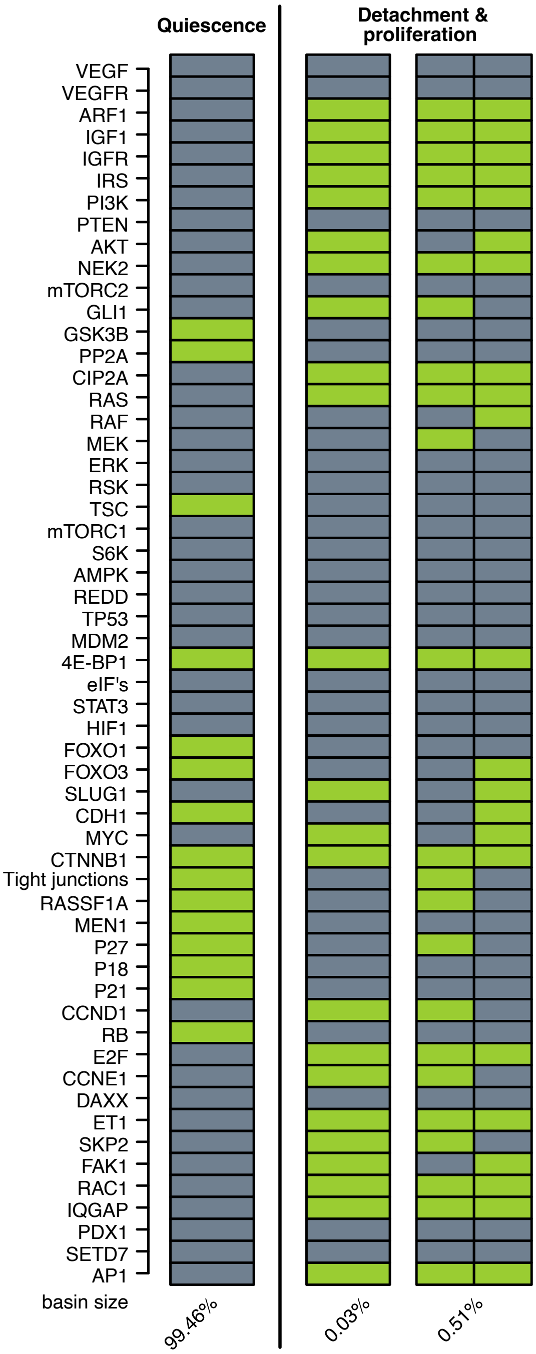

Supplementary Figure 16: mTORC1 and mTORC2 intervention attractor patterns in DAXX loss PanNETs. The attractor landscape of the mTORC1 and mTORC2 intervention on the DAXX loss PanNET network is reported. Attractors are grouped based on their matched phenotypes. Below each attractor the basin size is reported. Activities are reported in the colored boxed. Here, green indicates active (1) and grey indicates inactive (0) genes/proteins.

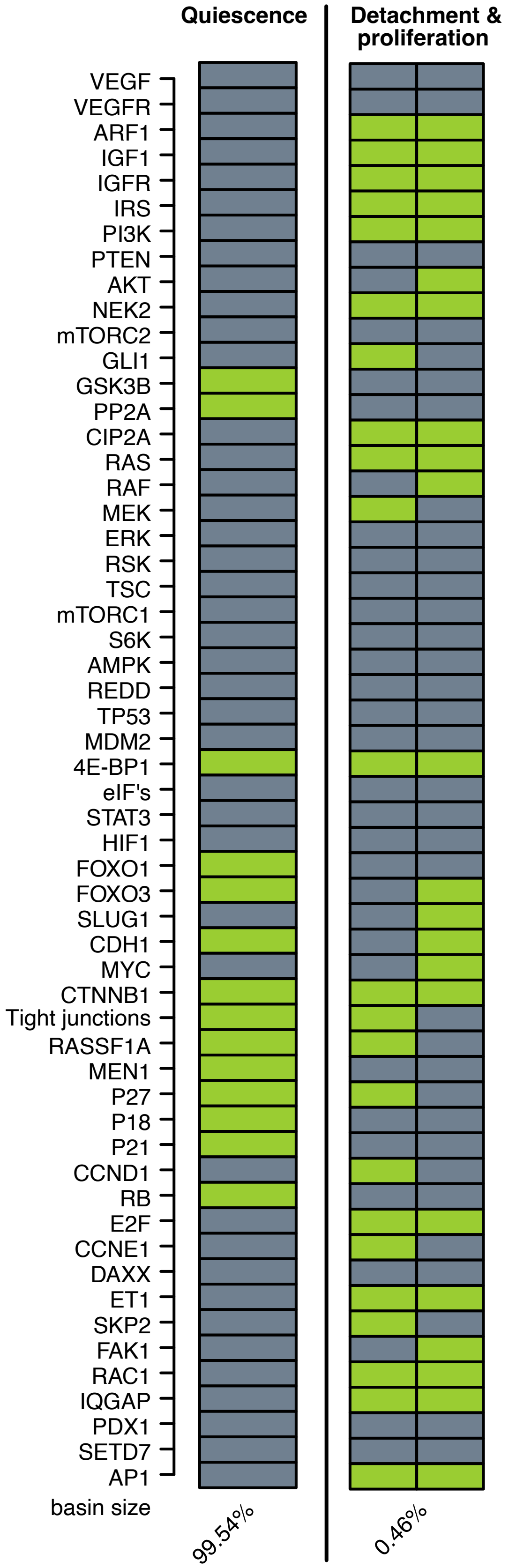

Supplementary Figure 17: mTORC1 and mTORC2 intervention attractor patterns in TSC loss PanNETs. The attractor landscape of the mTORC1 and mTORC2 intervention on the TSC loss PanNET network is reported. Attractors are grouped based on their matched phenotypes. Below each attractor the basin size is reported. Activities are reported in the colored boxed. Here, green indicates active (1) and grey indicates inactive (0) genes/proteins.

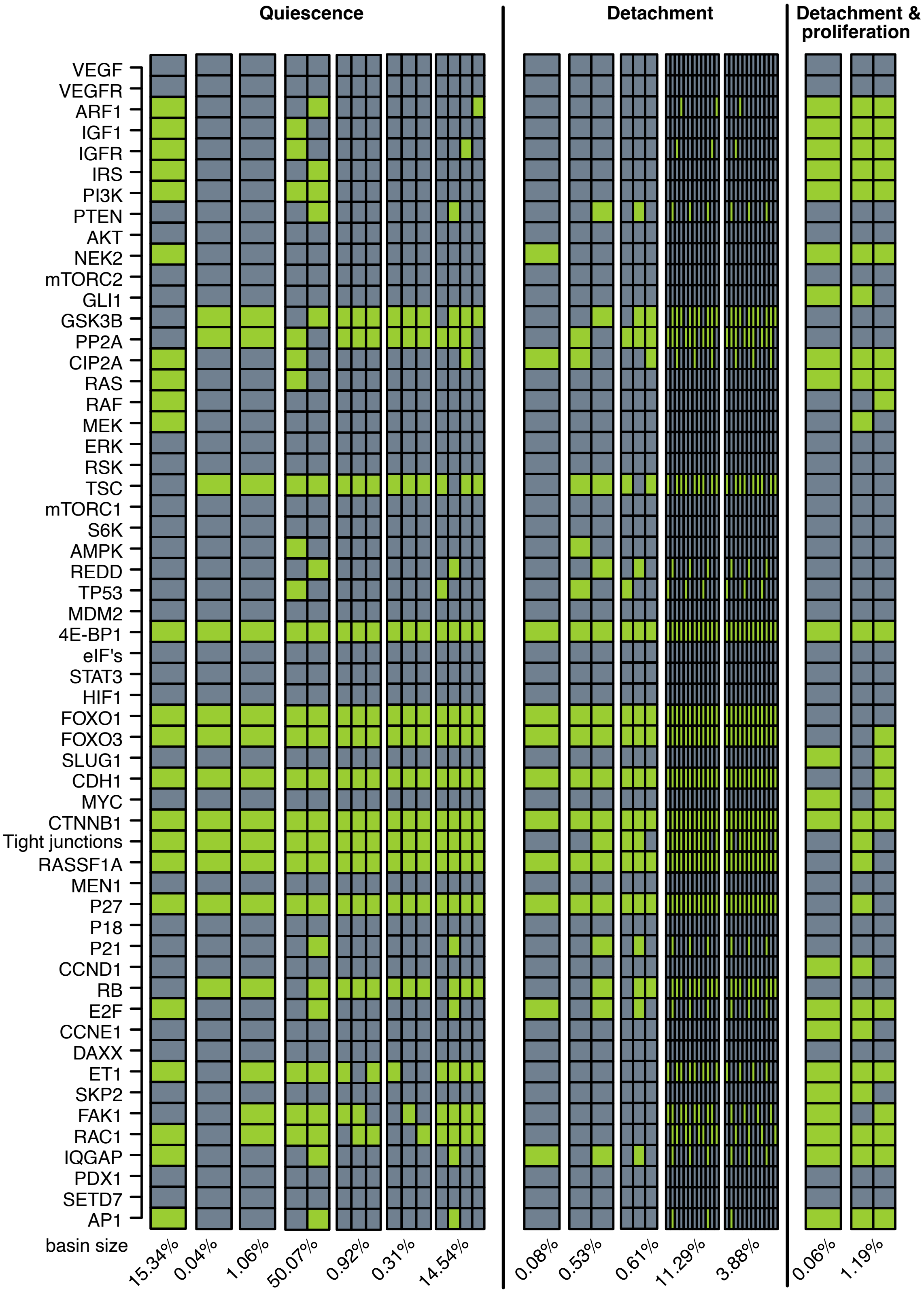

Supplementary Figure 18: mTORC1 and mTORC2 intervention attractor patterns in MEN1 loss PanNETs. The attractor landscape of the mTORC1 and mTORC2 intervention on the MEN1 loss PanNET network is reported. Attractors are grouped based on their matched phenotypes. Below each attractor the basin size is reported. Activities are reported in the colored boxed. Here, green indicates active (1) and grey indicates inactive (0) genes/proteins.

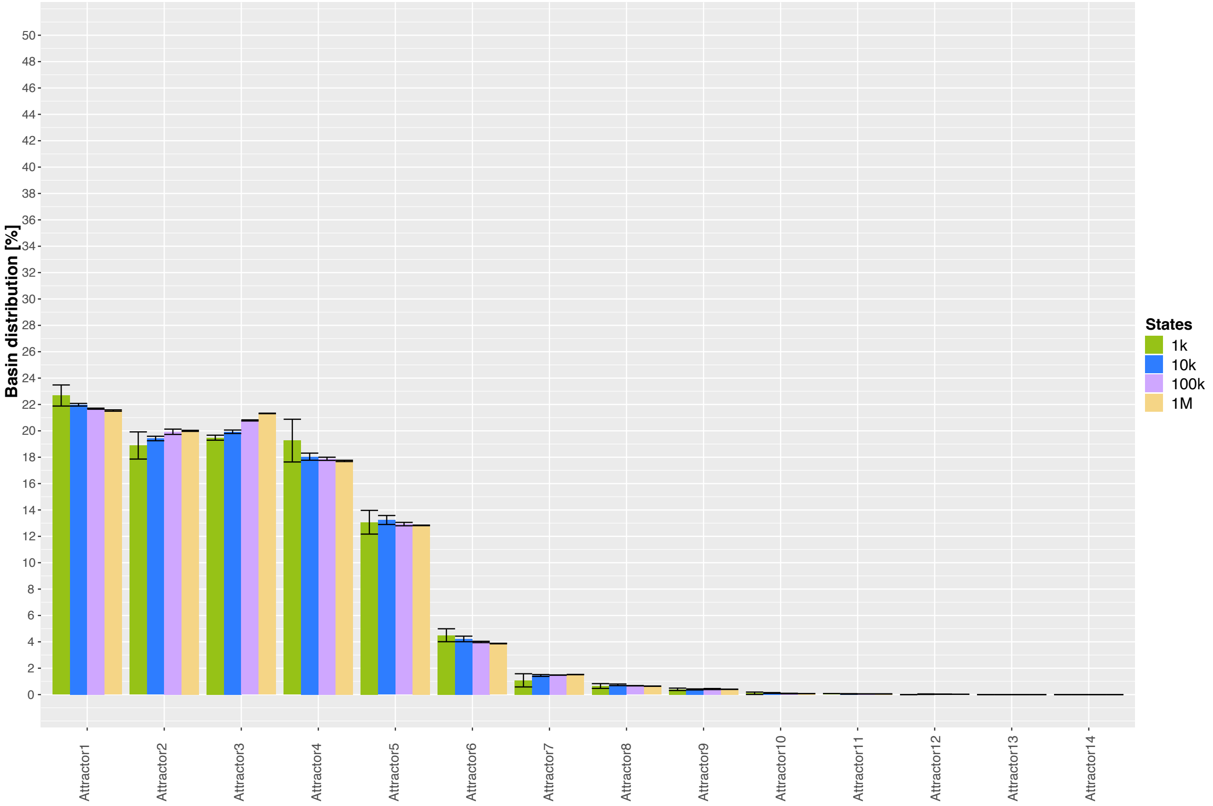

Supplementary Figure 19: Basin distribution analysis based on attractors. The basin distribution of the 14 attractors of the MEN1 loss condition is reported above. The abundance of an attractor in the basin was evaluated for 1000 (1k, in green), 10,000 (10k, in blue), 100,000 (100k, in purplr), and 1,000,000 (1M, in yellow) randomly drawn starting states. The evaluation has been performed three times for each initial condition. Standard deviations are reported in as bars in the graphs and the height of the histogram represents the means. The initial sampling of starting states does not significantly affect the basin distributions of the attractor landscape.

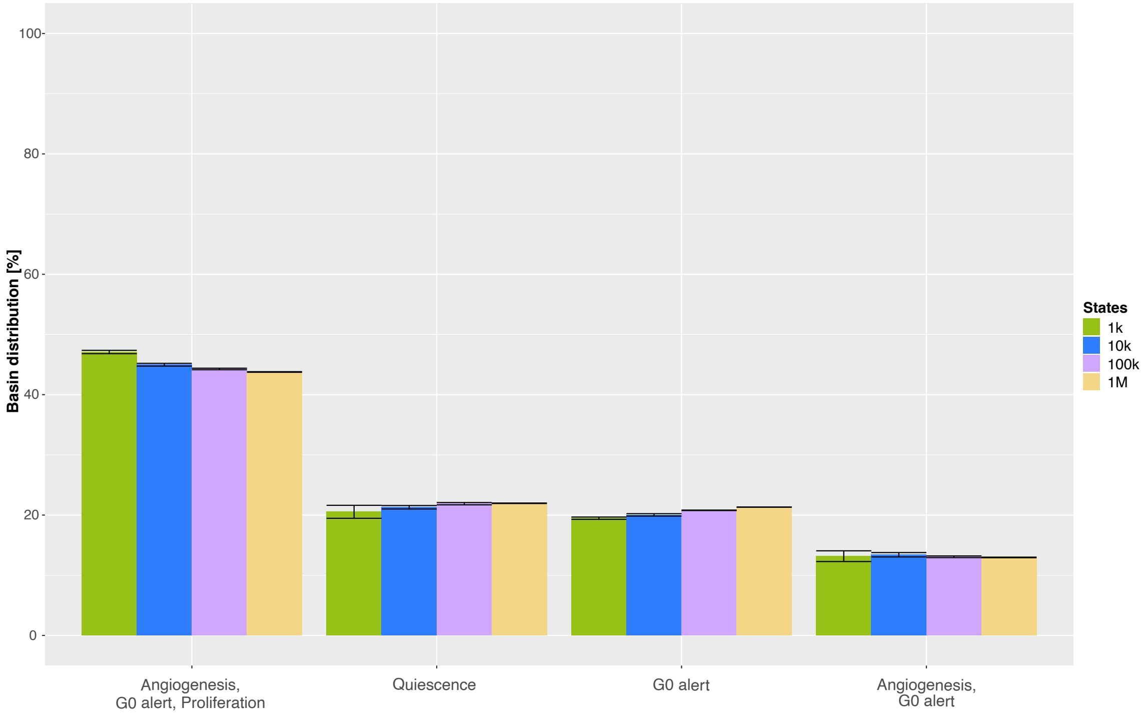

Supplementary Figure 20: Basin distribution analysis based on grouped phenotypes. The basin distribution of the four retrieved phenotypes of MEN1 loss condition is depicted above. The abundance each in the basin was evaluated for 1000 (1k, in green), 10,000 (10k, in blue), 100,000 (100k, in purplr), and 1,000,000 (1M, in yellow) randomly drawn starting states. Attractors matching the reported phenotypes were summed up in their basin abundance. The evaluation has been performed three times for each initial condition. Standard deviations are reported in as bars in the graphs and the height of the histogram represents the means. The initial sampling of starting states does not significantly affect the basin distributions of the final evaluated phenotypes.

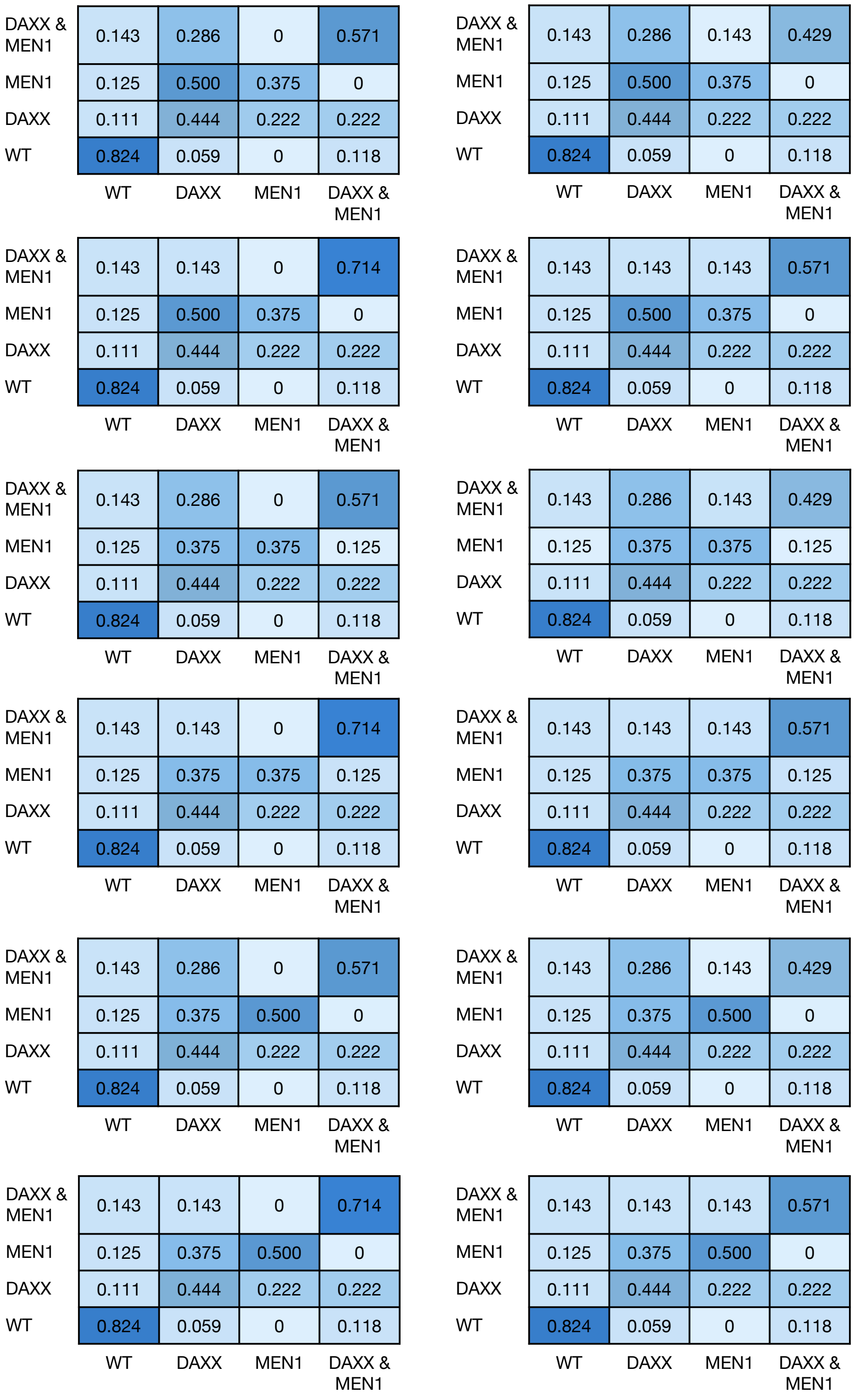

Supplementary Figure 21: Alternative equally possible solutions for the multi-class classification experiment (dataset GSE7117851). Alternative solutions depend on the presence of equally voted classes during the majority vote procedure for the experiment. The alternative matrices depicting all possibile solution of the multi-class classification experiment are depicted above. Each cell of the matrix reports the probability of a certain group to be assigned to the corresponding one in the matrix. Color intensities (here blue filled boxes) reflect these probabilities, where the higher the probability the more intense the color.

Supplementary Table 1: **Boolean functions of the PanNET model.** Regulatory interactions of the model are summarised by logic connectives AND ($\wedge$), OR ($\vee$), and NOT ($\neg$). To reduce the complexity of the model, linear interactions are depicted by time delays ($[-2]$). A detailed description of the regulatory Boolean functions is reported with references.

**Node; $t+1$**	**Boolean function; t**	**Description**
VEGF	(STAT3 $\vee$ HIF1 $\vee$ ET1) $\wedge$ eIFs	eIFs enhances cell proliferation by leading to selective tranlsation of mRNA such as VEGF [2]. VEGF promoter was activated by STAT3 [3]. STAT3 and HIF1 regulate VEGFR transcription simultanously. HIF1 upregulation leads to increased expression of angiogenic factors such as VEGF [4]. ET1 induces transcription of VEGF [5].
VEGFR	VEGF $\wedge$ ARF1 $\wedge$ ET1	VEGF is the ligand of VEGFR and responsible for receptor activation [6]. ARF1-dependent regulates cellular exit of soluble proteins such as VEGFR [7]. ET1 can upregulate the transcription of VEGFR [5].
ARF1	IGFR $\vee$ MEK $\vee$ ERK $\vee$ RAF	Activation of ARF1 is downstream of the MAPK signalling cascade. Here ARF1 activation depends on IGFR, MEK, RAF, or ERK [7, 8, 9, 10, 11].
IGF	$(E2F \vee AP1) \wedge ARF1$	AP1 is responsible of inducing IGF expression. E2F binds to the promotor of IGF and induces IGF expression [12]. ARF1 is involved in neuoren-docrine secretion and release IGF [7].
IGFR	$(\neg TP53 \wedge (AP1 \vee MYC)) \wedge (IGF \vee mTORC2 \vee FAK1)$	IGF binds to its receptor and activates it [13, 14, 15]. FAK is important for phosphorylation, and the stability of IGR1R [16]. Blocking the interaction of FAK and IGFR precludes activation of IRS [17]. mTORC2 can promote the activation of IGFR by the mTOR specific tyrosin kinase activity [18]. TP53 suppresses the IGFR promoter [19]. TP53 inhibits IGF through induction of IGF-BP3 expression that prevents binding of IGF to IGFR [13]. MYC can promote the expression of IGFR [20]. Likewise, cJUN, which is part of the AP1 transcriptional complex, is found to bind and activate the transcription of IGFR [21, 22, 23].
IRS	IGFR $\wedge$ $\neg$S6K $\wedge$ $\neg$GSK3B	S6K regulates IRS on transcriptional and translational level, thereby impairing IRS adaptor fusion [15, 4, 24]. Phosphorylation by S6K targets IRS to proteasomal degradation [4]. GSK3B phosphorylates IRS thereby reducing the insulin-stimulated activation [25]. IRS is the major substrate of IGFR [26].
PI3K	RAS $\vee$ IRS	RAS activates PI3K [27, 28]. PI3K bound to IRS converts PIP2 into PIP3 [27, 14, 15, 24].
PTEN	TP53 $\wedge$ $\neg$GSK3B	TP53 induces the expression of PTEN [13]. GSK3B phosphorylates PTEN on Thr366 or Ser370 [25]. Treatment with GSK3B inhibitors yield increase in PTEN protein levels [29].

Supplementary Table 1: **Boolean functions of the PanNET model.** Regulatory interactions of the model are summarised by logic connectives AND ($\wedge$), OR ($\vee$), and NOT ($\neg$). To reduce the complexity of the model, linear interactions are depicted by time delays ($[-2]$). A detailed description of the regulatory Boolean functions is reported with references.

**Node; $t+1$**	**Boolean function; t**	**Description**
AKT	GLI1 $\wedge$ ((PI3K $\wedge$ $\neg$PTEN) $\vee$ mTORC2 $\vee$ NEK2[-2]) $\wedge$ $\neg$PP2A $\wedge$ $\neg$MEN1	GLI1 controls AKT transcription by binding to its promotes [30]. PTEN acts as a negative regulator of PI3K/AKT signaling by reverting PIP3 into PIP2 [31, 27, 4]. PI3K activates AKT via PDK1 [13, 27, 14, 15, 4, 24, 32]. Phosphorylation of AKT by mTORC2 on Ser473 leads to full activation of AKT [13, 27, 14, 15, 4, 24, 32]. PP2A dephosphorylates AKT [33]. PP1 also dephosphorylates AKT [33]. PP1 activity toward AKT-S473 is inhibited by NEK2 [33]. This is included now as a delayed regulation on AKT from NEK2. MEN1 inhibits AKT activity by controling its subcellular localization [34].
NEK2	(GLI1 $\vee$ MYC $\vee$ E2F) $\wedge$ (CIP2A $\vee$ S6K)	GLI1 induces the transcription of NEK2 [35]. Also MYC can transcriptionally activate NEK2 [36]. Similarly, E2F is also a transcriptional regulator of NEK2 [37]. CIP2A enhances NEK2 kinase activity independent from PP2A-PP1 activity [38, 39]. S6K can phophorylate NEK2 and thereby enhancing its activity [40]
mTORC2	PI3K $\wedge$ $\neg$PTEN $\wedge$ $\neg$S6K	PIP3 phosphatase activates mTORC2. PTEN degradates PIP3 to PIP2 [13]. S6K inhibits mTORC2 [41].
GLI1	((MYC $\vee$ STAT3) $\wedge$ $\neg$MEN1 $\wedge$ $\neg$TP53) $\wedge$ ((MEK $\vee$ AKT $\vee$ ERK $\vee$ RSK) $\wedge$ ($\neg$AMPK $\wedge$ ($\neg$GSK3B $\vee$ (GSK3B $\wedge$ S6K))))	MYC binds to the promoter of GLI1 and activates its transcription [42, 43, 44, 45]. Similarly, STAT3 can activate GLI1 transcription [46, 47, 48]. MEN1 represses GLI1 expression [49]. TP53 inhibits the transcription, nuclear translocation and protein stability of GLI1 [45]. Constitutively active MEK increases GLI1 activity [45]. MEK and ERK increase the transcriptional activity of GLI1 [45]. MEK and RSK also promote GLI2 nuclear localization and stabilization [45]. AKT also increases transcriptional activity, nuclear localization and protein stability of GLI1 [45]. AMPK inhibits GLI1 via direct binding [50, 51]. S6K enhances activation of GLI by preventing its GSK3B mediated degradation [45]. GSK3B induces degradation of GLI1 [45].
GSK3B	PP2A $\wedge$ $\neg$AKT $\wedge$ $\neg$ERK	PP2A activates GSK3B [33]. AKT inactivates GSK3B by phosphorylation at Ser9 [13, 31, 4]. ERK phsophorylates GSK3B at Thr43 which primes GSK3B for subsequent phosphorylation and inactivation by RSK [24]. Furthermore, ERK phosphorylates Ser9 [52].

Supplementary Table 1: Boolean functions of the PanNET model. Regulatory interactions of the model are summarised by logic connectives AND ($\wedge$), OR ($\vee$), and NOT ($\neg$). To reduce the complexity of the model, linear interactions are depicted by time delays ($[-2]$). A detailed description of the regulatory Boolean functions is reported with references.

**Node; $t+1$**	**Boolean function; t**	**Description**
PP2A	$\neg$CIP2A	CIP2A binds to PP2A and inhibits its phosphatase functions resulting in tumorgenic transformation of cells [38, 33, 53, 54].
CIP2A	ERK[-2] $\vee$ E2F	Treatment of cells with phorbol ester TPA, a well-characterized activator of MEK-ERK signaling pathway, increased CIP2A mRNA expression more than threefold probably via ETS1, a transcription factor downstream of MEK-ERK signaling [53]. E2F induces CIP2A expression [54].
RAS	(IRS $\vee$ VEGFR) $\wedge$ $\neg$PP2A	IGF/IGFR axis also induces activation of MAPK signaling by IRS [14, 28]. VEGFR activates RAS [28]. PP2A inhibits RAS [33].
RAF1	RAS $\wedge$ $\neg$PP2A $\wedge$ ($\neg$AKT $\vee$ (AKT $\wedge\neg$NEK2[-2]))	RAS activates the RAF1-MEK1/2-ERK1/2 cascade [27, 15, 52]. RAF1 can be regulated by dephosphorylation by PP2A [4]. Inhibitory phosphorylations by AKT on RAF1 are removed by PP1 and/or PP2A during mitogen-stimulated RAF1 activation [24]. PP1 activity toward AKT-S473 is inhibited by NEK2 [33]. Here, we only included NEK2.
MEK	RAF	RAF1 activates MEK via phosphorylation [27, 52, 4].
ERK	STAT3 $\wedge$ (MEK $\vee$ RAC1[-2] $\vee$ ARF1)$\wedge$ $\neg$PP2A	STAT3 activates ERK promoter [3]. MEK activates ERK via phosphorylation [27, 15, 52, 4]. Alternatively, PAK4 a downstream target of RAC1 can also activate ERK [55]. Expression of ARF1 induces the activation of ERK [10]. PP2A inhibits ERK [33].
RSK	ERK $\wedge$ PI3K $\wedge$ $\neg$PTEN	Activated ERK phosphorylates and activates p90^{Rsk1}[15, 4, 56]. PDK1 phosphorylates RSK [56]. Full activation of RSK1 requires phosphorylation by both ERK and PI3K pathway constituent [57, 58].

Supplementary Table 1: Boolean functions of the PanNET model. Regulatory interactions of the model are summarised by logic connectives AND ($\wedge$), OR ($\vee$), and NOT ($\neg$). To reduce the complexity of the model, linear interactions are depicted by time delays ($[-2]$). A detailed description of the regulatory Boolean functions is reported with references.

**Node; $t+1$**	**Boolean function; t**	**Description**
TSC	(TP53 $\vee$ FOXO3) $\wedge$ (AMPK $\vee$ GSK3B $\vee$ REDD) $\wedge$ $\neg$AKT $\wedge$ $\neg$ERK $\wedge$ $\neg$RSK	TP53 induces the expression of TSC2 [13]. Similarly, FOXO3 can also induce the expression of TSC [59]. Activated AMPK phosphorylates and activates TSC2 [13, 15, 2, 24, 32]. GSK3B phosphorylates TSC2, thereby activating TSC2 [24]. GSK3B phosphorylates TSC2 only when TSC2 had been previously phosphorylated by AMPK [60]. Thereby GSK3B controls mTOR signaling and S6K [60]. Phosphorylation of TSC2 by AKT releases its inhibitory effect on mTOR [13, 31, 15, 4, 24, 25]. REDD1 releases TSC2 from the association with inhibitory 14-3-3 proteins, which act independently from AKT phoshorylation activity [13, 61]. REDD acts independently of the LKB2-AMPK signaling branch [15]. ERK inhibits TSC1/2 [27, 4, 32]. Activated ERK directly phosphorylates TSC2 at sites that differ from the AKT target sites [15]. RSK-mediated phosphorylation of TSC2 inhibits the TSC1-TSC2 complex [15, 32].
mTORC1	$\neg$TSC	TSC2 exerts GTPase activity to negatively regulate GTP-binding protein RHEB [13, 31, 24, 32].
S6K	mTORC1 $\wedge$ PI3K $\wedge$ $\neg$PTEN	mTORC1 mediates phosphorylation of Thr 389 within the hydrophobic motif, whereas PDK1 is responsible for phosphorylation of the T-loop [13, 33, 27, 15, 56, 26].
AMPK	(RSK[-2] $\vee$ TP53[-2]) $\wedge$ $\neg$PP2A $\wedge$ $\neg$AKT	LKB1 phosphorylates α-subunit of AMPK on Thr172 activating AMPK [13, 15, 4, 32]. LKB is phosphorylated at Ser431 by p90RSK [62]. Thus we have a linear connection from LKB to RSK to AMPK which we included as delay. Overexpression of Sestrin1 or Sestrin2 led to increases in AMPK activation [32]. TP53 induces expression of Sestrin1/2 [13]. PP2A inhibits AMPK [63, 64]. Insulin activation of AKT increases the phosphorylation of AMPK $\alpha1/\alpha2$ at Ser485/491 and inhibits AMPK signaling [65, 66, 67, 68, 69, 70] .
REDD	(HIF1 $\vee$ TP53)	HIF1 induces the expression of REDD1 and REDD2 [13, 15]. TP53 induces REDD1 expression [13].
TP53	GSK3B $\wedge$ $\neg$PP2A $\wedge$ $\neg$MDM2	Phosphorylation by GSK3B derepresses TP53 translation [25]. PP2A dephosphorylates TP53 on Ser15, thereby inactivating TP53 [13]. AKT-MDM2-TP53 forms a negative feedback loop to negatively regulate TP53 [13, 31].

Supplementary Table 1: **Boolean functions of the PanNET model.** Regulatory interactions of the model are summarised by logic connectives AND ($\wedge$), OR ($\vee$), and NOT ($\neg$). To reduce the complexity of the model, linear interactions are depicted by time delays ($[-2]$). A detailed description of the regulatory Boolean functions is reported with references.

**Node;** $t+1$	**Boolean function;** t	**Description**
MDM2	(ERK[-2] $\vee$ TP53) $\wedge$ (AKT $\vee$ (DAXX $\wedge$ $\neg$ RASSF1A))	AKT-MDM2-TP53 forms a negative feedback loop to negatively regulate TP53 [13, 31]. AKT phosphorylates MDM2 on Ser166/186 that activates MDM2 to decrease TP53 levels and activity [13, 4] in vitro. MEF, downstream of RAS/ERK can transcriptionally regulate MDM2 [71]. It has recently been shown that DAXX binds to MDM2 and is required for MDM2 activity towards TP53. Also, DAXX associates with RASSF1A and RASSF1C in vivo. Mechanistically, RASSF1A increases MDM2 self-ubiquitination by disrupting MDM2–DAXX–HAUSP interactions [72].
4EBP1	$\neg$mTORC1	mTORC1 phosphorylates 4E-BP1 [41, 24, 73].
eIFs	(RSK $\vee$ AKT $\vee$ S6K $\vee$ ERK[-2]) $\wedge$ $\neg$4EBP1	RSK, AKT, and S6K phosphorylate eIF4B on Ser422, which promotes eIF4B association with the pre-initation complex [24]. ERK activates eIFs via MNK [74]. Phosphorylation of 4EBP1 leads to the release of eIF4E, which is then free to associate with eIF4G to stimulate translation initiation [13, 15, 4, 73]. Multiple eukaryotic initiation factors (eIFs) are involved in this process. The heterotrimeric eIF4F consists of the cap-binding protein eIF4E, the scaffolding protein eIF4G, and the licase eIF4A [56].
STAT3	eIFs $\wedge$ mTORC1 $\wedge$ $\neg$SETD7 $\wedge$ $\neg$DAXX	eIF4E is a key component for translation including STAT3 [4]. mTORC1 is one of the kinases that phosphorylate STAT3 at Ser727 and rapamycin reduces STAT3 transcriptional activity [73]. STAT3 transcriptional function is inhibited by dimethylation at K140, which is catalyzed by SETD7 in response to IL-6 signaling, inhibiting STAT3 binding to DNA promoters [75, 76, 77]. DAXX supresses STAT3 transcription [78, 79].
HIF1	(STAT3 $\vee$ E2F) $\wedge$ ERK $\wedge$ eIFs $\wedge$ ($\neg$GSK3B $\vee$ (GSK3B $\wedge$ AP1))	STAT3 enhances HIF1 transcription and increases HIF1 protein level [80, 81]. E2F activity directly regulates and is required for induction of HIF1 [82]. eIF4E binds at 5'-UTRs of HIF1 and mediate its translation [83]. Phosphorylation from ERK increases HIF1 activity [84, 85, 86]. GSK3B phosphorylates HIF1 and primes it for ubiquitination. cJUN a component of the AP1 complex, can bind HIF1 at its ubiquitination sites and prevent its proteaosomal degradation [87, 88].

Supplementary Table 1: Boolean functions of the PanNET model. Regulatory interactions of the model are summarised by logic connectives AND ($\wedge$) , OR ($\vee$), and NOT ($\neg$). To reduce the complexity of the model, linear interactions are depicted by time delays ($[-2]$). A detailed description of the regulatory Boolean functions is reported with references.

**Node; $t+1$**	**Boolean function; t**	**Description**
FOXO1	$((\neg$AKT $\wedge$ $\neg$SKP2$)$ $\vee$ $($AKT $\wedge$ MEN1$)$ $\vee$ $($SKP2 $\wedge$ MEN1$))$ $\wedge$ $\neg$mTORC2	AKT phosphorylates FOXO1 [33, 89, 90] at three distinct sites, Thr24, Ser256 and Ser319. Phosphorylation of FOXO1 in the nucleus leads to export into the cytoplasm and thus repression of its transcriptional activity [91, 92, 90]. SKP2 inhibits FOXO1 through induction of its degradation [93]. MEN1 represses ubiquitination of FOXO1 protein and AKT phosphorylation. MEN1 also stabilizes FOXO1 by repressing FOXO1 degradation mediated by S-phase kinase-associated protein 2 (SKP2), an E3 ubiquitin ligase [94]. mTORC2 controls FOXO1 acetylation through inhibition of HDAC2A class [95].
FOXO3	$\neg$AKT $\wedge$ $\neg$mTORC2	AKT phosphorylates FOXO3 [33, 89]. mTORC2 controls FOXO acetylation through inhibition of HDAC2A class [95].
SLUG1	$\neg$GSK3B $\wedge$ (ERK[-2] $\vee$ GLI1) $\wedge$ ($\neg$DAXX $\vee$ (DAXX $\wedge$ HIF1))	SLUG1 bound to DAXX cannot inhibit the CDH1 promotor anymore, thus is inactive; when HIF1 is present instead, it can bind DAXX and prevent SLUG1 inhibition of CDH1 activity [96]. GSK3B controls EMT by chip-mediated degradation of SLUG1 [97]. ERK can control the expression of SLUG1, putatively via SP1 [98, 99]. GLI1 can induce transcription of SLUG1 also in BON-1 cells [100, 101, 102].
CDH1	$\neg$SLUG1 $\vee$ PDX1	SLUG1 inhibits the CDH1 promoter [103, 102]. PDX1 directly binds and activates CDH1 transcription [104].
MYC	(ERK[-2] $\vee$ (CTNNB1 $\wedge$ RAC1)) $\wedge$ $\neg$FOXO3 $\wedge$ $\neg$FOXO1 $\wedge$ $\neg$MEN1 $\wedge$ $\neg$GSK3B $\wedge$ ($\neg$PP2A $\vee$ CIP2A)	ERK controls activation of MYC via upregulation of mRNA levels [105, 106]. GSK3B enhances Thr58 phosphorylation and ubiquitination of MYC [107]. CTNNB1 presence in the nucleus, where it can induce MYC expression, controlled by RAC1 [108]. On the opposite way, MEN1 shuffles CTNNB1 from the nucleus to the cytosol [109, 110, 94]. MEN1 can also control expression of MYC [49]. FOXO1 and FOXO3 can repress MYC by inducing two miRNAs [95]. As part of the destruction complex, GSK3B inhibits the induction of MYC by CTNNB1 [111]. If not bound to cancerous inhibitor of PP2A (CIP2A), PP2A degradates MYC [112, 113].

Supplementary Table 1: Boolean functions of the PanNET model. Regulatory interactions of the model are summarised by logic connectives AND ($\wedge$), OR ($\vee$), and NOT ($\neg$). To reduce the complexity of the model, linear interactions are depicted by time delays ($[-2]$). A detailed description of the regulatory Boolean functions is reported with references.

**Node; $t+1$**	**Boolean function; t**	**Description**
CTNNB1	$\neg$GSK3B $\vee$ (GSK3B $\wedge$ $\neg$SETD7) $\vee$ (GSK3B $\wedge$ CDH1)	GSK3B phosphorylates CTNNB1 inducing its proteaosomal degradation [114, 115]. SETD7 methylates CTNNB1 at K180, which strengthens the interaction of CTNNB1 with GSK3B [75]. CTNNB1 can be sheltered from GSK3B degradation activity when bound to CDH1 at the cellular membrane. In this case, it accomplished its role in tight junctions and can not be exported in the nucleus to activate transcription of its target genes [116].
Tight Junctions (TJ)	CDH1 $\wedge$ CTNNB1 $\wedge$ ($\neg$IQGAP1 $\vee$ ((IQGAP1 $\wedge$ (RAC1 $\vee$ MEN1))))	CDH1 and CTNNB1 are essential components of tight junctions [117, 118]. The presence of CTNNB1 at the membrane is also determined by its shuffling from and to the nuclei. In this context, RAC1 promotes CTNNB1 shuffling to the nuclei, while MEN1 promotes its presence in the cytoplasm [108, 109, 110, 94]. In addition, the binding of IQGAP1 to CTNNB1 abrogates the binding of the CTNNB1/CDH1 complex to a-catenin and thus, leads to decreased adhesion [117, 118]. IQGAP1-mediated inhibition of adhesion can be relieved by binding of RAC1–GTP to IQGAP1, which displaces IQGAP1 from CTNNB1[117, 118, 119]. Also, MEN1 can cause a similar effect to RAC1 by competitively binding IQGAP1 [117].
RASSF1A	$\neg$SKP2	RASSF1A undergoes ubiquitin-mediated degradation by SKP2 [120, 72].
MEN1	FOXO1	MEN1 expression is induced by FOXO1 [91, 94, 34].
p27	(MEN1 $\vee$ FOXO3 $\vee$ PP2A) $\wedge$ $\neg$ SKP2	p27 can be induced by MEN1 [121, 122, 123, 124, 125, 126, 127]. Also FOXO3 can transcriptionally induce p27 [128, 129]. PP2A can also induce accumulation of p27 independent from the AKT pathway [130, 131]. SKP2 induces p27 degradation [132, 132].
p18	MEN1	MEN1-dependent histone methylation maintains the expression of p18 [122, 133, 123, 124, 125, 126, 127].
p21	(TP53 $\vee$ RSK[-2] $\vee$ MEN1) $\wedge$ $\neg$ SKP2 $\wedge$ $\neg$GLI1	LKB1, directly downstream of RSK, can also transcriptionally induce the expression of p21 [134, 135]. TP53 induces p21 expression [136, 72]. MEN1 can also induce p21 expression [137]. SKP2 phosphorylates p21 and prompts it for degradation [138]. p21 expression is upregulated in cells with decreased GLI1 expression [139].

Supplementary Table 1: **Boolean functions of the PanNET model.** Regulatory interactions of the model are summarised by logic connectives AND ($\wedge$), OR ($\vee$), and NOT ($\neg$). To reduce the complexity of the model, linear interactions are depicted by time delays ($[-2]$). A detailed description of the regulatory Boolean functions is reported with references.

**Node; $t+1$**	**Boolean function; t**	**Description**
CCND1	$(MYC \vee (CTNNB1 \wedge RAC1 \wedge \neg MEN1)) \wedge \neg p18 \wedge \neg RASSF1A \wedge \neg GSK3B$	MYC can induce the expression of CCND1 [140, 141, 142]. CTNNB1 presence in the nucleus, where it can induce CCND1 expression, is controlled by RAC1 [108]. On the opposite way, MEN1 shuffles CTNNB1 from the nucleus to the cytosol [109, 110, 94]. p18 inhibits CCND1 activity by inhibiting binding to CDK4 and CDK6 [143]. GSK3B, has been shown to phosphorylate CCND1 on Thr286 in vitro, and is postulated to regulate CCND1 levels and intracellular distribution [144]. RASSF1A inhibits accumulation of CCND1 [145].
RB	PP2A	PP2A dephosphorylates RB, leading to its activation [146, 147, 148, 149, 150].
E2F	$(\neg RB \vee (RB \wedge CCND1) \vee (RB \wedge PDX1)) \wedge \neg p21$	Phosphorylation of RB by CCND1 release E2F [151, 152, 153, 154]. RB can also bind and stabilize PDX1 at the same conserved site at which RB interacts with E2Fs [155]. Thus PDX1 binding to RB competes with its binding to E2Fs [155]. p21 suppresses the activity of E2F-responsive promoters (dihydrofolate reductase and CDC2) [156].
CCNE1	$E2F \wedge \neg p21 \wedge \neg p27$	E2F1 promotes CCNE1 transcription and progression from the G1 to S phase of the cell cycle [152, 153, 154]. p27 can inhibit CCNE1 by binding CDK4 [157, 158]. p21 inhibits CCNE1-CDK2 [159].
DAXX	$MEN1 \wedge ERK[-2]$	MEN1 binds and regulates DAXX activity at gene promoters [160, 91]. DAXX expression can be induced by ETS1 a transcription factor downstream of ERK [161].
ET1	$(HIF1 \wedge AP1) \vee (CTNNB1 \wedge RAC1 \wedge \neg MEN1)$	Hypoxia is one of the most potent inducers of ET1 [162, 5]. Mutations of HIF1 or AP1 in the ET1 promotor eliminate activation by hypoxia [162]. CTNNB1 can induce the expression of ET1 via binding of TCF4 to the ET1 promotor [163, 164]. CTNNB1 presence in the nucleus, where it can induce CCND1 expression, is controlled by RAC1 [108]. On the opposite way, MEN1 shuffles CTNNB1 from the nucleus to the cytosol [109, 110, 94].
SKP2	AKT	Inactivation of AKT1 decreases SKP2 mRNA levels [165].

Supplementary Table 1: **Boolean functions of the PanNET model.** Regulatory interactions of the model are summarised by logic connectives AND ($\wedge$), OR ($\vee$), and NOT ($\neg$). To reduce the complexity of the model, linear interactions are depicted by time delays ($[-2]$). A detailed description of the regulatory Boolean functions is reported with references.

**Node; $t+1$**	**Boolean function; t**	**Description**
FAK1	(ET1 $\vee$ (IGFR $\wedge$ ARF)) $\wedge$ $\neg$RAF1	ARF1 is essential for the EGF-mediated activation of FAK1 [166, 11]. ET1 signaling increases the activation of FAK1 [5, 167]. The ability of FAK1 to mediate integrin signaling in the regulation of cell cycle progression depends on the phosphorylation of Tyr397, which implies a functional significance for the formation of FAK1 signaling complexes with SRC, phosphatidylinositol-3-kinase (PI3K) and GRB7. There is a FAK1 mutant, D395A, that selectively disrupts FAK1 binding to PI3K, but allows FAK1 association with SRC. SRC-dependent association of FAK with Grb2 and p130Cas are both required for the regulation of cell cycle progression by FAK1 [168]. Overexpressed RAF1 increases phosphorylation of FAK1 at the site of Tyr407, the negative regulator of FAK1 kinase activity [169, 170]. Suppression of RAF1 in BON-1 cells induced FAK1 activation as well as increased invasion and aggressiveness, whereas MEK inhibition did not affect migration. Moreover, PanNET cells are known to have low levels of RAF1 activation [169, 170].
RAC1	(FAK1 $\vee$ PI3K $\vee$ mTORC1)	RAC1 is activated by receptor tyrosine kinases such as PI3K [171, 172]. RHO-GEF downstream of FAK1/Paxillin activates RAC [5]. FAK1 activates and translocates RAC1 for focal adhesion [173, 174]. Disruption of mTORC1 and mTORC2 by inhibiting Raptor and Rictor, respectively, inhibits the activity of RHOA and RAC1. In addition, mTORC1 was also shown to control the translation of RHOA and RAC1 mRNAs via 4E-BP1 and p70S6K [175, 176].
IQGAP1	$\neg$GSK3B	Inhibition of GSK3B frees IQGAP1 to regulate RAC1 [177].
PDX1	((RB $\wedge$ CCND1) $\vee$ RB $\vee$ ERK) $\wedge$ $\neg$GSK3B $\wedge$ $\neg$MEN1	In pancreatic cells the glucose-induced response is denpendent upon ERK1/2 phosphorylation of a subset of transcription factors, that include PDX1 [178]. RB stabilizes PDX1 and regulates its half-life [155]. Besides, PDX1 preferentially interacts with phosphorylated forms of RB, particularly via the Cdk4-dependent phosphorylation of the Ser780 residue of RB [178]. GSK3B phosphorylates PDX1 prompting it to proteosomal degradation [179, 180]. MEN1 can repress the expression of PDX1 [181].
SETD7	PDX1	SETD7 is activated at transcription level by PDX1[182].

Supplementary Table 1: **Boolean functions of the PanNET model.** Regulatory interactions of the model are summarised by logic connectives AND ($\wedge$), OR ($\vee$), and NOT ($\neg$). To reduce the complexity of the model, linear interactions are depicted by time delays ($[-2]$). A detailed description of the regulatory Boolean functions is reported with references.

**Node;** $t+1$	**Boolean function;** t	**Description**
AP1	(ERK $\vee$ (CTNNB1 $\wedge$ $\neg$GSK3B $\wedge$ $\neg$MEN1)) $\wedge$ RAC1[-2]	ERK increases the expression of AP1 transcription factor [183, 184, 185]. CTNNB1 can also transcriptionally induce AP1 [186, 187, 188]. MEN1 shuffles CTNNB1 from the nucleus to the cytosol [109, 110, 94]. GSK3B-triggered c-JUN phosphorylation inhibits AP1 [52]. JNK mediates the activation of AP1 via its phosphorylation (here RAC1[-2]) [189, 190, 191].

Supplementary Table 2: List of details on the model components.

Node	Name	Compartment	Function
VEGF	vascular endothelial growth factor	extracellular	growth factor
VEGFR	vascular endothelial grwoth factor receptor	plasma membrane	signal transduction
ARF1	ADP ribosylation factor 1	cytosol	small GTPase/ membrane trafficking
IGF1	insulin like growth factor 1	extracellular	growth factor
IGFR	insulin like growth factor 1	plasma membrane	signal transduction
IRS	insulin receptor substrate	cytosol	signal transduction
PI3K	phosphatidylinositol 3-kinase	cytosol	kinase
PTEN	phosphtase and tensin homolog	cytosol	phosphatase
AKT	AKT serine/threonine kinase	cytosol	kinase
NEK2	NIMA related kinase 2	cytosol	kinase
mTORC2	mechanistic target of rapamycin kinase complex 2	cytosol	kinase
GLI1	GLI family zinc finger 1	nucleus	ttranscription factor
GSK3B	glycogen synthase kinase 3 beta	cytosol	kinase
PP2A	protein phopsphatase 2A	cytosol	phosphatase
CIP2A	cellular inhibitor of PP2A	cytosol	phosphatase inhibitor (binding)
RAS	rat sarcoma	cytosol	GTPase/ signal transduction
RAF	raf proto-oncogene	cytosol	kinase
MEK	mitogen activated protein kinase kinase	cytosol	kinase
ERK	mitogen activated protein kinase	cytosol	kinase
RSK	ribosomal protein S6 kinase	cytosol	kinase
TSC	TSC complex	cytosol	signal transduction
mTORC1	mechanistic target of rapamycin kinase complex 1	cytosol	kinase
S6K	ribosomal protein S6 kinase B1	cytosol	kinase
AMPK	protein kinase AMP-activated	nucleus	kinase
REDD	DNA damage inducible transcript	cytosol	signal transduction
TP53	tumor protein p53	nucleus & cytosol	transcription factor
MDM2	MDM2 proto-oncogene	nucleus & cytosol	E3 ubiquitin-protein ligase
4E-BP1	eukaryotic translation factor 4E binding protein 1	cytosol	repressor of translation initiation
eIF's	eukaryotic translation initiation factor 4E	cytosol	activator of protein synthesis
STAT3	signal transducer and activator of transcription 3	cytosol & nucleus	signal transduction & transcription factor
HIF1	hypoxia inducible factor 1	cytosol & nucleus	transcription factor
FOXO1	forkhead box O1	cytosol & nucleus	transcription factor
FOXO3	forkhead box O3	cytosol & nucleus	transcription factor
SLUG1	snail family transcription repressor 2	nucleus	transcription factor
CDH1	E-cadherin	plasma membrane	cell adhesion
MYC	MYC proto-oncogene	nucleus	transcription factor
RASSF1A	Ras association domain family member 1	nucleus & cytosol	signal transduction
MEN1	menin 1	cytosol & nucleus	transcriptional factor
p27	cyclin dependent kinase inhibitor 1B	nucleus & cytosol	CDK inhibitor
p18	cyclin dependent kinase inhibitor 2C	nucleus & cytosol	CDK inhibitor
p21	cyclin dependent kinase inhibitor 1A	nucleus & cytosol	CDK inhibitor
CCND1	cyclin D1	nucleus	kinase complex
RB	RB transcriptional corepressor	nucleus	signal transduction
E2F	E2F transcription factor	nucleus	transcription factor
CCNE1	cyclin E1	nucleus	kinase complex
DAXX	death domain associated protein	cytosol & nucleus	transcription factor
ET1	endothelin 1	cytosol	membrane trafficking

SKP2	S-phase kinase associated protein 3	cytosol & nucleus	E3 ubiquitin-protein ligase
FAK1	protein tyrosine ligase	cytosol	kinase
RAC1	rac family small GTPase 1	cytosol	cell adhesion
IQGAP	IQ motif containing GTPase activating protein 1	cytosol & plasma membrane	cell adhesion
PDX1	pancreatic and duodenal homeobox 1	nucleus	transcription factor
SETD7	SET domain containing 7	nucleus	histone methyltransferase
AP1	AP1 transcription factor	nucleus	transcription factor

Supplementary Table 3: Comparison of mutated attractor landscapes with knowledge on PanNETs. Each simulated mutated condition (WT, DAXX loss, TSC loss, and MEN1 loss) is summmarized in terms of attractor matched phenotypes. Matched information from PanNETs mouse models and patient cohorts is reported and matched, when available. Note that, due to the rarity of PanNETs, patients cohorts for specific mutations collect a rather limited number of patients.

**Muation**	**Attractor mathched phenotypes**	**Mouse model information**	**Patient cohort information**
WT	99% quiescent, 1% angiogenesis, proliferation, detachment	–	WT PanNETs showed better prognosis compared mutated ones [192].
DAXX	98.9% quiescent, 1.1% angiogenesis, proliferation, detachment 0.1% G0 alert, detachment	Conditional Pdx1-cre driven DAXX inactivation could indicate that DAXX alone has limited effect on pancreatic development and function. However, an alteration of transcriptome was observed, creative permissive chromatin states favoring further mutations and progression [193]. Another mouse model for DAXX inactivation showed comparable results on the tollerability of DAXX loss in the pancreas [194]	Patients in showing DAXX loss mutations show contrasting results, [195] with certain cohorts associating DAXX loss to positive prognosis [196, 197, 198, 199], and others to negative prognosis [200, 201, 202, 198]. However, in accordance to the mouse models results, DAXX loss has been associated to chromosomal instability, specifically altered telomer lengthnening, in patient cohorts [196, 201, 202, 198]. Differences observed in DAXX patients cohorts have been hypothesized to be imputed to facturs such of the metastatic status of the patients, or the evaluation of histopathological results [195].
TSC	99% G0 alert, 1% angiogenesis, proliferation, detachment	–	Genetic loss of TSC in most of the cases does not correlate with aggressive PanNETs [203].
MEN1	22% quiescence, 20% G0 alert, 13% angiogenesis, G0 alert, 44% angiogenesis, proliferation, detachment	Mouse models with heterogeneous loss of MEN1 develop multiple endocrine tumors, but they do not develop up to carcinoma stages [204].	MEN1 is the most frequently mutated gene in human PanNETs, associated with the presence of multiple neuroendocrine tumors [196].

Supplementary Table 4: Model validation. The activity levels of model compounds compared to literature descriptions or gene expression datasets. This table is the reference for Figure 5 of the main.

Pathway	Node	Model-based activity	Validation
Angiogenesis	VEGF	active	[205, 206]
Angiogenesis	VEGFR	active	[205, 206]
Angiogenesis & cell cycle	HIF1	partially active	[205]
Angiogenesis & MAPK signaling	ARF1	active	[207]
MAPK signaling	FAK1	active	[208]
MAPK signaling	RAF	constant	[209, 210]
MAPK signaling	ERK	partially active	[211]
MAPK signaling	NEK2	partially active	[212, 213]
MAPK signaling & PI3K/AKT signaling	GLI1	active	[102]
MAPK signaling & PI3K/AKT signaling	IGF	active	[214]
MAPK signaling & PI3K/AKT signaling	IGFR	active	[215, 216]
MAPK signaling & PI3K/AKT signaling	PP2A	inactive	[33]
MAPK signaling & PI3K/AKT signaling	CIP2A	active	[214]
PI3K/AKT signaling	PI3K	active	[217]
PI3K/AKT signaling	AKT	active	[211, 208, 218, 219]
PI3K/AKT signaling	PTEN	constantly inactive	[220, 221]
PI3K/AKT signaling	GSK3B	inactive	[222, 223]
PI3K/AKT signaling	FOXO1	inactive	[224]
PI3K/AKT signaling	FOXO3	inactive	[225]
PI3K/AKT signaling & cell cycle	RASSF1A	inactive	[226, 227]
Cell cycle	MDM2	partially active	[228, 229]
Cell cycle	TP53	constant	[228, 229]
Cell cycle	MYC	active	[230, 231]
Cell cycle	MEN1	inactive	[232, 233]
Cell cycle	p27	inactive	[234, 235]
Cell cycle	p21	inactive	[236]
Cell cycle	p18	inactive	[234]
Cell cycle	CCND1	active	[211, 237]
Cell cycle	RB	inactive	[238]
Cell cycle	E2F	active	[214]
Cell cycle	CCNE1	active	[214]
Cell cycle	DAXX	inactive	[239]
mTORC signaling	mTORC1	active	[240]
mTORC signaling	TSC	inactive	[220]
mTORC signaling	mTORC2	constant	[240]
mTORC signaling	STAT3	partially active	[241]
Cell adhesion	CDH1	partially active	[214]
Cell adhesion & cell cycle	CTNNB1	active	[242]
Cell adhesion & cell cycle	SLUG	active	[221, 243]
Cell adhesion & cell cycle	PDX1	partially inactive	[244, 245]

Supplementary References

[1] Csardi, G. & Nepusz, T. The igraph software package for complex network research. InterJournal **Complex Systems**, 1695 (2006). URL https://igraph.org.

[2] Meric-Bernstam, F. & Gonzalez-Angulo, A. M. Targeting the mTOR signaling network for cancer therapy. Journal of Clinical Oncology **27**, 2278–2287 (2009).

[3] Kan, J. et al. S-propargyl-cystein, a novel watter-soluble modulator of endogenous hydogen sulfide, promotes angiogenesis through activation of singal transducer and activator of transcription 3. Antioxidants & Redox Signaling **20**, 2303–2316 (2014).

[4] McCubrey, J. A. et al. Mutations and deregulation of RAS/RAF/MEK/ERK and PI3K/PTEN/AKT/mTOR cascades which alter therapy resonse. Oncotraget **3**, 954–987 (2012).

[5] Rosanò, L., Spinella, F. & Bagnato, A. Endothelin 1 in cancer: biological implications and therapeutic opportunities. Nature Reviews Cancer **13**, 637–651 (2013).

[6] Bösch, F. et al. Distinct expression patterns of VEGFR 1-3 in gastroenteropancreatic neuroendocrine neoplasms: Supporting clinical relevance, but not a prognostic factor. Journal of clinical medicine **9**, 3368 (2020).

[7] Münzberg, C. et al. IGF-1 drives chromogranin A secretion via activation of Arf1 in human neuroendocrine tumour cells. Journal of Cellular and Molecular Medicine **19**, 948–959 (2015).

[8] Shome, K., Vasudevan, C. & Romero, G. Arf proteins mediate insulin-dependent activation of phospholipase D. Current Biology **7**, 387–396 (1997).

[9] Haines, E., Saucier, C. & Claing, A. The adaptor proteins p66shc and grb2 regulate the activation of the gtpases ARF1 and ARF6 in invasive breast cancer cells. Journal of Biological Chemistry **289**, 5687–5703 (2014).

[10] Davis, J. E. et al. Arf1 promotes prostate tumorigenesis via targeting oncogenic MAPK signaling. Oncotarget **7**, 39834 (2016).

[11] Casalou, C., Ferreira, A. & Barral, D. C. The role of ARF family proteins and their regulators and effectors in cancer progression: a therapeutic perspective. Frontiers in Cell and Developmental Biology **8** (2020).

[12] Yu, J. T., Foster, R. G. & Dean, D. C. Transcriptional repression by Rb-E2F and regulation of anchorage-independent survival. Molecular and Cellular Biology **21**, 3325–3335 (2001).

[13] Feng, Z. & Levine, A. J. The regulation of energy metabolism and the IGF-1/mTOR pathways by the p53 protein. Trends in Cell Biology **20**, 427 – 434 (2010).

[14] Djukom, C. et al. Dual inhibition of PI3K and mTOR signaling pathways decreases human pancreatic neuroendocrine tumor PNET metastatic progression. Pancreas **43**, 88–92 (2010).

[15] Wullschleger, S., Loewith, R. & Hall, M. N. TOR signaling in growth and metabolism. Cell **124**, 471–484 (2006).

[16] Andersson, S., D'Arcy, P., Larsson, O. & Sehat, B. Focal adhesion kinase (FAK) activates and stabilizes IGF-1 receptor. Biochemical and Biophysical Research Communications **387**, 36–41 (2009).

[17] Ucar, D. A. et al. Disruption of the protein interaction between FAK and IGF-1R inhibits melanoma tumor growth. Cell Cycle **11**, 3250–3259 (2012).

[18] Yin, Y. et al. mtorc2 promotes type i insulin-like growth factor receptor and insulin receptor activation through the tyrosine kinase activity of mtor. Cell research **26**, 46–65 (2016).

[19] Werner, H., Karnieli, E., Rauscher, F. J. & LeRoith, D. Wild-type and mutant p53 differentially regulate transcription of the insulin-like growth factor I receptor gene. Proceedings of the National Academy of Sciences of the United States of America **93**, 8318–8323 (1996).

[20] Sun, J. et al. Up-regulation of insr/igf1r by c-myc promotes tscc tumorigenesis and metastasis through the nf-κb pathway. Biochimica et Biophysica Acta (BBA)-Molecular Basis of Disease **1864**, 1873–1882 (2018).

[21] Scheidegger, K. J., Du, J. & Delafontaine, P. Distinct and common pathways in the regulation of insulin-like growth factor-1 receptor gene expression by angiotensin ii and basic fibroblast growth factor. Journal of Biological Chemistry **274**, 3522–3530 (1999).

[22] Sarfstein, R., Belfiore, A. & Werner, H. Identification of insulin-like growth factor-i receptor (igf-ir) gene promoter-binding proteins in estrogen receptor (er)-positive and er-depleted breast cancer cells. Cancers **2**, 233–261 (2010).

[23] Rizzolio, S. et al. Neuropilin-1 upregulation elicits adaptive resistance to oncogene-targeted therapies. The Journal of clinical investigation **128**, 3976–3990 (2018).

[24] Mendoza, M. C., Er, E. E. & Blenis, J. The Ras-ERK and PI3K-mTOR pathways: cross-talk and compensation. Trends in Biochemical Sciences **36**, 320–328 (2011).

[25] Hermida, M. A., Kumar, J. D. & Leslie, N. R. GSK3 and its interactions with the PI3K/AKT/mTOR signalling network. Advances in Biological Regulation **65**, 5–15 (2017).

[26] von Wichert, G. et al. Insulin-like growth factor-i is an autocrine regulaotr of Chromogranin A secretion and growth in human neuroendocrine tumor cells. Cancer Research **60**, 4573–4581 (2000).

[27] Vandamme, T. et al. Long-term acquired everolimus resistance in pancreatic neuroendocrine tumours can be overcome with novel PI3K-AKT-mTOR inhibitors. Britisch Journal of Cancer **114**, 650–658 (2016).

[28] Yim, K.-L. Everolimus and mTOR inhibition in pancreatic neuroendocrine tumors. Cancer Management and Research **4**, 2017–214 (2012).

[29] Maccario, H., Perera, N. M., Davidson, L., Downes, C. P. & Leslie, N. R. PTEN is destabilized by phosphorylation on thr^{366}. Biochemical Journal **405**, 439–444 (2007).

[30] Agarwal, N. K., Qu, C., Kunkulla, K., Liu, Y. & Vega, F. Transcriptional regulation of serine/threonine protein kinase (akt) genes by glioma-associated oncogene homolog 1. Journal of Biological Chemistry **288**, 15390–15401 (2013).

[31] Porta, C., Paglino, C. & Mosca, A. Targeting PI3K/Akt/mTOR signaling in cancer. Frontiers in Oncology **4** (2014).

[32] Shaw, R. J. LKB1 and AMP-activated protein kinase control of mTOR signalling and growth. Acta Physiologica **196**, 65–80 (2009).

[33] Umesalma, S. et al. RABL6A inhibits tumor-suppressive PP2A/AKT signaling to drive pancreatic neuroendocrine tumor growth. The Journal of Clinical Investigation **129**, 1641–1653 (2019).

[34] Wang, Y. et al. The tumor suppressor protein menin inhibits akt activation by regulating its cellular localization. Cancer research **71**, 371–382 (2011).

[35] Zhou, F. et al. Nek2A/SuFu feedback loop regulates Gli-mediated Hedgehog signaling pathway. International Journal of Oncology **50**, 373–380 (2017).

[36] Gu, Z. et al. Nek2 promotes aerobic glycolysis in multiple myeloma through regulating splicing of pyruvate kinase. Journal of hematology & oncology **10**, 1–11 (2017).

[37] Lee, M.-Y., Moreno, C. S. & Saavedra, H. I. E2f activators signal and maintain centrosome amplification in breast cancer cells. Molecular and cellular biology **34**, 2581–2599 (2014).

[38] De, P., Carlson, J., Leyland-Jones, B. & Dey, N. Oncongenic nexus of cancerous inhibitor of protein phosphatase 2A (CIP2A): an oncoprotein with many hands. Oncotarget **5**, 4581–4602 (2014).

[39] Jeong, A. L. et al. Cancerous inhibitor of protein phosphatase 2a (cip2a) protein is involved in centrosome separation through the regulation of nima (never in mitosis gene a)-related kinase 2 (nek2) protein activity. Journal of Biological Chemistry **289**, 28–40 (2014).

[40] Di Agostino, S., Rossi, P., Geremia, R. & Sette, C. The mapk pathway triggers activation of nek2 during chromosome condensation in mouse spermatocytes. Development **129**, 1715–1727 (2002).

[41] Xao, J. C., Phan, A. T., Jehl, V., Shah, G. & Meric-Bernstam, F. Everolimus in advanced pancreatic neuroendocrine tumors: The clinical experience. Cancer Research **73**, 1449–1453 (2013).

[42] Liu, X. et al. Yamanaka factors critically regulate the developmental signaling network in mouse embryonic stem cells. Cell research **18**, 1177–1189 (2008).

[43] Varnat, F., Siegl-Cachedenier, I., Malerba, M., Gervaz, P. & Ruiz i Altaba, A. Loss of wnt-tcf addiction and enhancement of hh-gli1 signalling define the metastatic transition of human colon carcinomas. EMBO molecular medicine **2**, 440–457 (2010).

[44] Yoon, J. W. et al. Noncanonical regulation of the hedgehog mediator gli1 by c-myc in burkitt lymphoma. Molecular Cancer Research **11**, 604–615 (2013).

[45] Pietrobono, S., Gagliardi, S. & Stecca, B. Non-canonical hedgehog signaling pathway in cancer: Activation of GLI transcription factors beyond smoothened. Frontiers in Genetics **10**, 556 (2019).

[46] Sirkisoon, S. R. et al. Interaction between stat3 and gli1/tgli1 oncogenic transcription factors promotes the aggressiveness of triple-negative breast cancers and her2-enriched breast cancer. Oncogene **37**, 2502–2514 (2018).

[47] Carr, R. M. et al. The extracellular sulfatase sulf2 promotes liver tumorigenesis by stimulating assembly of a promoter-looping gli1-stat3 transcriptional complex. Journal of Biological Chemistry **295**, 2698–2712 (2020).

[48] Brennan-Crispi, D. M. et al. Overexpression of desmoglein 2 in a mouse model of gorlin syndrome enhances spontaneous basal cell carcinoma formation through stat3-mediated gli1 expression. Journal of Investigative Dermatology **139**, 300–307 (2019).

[49] Gurung, B., Feng, Z. & Hua, X. Menin directly represses Gli1 expression independent of canonical Hedgehog signaling. Molecular Cancer Research **11**, 1215–1222 (2013).

[50] Xu, Q. et al. The transcriptional activity of gli1 is negatively regulated by ampk through hedgehog partial agonism in hepatocellular carcinoma. International journal of molecular medicine **34**, 733–741 (2014).

[51] Zhang, R. et al. Dual degradation signals destruct GLI1: AMPK inhibits GLI1 through β-TrCP-mediated proteasome degradation. Oncotarget **8**, 49869–49881 (2017).

[52] Zeller, E., Hammer, K., Kirschnick, M. & Braeuning, A. Mechanisms of RAS/β-catenin interactions. Archives of Toxikology **87**, 611–632 (2013).

[53] Khanna, A. et al. ETS1 mediates MEK1/2-dpendent overexpression of cancerous inhibitor of protein phosphatase 2A (CIP2A) in human cancer cells. PLOS ONE **6**, e17979 (2011).

[54] Soofiyani, S. R., Hejazi, M. S. & Baradaran, B. The role of CIP2A in cancer: A review and update. Biomedicine & Pharmacotherapy **96**, 626–633 (2017).

[55] Mpilla, G. et al. Pak4-nampt dual inhibition as a novel strategy for therapy resistant pancreatic neuroendocrine tumors. Cancers **11**, 1902 (2019).

[56] Shahbazian, D. et al. The mTOR/PI3K and MAPK pahtways converge on eIF4B to control its phosphorylation and activity. The EMBO Journal **25**, 2781–2791 (2006).

[57] Romeo, Y., Zhang, X. & Roux, P. P. Regulation and function of the RSK family of protein kinases. Biochemical Journal **441**, 553–569 (2012).

[58] Creson, T. K., Yuan, P., Manji, H. K. & Chen, G. Evidence for involvement of ERK, PI3K, and RSK in induction of Bcl-2 by valproate. Journal of Molecular Neuroscience **37**, 123–134 (2009).

[59] Khatri, S., Yepiskoposyan, H., Gallo, C. A., Tandon, P. & Plas, D. R. FOXO3a regulates glycolysis via transcriptional control of tumor suppressor TSC1. Journal of Biological Chemistry **285**, 15960–15965 (2010).

[60] Inoki, K. et al. TSC2 integrates wnt and energy signals via a coordinated phosphorylation by AMPK and GSK3 to regulate cell growth. Cell **126**, 955–968 (2006).

[61] Shumway, S. D., Li, Y. & Xiong, Y. 14-3-3β binds to and negatively regulates the tuberous sclerosis complex 2 (tsc2) tumor suppressor gene product, tuberin. Journal of Biological Chemistry **278**, 2089–2092 (2003).

[62] Sapkota, G. P. et al. Phosphorylation of the protein kinase mutated in Peutz-Jegehrs cancer syndrome, LKB1/STK11, at Ser^{431} by p90^{RSK} and cAMP-dependent protein kinase, but not its farnesylation at Cys^{4}33, is essential for LKB1 to suppress cell growth. The Journal of Biological Chemistry **276**, 19469–19482 (2001).

[63] Khanal, P., Kim, G., Yun, H. J., Cho, H.-G. & Choi, H. S. The prolyl isomerase Pin1 interacts with and downregulates the activity of AMPK leading to induction of tumorigenicity of hepatocarcinoma cells. Molecular Carcinogenesis **52**, 813–823 (2013).

[64] Gao, X. et al. γ-6-phosphogluconolactone, a byproduct of the oxidative pentose phosphate pathway, contributes to ampk activation through inhibition of pp2a. Molecular cell **76**, 857–871 (2019).

[65] Horman, S. et al. Insulin antagonizes ischemia-induced thr172 phosphorylation of amp-activated protein kinase α-subunits in heart via hierarchical phosphorylation of ser485/491. Journal of Biological Chemistry **281**, 5335–5340 (2006).

[66] Soltys, C.-L. M., Kovacic, S. & Dyck, J. R. Activation of cardiac amp-activated protein kinase by lkb1 expression or chemical hypoxia is blunted by increased akt activity. American Journal of Physiology-Heart and Circulatory Physiology **290**, H2472–H2479 (2006).

[67] Beauloye, C. et al. Insulin antagonizes amp-activated protein kinase activation by ischemia or anoxia in rat hearts, without affecting total adenine nucleotides. FEBS letters **505**, 348–352 (2001).

[68] Kovacic, S. et al. Akt activity negatively regulates phosphorylation of amp-activated protein kinase in the heart. Journal of Biological Chemistry **278**, 39422–39427 (2003).

[69] Saha, M. et al. Ampk–akt double-negative feedback loop in breast cancer cells regulates their adaptation to matrix deprivation. Cancer research **78**, 1497–1510 (2018).

[70] Hahn-Windgassen, A. et al. Akt activates the mammalian target of rapamycin by regulating cellular atp level and ampk activity. Journal of Biological Chemistry **280**, 32081–32089 (2005).

[71] Sashida, G. et al. ELF4/MEF activates MDM2 expression and blocks oncogene-induced p16 activation to promote transformation. Molecular and cellular biology **29**, 3687–3699 (2009).

[72] Song, M. S., Song, S. J., Kim, S. Y., Oh, H. J. & Lim, D.-S. The tumour suppressor RASSF1A promotes MDM2 self-ubiquitination by disrupting the MDM2–DAXX–HAUSP complex. The EMBO journal **27**, 1863–1874 (2008).

[73] Laplante, M. & Sabatini, D. M. Regulation of mTORC1 and its impact on gene expression at a glance. Journal of Cell Science **126**, 1713–1719 (2013).

[74] Roux, P. P. & Topisirovic, I. Regulation of mrna translation by signaling pathways. Cold Spring Harbor perspectives in biology **4**, a012252 (2012).

[75] Batista, I. d. A. A. & Helguero, L. A. Biological processes and signal transduction pathways regulated by the protein methyltransferase SETD7 and their significance in cancer. Signal transduction and targeted therapy **3**, 1–14 (2018).

[76] Stark, G. R., Wang, Y. & Lu, T. Lysine methylation of promoter-bound transcription factors and relevance to cancer. Cell research **21**, 375–380 (2011).

[77] Koo, M. Y. et al. Selective inhibition of the function of tyrosine-phosphorylated stat3 with a phosphorylation site-specific intrabody. Proceedings of the National Academy of Sciences **111**, 6269–6274 (2014).

[78] Muromoto, R. et al. Physical and functional interactions between Daxx and STAT3. Oncogene **25**, 2131–2136 (2006).

[79] Matsuda, T. et al. Signal transducer and activator of transcription 3 regulation by novel binding partners. World journal of biological chemistry **6**, 324 (2015).

[80] Demaria, M. et al. A sTAT3-mediated metabolic switch is involved in tumour transformation and STAT3 addiction. Aging **2**, 823–842 (2010).

[81] Demaria, M. & Poli, V. PKM2, STAT3 and HIF-1α. JAK-STAT **1**, 194–196 (2012).

[82] Sengupta, T., Abraham, G., Xu, Y., Clurman, B. E. & Minella, A. C. Hypoxia-inducible factor 1 is activated by dysregulated cyclin e during mammary epithelial morphogenesis. Molecular and cellular biology **31**, 3885–3895 (2011).

[83] Lim, J.-H. et al. Prmt5 is essential for the eif4e-mediated 5-cap dependent translation. Biochemical and biophysical research communications **452**, 1016–1021 (2014).

[84] Huang, Z. & Bao, S.-D. Roles of main pro-and anti-angiogenic factors in tumor angiogenesis. World journal of gastroenterology **10**, 463 (2004).

[85] Ramos, J. W. The regulation of extracellular signal-regulated kinase (erk) in mammalian cells. The international journal of biochemistry & cell biology **40**, 2707–2719 (2008).

[86] Hong, K. H., Ryu, J. & Han, K. H. Monocyte chemoattractant protein-1–induced angiogenesis is mediated by vascular endothelial growth factor-a. Blood **105**, 1405–1407 (2005).

[87] Yu, B. et al. c-jun protects hypoxia-inducible factor-1α from degradation via its oxygen-dependent degradation domain in a nontranscriptional manner. Cancer research **69**, 7704–7712 (2009).

[88] Flügel, D., Görlach, A., Michiels, C. & Kietzmann, T. Glycogen synthase kinase 3 phosphorylates hypoxia-inducible factor 1α and mediates its destabilization in a vhl-independent manner. Molecular and cellular biology **27**, 3253–3265 (2007).

[89] Hay, N. Interplay between FOXO, TOR, and Akt. Biochimica et Biophysica Acta (BBA)-Molecular Cell Research **1813**, 1965–1970 (2011).

[90] Martinez, S. C., Cras-Méneur, C., Bernal-Mizrachi, E. & Permutt, M. A. Glucose regulates foxo1 through insulin receptor signaling in the pancreatic islet β-cell. Diabetes **55**, 1581–1591 (2006).

[91] Feng, Z., Ma, J. & Hua, X. Epigenetic regulation by the menin pathway. Endocrine-Related Cancer **24**, T147–T159 (2017).

[92] Vogt, P. K., Jiang, H. & Aoki, M. Triple layer control: phosphorylation, acetylation and ubiquitination of foxo proteins. Cell cycle **4**, 908–913 (2005).

[93] Watson, I. R., Irwin, M. S. & Ohh, M. NEDD8 pathways in cancer, sine quibus non. Cancer cell **19**, 168–176 (2011).

[94] Jiang, Z. et al. Menin upregulates FOXO1 protein stability by repressing Skp2-mediated degradation in β cells. Pancreas **48**, 267 (2019).

[95] Masui, K. et al. mtor complex 2 controls glycolytic metabolism in glioblastoma through FoxO acetylation and upregulation of c-myc. Cell metabolism **18**, 726–739 (2013).

[96] Lin, C.-W. et al. Daxx inhibits hypoxia-induced lung cancer cell metastasis by suppressing the hif-1α/hdac/slug axis. Nature Communications **7**, 1–16 (2016).

[97] Kao, S.-H. et al. Gsk3β controls epithelial–mesenchymal transition and tumor metastasis by chip-mediated degradation of slug. Oncogene **33**, 3172–3182 (2014).

[98] Gui, T., Sun, Y., Shimokado, A. & Muragaki, Y. The roles of mitogen-activated protein kinase pathways in tgf-β-induced epithelial-mesenchymal transition. Journal of signal transduction **2012** (2012).

[99] Choi, J., Park, S. Y. & Joo, C.-K. Transforming growth factor-β1 represses e-cadherin production via slug expression in lens epithelial cells. Investigative ophthalmology & visual science **48**, 2708–2718 (2007).

[100] Li, X. et al. Snail induction is an early response to gli1 that determines the efficiency of epithelial transformation. Oncogene **25**, 609–621 (2006).

[101] Li, X., Deng, W., Lobo-Ruppert, S. & Ruppert, J. Gli1 acts through snail and e-cadherin to promote nuclear signaling by β-catenin. Oncogene **26**, 4489–4498 (2007).

[102] Fendrich, V. et al. Snail and sonic hedgehog activation in neuroendocrine tumors of the ileum. Endocrine-Related Cancer **14**, 865–874 (2007).

[103] Yonemori, K. et al. Impact of snail and e-cadherin expression in pancreatic neuroendocrine tumors. Oncology letters **14**, 1697–1702 (2017).

[104] Marty-Santos, L. & Cleaver, O. Pdx1 regulates pancreas tubulogenesis and e-cadherin expression. Development **143**, 101–112 (2016).

[105] Ciccarelli, C. et al. Disruption of mek/erk/c-myc signaling radiosensitizes prostate cancer cells in vitro and in vivo. Journal of cancer research and clinical oncology **144**, 1685–1699 (2018).

[106] Murphy, L. O., MacKeigan, J. P. & Blenis, J. A network of immediate early gene products propagates subtle differences in mitogen-activated protein kinase signal amplitude and duration. Molecular and cellular biology **24**, 144–153 (2004).

[107] Gregory, M. A., Qi, Y. & Hann, S. R. Phosphorylation by glycogen synthase kinase-3 controls c-Myc proteolysis and subnuclear localization. The Journal of biological chemistry **278**, 51606–51612 (2003).

[108] Wu, X. et al. Rac1 activation controls nuclear localization of β-catenin during canonical wnt signaling. Cell **133**, 340–353 (2008).

[109] Cao, Y. et al. Nuclear-cytoplasmic shuttling of menin regulates nuclear translocation of β-catenin. Molecular and cellular biology **29**, 5477–5487 (2009).

[110] Klaus, A. & Birchmeier, W. Wnt signalling and its impact on development and cancer. Nature Reviews Cancer **8**, 387–398 (2008).

[111] Kotliarova, S. et al. Glycogen synthase kinase-3 inhibition induces glioma cell death through c-myc, nuclear factor-κb, and glucose regulation. Cancer Research **68**, 6643–6651 (2008).

[112] Wiegering, A. et al. Cip2a influences survival in colon cancer and is critical for maintaining myc expression. PloS one **8**, e75292 (2013).

[113] De, P., Carlson, J., Leyland-Jones, B. & Dey, N. Oncogenic nexus of cancerous inhibitor of protein phosphatase 2a (cip2a): an oncoprotein with many hands. Oncotarget **5**, 4581 (2014).

[114] MacDonald, B. T., Tamai, K. & He, X. Wnt/β-catenin signaling: components, mechanisms, and diseases. Developmental cell **17**, 9–26 (2009).

[115] Valenta, T., Hausmann, G. & Basler, K. The many faces and functions of β-catenin. The EMBO journal **31**, 2714–2736 (2012).

[116] Tian, X. et al. E-cadherin/β-catenin complex and the epithelial barrier. Journal of Biomedicine and Biotechnology **2011** (2011).

[117] Yan, J. et al. Menin interacts with iqgap1 to enhance intercellular adhesion of β-cells. Oncogene **28**, 973–982 (2009).

[118] Noritake, J., Watanabe, T., Sato, K., Wang, S. & Kaibuchi, K. Iqgap1: a key regulator of adhesion and migration. Journal of cell science **118**, 2085–2092 (2005).

[119] Kuroda, S. et al. Role of iqgap1, a target of the small gtpases cdc42 and rac1, in regulation of e-cadherin-mediated cell-cell adhesion. Science **281**, 832–835 (1998).

[120] Song, M. et al. Skp2 regulates the antiproliferative function of the tumor suppressor RASSF1A via ubiquitin-mediated degradation at the G 1–S transition. Oncogene **27**, 3176–3185 (2008).

[121] Ishida, E. et al. Attenuated expression of menin and p27 kip1 in an aggressive case of multiple endocrine neoplasia type 1 (men1) associated with an atypical prolactinoma and a malignant pancreatic endocrine tumor. Endocrine journal **58**, 287–296 (2011).

[122] Matkar, S., Thiel, A. & Hua, X. Menin: a scaffold protein that controls gene expression and cell signaling. Trends in biochemical sciences **38**, 394–402 (2013).

[123] Karnik, S. K. et al. Menin regulates pancreatic islet growth by promoting histone methylation and expression of genes encoding p27kip1 and p18ink4c. Proceedings of the National Academy of Sciences **102**, 14659–14664 (2005).

[124] Schnepp, R. W. et al. Mutation of tumor suppressor gene men1 acutely enhances proliferation of pancreatic islet cells. Cancer Research **66**, 5707–5715 (2006).

[125] Wu, T. & Hua, X. Menin represses tumorigenesis via repressing cell proliferation. American journal of cancer research **1**, 726 (2011).

[126] Taguchi, R. et al. Haploinsufficient and predominant expression of multiple endocrine neoplasia type 1 (men1)-related genes, mll, p27kip1 and p18ink4c in endocrine organs. Biochemical and biophysical research communications **415**, 378–383 (2011).

[127] Milne, T. A. et al. Menin and mll cooperatively regulate expression of cyclin-dependent kinase inhibitors. Proceedings of the National Academy of Sciences **102**, 749–754 (2005).

[128] Karger, S. et al. Foxo3a: a novel player in thyroid carcinogenesis? Endocrine-related cancer **16**, 189 (2009).

[129] Glauser, D. A. & Schlegel, W. The emerging role of foxo transcription factors in pancreatic β cells. Journal of Endocrinology **193**, 195–207 (2007).

[130] Kim, S. W., Kim, H. J., Chun, Y. J. & Kim, M. Y. Ceramide produces apoptosis through induction of p27kip1 by protein phosphatase 2a-dependent akt dephosphorylation in pc-3 prostate cancer cells. Journal of Toxicology and Environmental Health, Part A **73**, 1465–1476 (2010).

[131] Jiang, C., Wu, Y., Xia, Q. & Huang, Q. Novel molecular targets in malignant diseases of digestive system. Gastroenterology research and practice **2013** (2013).

[132] Zhong, L. et al. Essential role of skp2-mediated p27 degradation in growth and adaptive expansion of pancreatic β cells. The Journal of clinical investigation **117**, 2869–2876 (2007).

[133] Wong, C. et al. Two well-differentiated pancreatic neuroendocrine tumor mouse models. Cell Death & Differentiation **27**, 269–283 (2020).

[134] Zeng, P.-Y. & Berger, S. L. Lkb1 is recruited to the p21/waf1 promoter by p53 to mediate transcriptional activation. Cancer research **66**, 10701–10708 (2006).

[135] Tiainen, M., Vaahtomeri, K., Ylikorkala, A. & Mäkelä, T. P. Growth arrest by the lkb1 tumor suppressor: induction of p21waf1/cip1. Human molecular genetics **11**, 1497–1504 (2002).

[136] Bunz, F. et al. Requirement for p53 and p21 to sustain g2 arrest after dna damage. Science **282**, 1497–1501 (1998).

[137] Bazzi, W. et al. Men1 missense mutations impair sensitization to apoptosis induced by wild-type menin in endocrine pancreatic tumor cells. Gastroenterology **135**, 1698–1709 (2008).

[138] Wei, Z. et al. Stat3 interacts with skp2/p27/p21 pathway to regulate the motility and invasion of gastric cancer cells. Cellular signalling **25**, 931–938 (2013).

[139] Ohta, M. et al. p53-independent negative regulation of p21/cyclin-dependent kinase–interacting protein 1 by the sonic hedgehog-glioma-associated oncogene 1 pathway in gastric carcinoma cells. Cancer Research **65**, 10822–10829 (2005).

[140] Gansauge, S. et al. Overexpression of cyclin d1 in human pancreatic carcinoma is associated with poor prognosis. Cancer research **57**, 1634–1637 (1997).

[141] Filmus, J. et al. Induction of cyclin d1 overexpression by activated ras. Oncogene **9**, 3627–3633 (1994).

[142] Daksis, J. I., Lu, R. Y., Facchini, L. M., Marhin, W. W. & Penn, L. Myc induces cyclin d1 expression in the absence of de novo protein synthesis and links mitogen-stimulated signal transduction to the cell cycle. Oncogene **9**, 3635–3645 (1994).

[143] Hirai, H., Roussel, M. F., Kato, J., Ashmun, R. A. & Sherr, C. J. Novel ink4 proteins, p19 and p18, are specific inhibitors of the cyclin d-dependent kinases cdk4 and cdk6. Molecular and cellular biology **15**, 2672–2681 (1995).

[144] Diehl, J. A., Cheng, M., Roussel, M. F. & Sherr, C. J. Glycogen synthase kinase-3β regulates cyclin d1 proteolysis and subcellular localization. Genes & development **12**, 3499–3511 (1998).

[145] Shivakumar, L., Minna, J., Sakamaki, T., Pestell, R. & White, M. A. The RASSF1A tumor suppressor blocks cell cycle progression and inhibits Cyclin D1 accumulation. Molecular and Cellular Biology **22**, 4309–4318 (2002).

[146] Cicchillitti, L., Fasanaro, P., Biglioli, P., Capogrossi, M. C. & Martelli, F. Oxidative stress induces protein phosphatase 2A-dependent dephosphorylation of the pocket proteins prb, p107, and p130. Journal of Biological Chemistry **278**, 19509–19517 (2003).

[147] Tong, Y. et al. Pin1 inhibits PP2A-mediated rb dephosphorylation in regulation of cell cycle and S-phase DNA damage. Cell death & disease **6**, e1640–e1640 (2015).

[148] Kolupaeva, V. & Janssens, V. PP1 and PP2A phosphatases – cooperating partners in modulating retinoblastoma protein activation. The FEBS Journal **280**, 627–643 (2013).

[149] Alberts, A. S., Thorburn, A. M., Shenolikar, S., Mumby, M. C. & Feramisco, J. R. Regulation of cell cycle progression and nuclear affinity of the retinoblastoma protein by protein phosphatases. Proceedings of the National Academy of Sciences **90**, 388–392 (1993).

[150] Ludlow, J. W., Glendening, C. L., Livingston, D. M. & DeCarprio, J. A. Specific enzymatic dephosphorylation of the retinoblastoma protein. Molecular and Cellular Biology **13**, 367–372 (1993).

[151] Hiebert, S., Chellappan, S., Horowitz, J. & Nevins, J. The interaction of rb with e2f coincides with an inhibition of the transcriptional activity of e2f. Genes & development **6**, 177–185 (1992).

[152] Brehm, A. et al. Retinoblastoma protein recruits histone deacetylase to repress transcription. Nature **391**, 597–601 (1998).

[153] Ohtani, K., Degregori, J. & Nevins, J. R. Regulation of the cyclin e gene by transcription factor e2f1. Proceedings of the National Academy of Sciences **92**, 12146–12150 (1995).

[154] Connell-Crowley, L., Harper, J. W. & Goodrich, D. W. Cyclin d1/cdk4 regulates retinoblastoma protein-mediated cell cycle arrest by site-specific phosphorylation. Molecular biology of the cell **8**, 287–301 (1997).

[155] Kim, Y.-C. et al. Rb regulates pancreas development by stabilizing pdx1. The EMBO journal **30**, 1563–1576 (2011).

[156] Dimri, G. P., Nakanishi, M., Desprez, P.-Y., Smith, J. R. & Campisi, J. Inhibition of e2f activity by the cyclin-dependent protein kinase inhibitor p21 in cells expressing or lacking a functional retinoblastoma protein. Molecular and cellular biology **16**, 2987–2997 (1996).

[157] Ray, A., James, M. K., Larochelle, S., Fisher, R. P. & Blain, S. W. p27kip1 inhibits cyclin d-cyclin-dependent kinase 4 by two independent modes. Molecular and cellular biology **29**, 986–999 (2009).

[158] Toyoshima, H. & Hunter, T. p27, a novel inhibitor of g1 cyclin-cdk protein kinase activity, is related to p21. Cell **78**, 67–74 (1994).

[159] Jackson, P. K., Chevalier, S., Philippe, M. & Kirschner, M. W. Early events in dna replication require cyclin e and are blocked by p21cip1. The Journal of cell biology **130**, 755–769 (1995).

[160] Feng, Z. et al. Menin and daxx interact to suppress neuroendocrine tumors through epigenetic control of the membrane metallo-endopeptidase. Cancer research **77**, 401–411 (2017).

[161] Li, J. et al. Berberine represses daxx gene transcription and induces cancer cell apoptosis. Laboratory Investigation **93**, 354–364 (2013).

[162] Yamashita, K., Discher, D. J., Hu, J., Bishopric, N. H. & Webster, K. A. Molecular regulation of the endothelin-1 gene by hypoxia. Journal of Biological Chemistry **276**, 12645–12653 (2001).

[163] Sun, P., Xiong, H., Kim, T. H., Ren, B. & Zhang, Z. Positive inter-regulation between β-catenin/t cell factor-4 signaling and endothelin-1 signaling potentiates proliferation and survival of prostate cancer cells. Molecular Pharmacology **69**, 520–531 (2006).

[164] Kim, T. H., Xiong, H., Zhang, Z. & Ren, B. β-catenin activates the growth factor endothelin-1 in colon cancer cells. Oncogene **24**, 597–604 (2005).

[165] Gao, D. et al. Phosphorylation by Akt1 promotes cytoplasmic localization of Skp2 and impairs APCCdh1-mediated Skp2 destruction. Nature Cell Biology **11**, 397–408 (2009).

[166] Schlienger, S., Ramirez, R. A. M. & Claing, A. ARF1 regulates adhesion of MDA-MB-231 invasive breast cancer cells through formation of focal adhesions. Cellular Signalling **27**, 403–415 (2015).

[167] Vacca, F., Bagnato, A., Catt, K. J. & Tecce, R. Transactivation of the epidermal growth factor receptor in endothelin-1-induced mitogenic signaling in human ovarian carcinoma cells. Cancer Research **60**, 5310–5317 (2000).

[168] Reiske, H. R., Zhao, J., Han, D. C., Cooper, L. A. & Guan, J.-L. Analysis of FAK-associated signaling pathways in the regulation of cell cycle progression. Febs Letters **486**, 275–280 (2000).

[169] Lim, Y. et al. Focal adhesion kinase is negatively regulated by phosphorylation at tyrosine 407. Journal of Biological Chemistry **282**, 10398–10404 (2007).

[170] Ning, L., Chen, H. & Kunnimalaiyaan, M. Focal adhesion kinase, a downstream mediator of raf-1 signaling, suppresses cellular adhesion, migration, and neuroendocrine markers in bon carcinoid cells. Molecular Cancer Research **8**, 775–782 (2010).

[171] De, P., Rozeboom, B. J., Aske, J. C. & Dey, N. Active rac1 promotes tumorigenic phenotypes and therapy resistance in solid tumors. Cancers **12** (2020).

[172] Yang, H. W. et al. Cooperative activation of pi3k by ras and rho family small gtpases. Molecular Cell **47**, 281–290 (2012).

[173] Chang, F., Lemmon, C. A., Park, D. & Romer, L. H. FAK potentiates Rac1 activation and localization to matrix adhesion sites: A role for βPIX. Molecular Biology of the Cell **18**, 253–264 (2007).

[174] Kallergi, G., Agelaki, S., Markomanolaki, H., Georgoulias, V. & Stournaras, C. Activation of fak/pi3k/rac1 signaling controls actin reorganization and inhibits cell motility in human cancer cells. Cellular Physiology and Biochemistry **20**, 977–986 (2007).

[175] Gulhati, P. et al. mtorc1 and mtorc2 regulate emt, motility, and metastasis of colorectal cancer via rhoa and rac1 signaling pathways. Cancer research **71**, 3246–3256 (2011).

[176] Liu, L. et al. Rapamycin inhibits cytoskeleton reorganization and cell motility by suppressing rhoa expression and activity. Journal of Biological Chemistry **285**, 38362–38373 (2010).

[177] Sun, T., Rodriguez, M. & Kim, L. Glycogen synthase kinase 3 in the world of cell migration. Development, Growth & Differentiation **51**, 735–742 (2009).

[178] Khoo, S. et al. Regulation of insulin gene transcription by erk1 and erk2 in pancreatic β cells. Journal of Biological Chemistry **278**, 32969–32977 (2003).

[179] Boucher, M.-J., Selander, L., Carlsson, L. & Edlund, H. Phosphorylation marks ipf1/pdx1 protein for degradation by glycogen synthase kinase 3-dependent mechanisms. Journal of Biological Chemistry **281**, 6395–6403 (2006).

[180] Humphrey, R. K., Yu, S.-M., Flores, L. E. & Jhala, U. S. Glucose regulates steady-state levels of pdx1 via the reciprocal actions of gsk3 and akt kinases. Journal of Biological Chemistry **285**, 3406–3416 (2010).

[181] Jiang, Z. et al. Human proislet peptide promotes pancreatic progenitor cells to ameliorate diabetes through foxo1/menin-mediated epigenetic regulation. Diabetes **67**, 1345–1355 (2018).

[182] Ogihara, T., Vanderford, N. L., Maier, B., Stein, R. W. & Mirmira, R. G. Expression and function of set7/9 in pancreatic islets. Islets **1**, 269–272 (2009).

[183] Wu, Y. et al. Dual oxidase 2 and pancreatic adenocarcinoma: Ifn-γ-mediated dual oxidase 2 overexpression results in h2o2-induced, erk-associated up-regulation of hif-1α and vegf-a. Oncotarget **7**, 68412 (2016).

[184] Kato, S. et al. Muc5ac mucin gene regulation in pancreatic cancer cells. International journal of oncology **29**, 33–40 (2006).

[185] Ehlers, R. A., Zhang, Y., Hellmich, M. R. & Evers, B. M. Neurotensin-mediated activation of mapk pathways and ap-1 binding in the human pancreatic cancer cell line, mia paca-2. Biochemical and biophysical research communications **269**, 704–708 (2000).

[186] Mann, B. et al. Target genes of β-catenin–t cell-factor/lymphoid-enhancer-factor signaling in human colorectal carcinomas. Proceedings of the National Academy of Sciences **96**, 1603–1608 (1999).

[187] Wang, Q., Sun, Z. & Yang, H. Downregulation of tumor suppressor pdcd4 promotes invasion and activates both β-catenin/tcf and ap-1-dependent transcription in colon carcinoma cells. Oncogene **27**, 1527–1535 (2008).

[188] Zhang, W., Hart, J., McLeod, H. L. & Wang, H. L. Differential expression of the ap-1 transcription factor family members in human colorectal epithelial and neuroendocrine neoplasms. American journal of clinical pathology **124**, 11–19 (2005).

[189] Karin, M., Liu, Z.-g. & Zandi, E. Ap-1 function and regulation. Current opinion in cell biology **9**, 240–246 (1997).

[190] Cavigelli, M. et al. The tumor promoter arsenite stimulates ap-1 activity by inhibiting a jnk phosphatase. The EMBO journal **15**, 6269–6279 (1996).

[191] Czaja, M. J. The future of gi and liver research: editorial perspectives. iii. jnk/ap-1 regulation of hepatocyte death. American Journal of Physiology **284**, G875–G879 (2003).

[192] Chan, C. S. et al. ATRX, DAXX or MEN1 mutant pancreatic neuroendocrine tumors are a distinct alpha-cell signature subgroup. Nature Communications **9**, 1–10 (2018).

[193] Wasylishen, A. R. et al. Daxx maintains endogenous retroviral silencing and restricts cellular plasticity in vivo. Science Advances **6**, eaba8415 (2020).

[194] Sun, C. et al. Context matters–Daxx and Atrx are not robust tumor suppressors in the murine endocrine pancreas. Disease models & Mechanisms **15**, dmm049552 (2022).

[195] Wang, F. et al. Prognostic significance of altered ATRX/DAXX gene in pancreatic neuroendocrine tumors: a meta-analysis. Frontiers in Endocrinology **12**, 691557 (2021).

[196] Jiao, Y. et al. DAXX/ATRX, MEN1, and mTOR pathway genes are frequently altered in pancreatic neuroendocrine tumors. Science **331**, 1199–1203 (2011).

[197] Park, J. K. et al. DAXX/ATRX and MEN1 genes are strong prognostic markers in pancreatic neuroendocrine tumors. Oncotarget **8**, 49796 (2017).

[198] Kim, J. Y. et al. Alternative lengthening of telomeres in primary pancreatic neuroendocrine tumors is associated with aggressive clinical behavior and poor survival in pancreatic neuroendocrine neoplasms. Clinical Cancer Research **23**, 1598–1606 (2017).

[199] Raj, N. et al. Real-time genomic characterization of metastatic pancreatic neuroendocrine tumors has prognostic implications and identifies potential germline actionability. JCO precision oncology **2**, 1–18 (2018).

[200] Marinoni, I. et al. Loss of daxx and atrx are associated with chromosome instability and reduced survival of patients with pancreatic neuroendocrine tumors. Gastroenterology **146**, 453–460 (2014).

[201] Hackeng, W. M. et al. Non-functional pancreatic neuroendocrine tumours: ATRX/DAXX and alternative lengthening of telomeres (ALT) are prognostically independent from ARX/PDX1 expression and tumour size. Gut **71**, 961–973 (2022).

[202] Singhi, A. D. et al. Alternative lengthening of telomeres and loss of DAXX/ATRX expression predicts metastatic disease and poor survival in patients with pancreatic neuroendocrine tumorsassessment of ALT and DAXX/ATRX in PanNETs. Clinical Cancer Research **23**, 600–609 (2017).

[203] Larson, A. et al. Pancreatic neuroendocrine tumors in patients with tuberous sclerosis complex. Clinical Genetics **82**, 558–563 (2012).

[204] Zhang, J. et al. Current understanding of the molecular biology of pancreatic neuroendocrine tumors. Journal of the National Cancer Institute **105**, 1005–1017 (2013).

[205] Schmitt, A. M. et al. VHL inactivation is an important pathway for the development of malignant sporadic pancreatic endocrine tumors. Endocrine-Related Cancer **16**, 1219 – 1227 (2009).

[206] Sennino, B. et al. Suppression of Tumor Invasion and Metastasis by Concurrent Inhibition of c-Met and VEGF Signaling in Pancreatic Neuroendocrine Tumors. Cancer Discovery **2**, 270–287 (2012).

[207] Münzberg, C. et al. IGF-1 drives chromogranin A secretion via activation of Arf1 in human neuroendocrine tumour cells. Journal of Cellular and Molecular Medicine **19**, 948–959 (2015).

[208] François, R. A. et al. Targeting Focal Adhesion Kinase and Resistance to mTOR Inhibition in Pancreatic Neuroendocrine Tumors. JNCI: Journal of the National Cancer Institute **107** (2015).

[209] Sippel, R. S., Carpenter, J. E., Kunnimalaiyaan, M., Lagerholm, S. & Chen, H. Raf-1 activation suppresses neuroendocrine marker and hormone levels in human gastrointestinal carcinoid cells. American Journal of Physiology-Gastrointestinal and Liver Physiology **285**, G245–G254 (2003).

[210] Kunnimalaiyaan, M., Ndiaye, M. & Chen, H. Neuroendocrine tumor cell growth inhibition by ZM336372 through alterations in multiple signaling pathways. Surgery **142**, 959–964 (2007).

[211] Guo, S. et al. Frequent overexpression of cyclin D1 in sporadic pancreatic endocrine tumours. Journal of Endocrinology **179**, 73 – 79 (2003).

[212] Zhou, H. et al. Integrated clinicopathological features and gene microarray analysis of pancreatic neuroendocrine tumors. Gene **625**, 72–77 (2017).

[213] Motylewska, E., Braun, M. & Stępień, H. High Expression of NEK2 and PIM1, but Not PIM3, Is Linked to an Aggressive Phenotype of Bronchopulmonary Neuroendocrine Neoplasms. Endocrine Pathology **31**, 264–273 (2020).

[214] Sadanandam, A. et al. A cross-species analysis in pancreatic neuroendocrine tumors reveals molecular subtypes with distinctive clinical, metastatic, developmental, and metabolic characteristics. Cancer Discovery **5**, 1296–1313 (2015).

[215] Gilbert, J. A. et al. Molecular markers for novel therapeutic strategies in pancreatic endocrine tumors. Pancreas **42**, 411–421 (2013).

[216] Maharjan, C. K. et al. Pancreatic neuroendocrine tumors: Molecular mechanisms and therapeutic targets. Cancers **13**, 5117 (2021).

[217] Soler, A. et al. Therapeutic Benefit of Selective Inhibition of p110α PI3-Kinase in Pancreatic Neuroendocrine Tumors. Clinical Cancer Research **22**, 5805–5817 (2016).

[218] Ghayouri, M., Boulware, D., Nasir, A., Strosberg, J. & Kvols, L. Activation of the serine/theronine protein kinase Akt in enteropancreatic neuroendocrine tumors. Anticancer Research **30**, 5063–5067 (2010).

[219] Thomas-Marques, L. et al. Prospective endoscopic ultrasonographic evaluation of the frequency of non-functioning pancreaticoduodenal endocrine tumors in patients with multiple endocrine neoplasia type 1. American Journal of Gastroenerology **101**, 266–273 (2006).

[220] Missiaglia, E. et al. Pancreatic Endocrine Tumors: Expression Profiling Evidences a Role for AKT-mTOR Pathway. Journal of Clinical Oncology **28**, 245–255 (2010).

[221] Chang, T.-M. et al. PTEN regulates invasiveness in pancreatic neuroendocrine tumors through DUSP19-mediated VEGFR3 dephosphorylation. Journal of Biomedical Science **29** (2022).

[222] Prada, E. T. A. et al. The role of GSK3 and its reversal with GSK3 antagonism in everolimus resistance. Endocrine-Related Cancer **25**, 893 – 908 (2018).

[223] Elmadbouh, O. H. M., Pandol, S. J. & Edderkaoui, M. Glycogen Synthase Kinase 3β: A True Foe in Pancreatic Cancer. International Journal of Molecular Sciences **23** (2022).

[224] Cavalcanti, E. et al. Altered miRNAs Expression Correlates With Gastroenteropancreatic Neuroendocrine Tumors Grades. Frontiers in Oncology **10** (2020).

[225] Glauser, D. A. & Schlegel, W. The emerging role of FOXO transcription factors in pancreatic β cells. Journal of Endocrinology **193**, 195 – 207 (2007).

[226] Malpeli, G. et al. Methlyation-associated down-regulation of RASSF1A and up-regulation of RASSF1C in pacnreatic endocrine tumors. BMC Cancer **11**, 351 (2011).

[227] Bocchini, M. et al. Biomarkers for Pancreatic Neuroendocrine Neoplasms (PanNENs) Management - An Update Review. Frontiers in Oncology **10**, 831 (2020).

[228] Hu, W. et al. Gene amplification in well-differentiated pancreatic neuroendocrine tumors inactivate the p53 pathway. Genes & Cancer **1**, 360–368 (2010).

[229] van Riet, J. et al. The genomic landscape of 85 advanced neuroendocrine neoplasms reveals subtype-heterogeneity and potential therapeutic targets. Nature Communications **12**, 4612 (2021).

[230] Chan, J. & Kulke, M. Targeting the mTOR Signaling Pathway in Neuroendocrine Tumors. Current Treatment Options in Oncology **15**, 365–379 (2014).

[231] Wang, D. G., Johnston, C. F. & Buchanan, K. D. Oncogene expression in gastroenteropancreatic neuroendocrine tumors: implications for pathogenesis. Cancer **80**, 668–675 (1997).

[232] Stålberg, P. et al. Transfection of the Multiple Endocrine Neoplasia Type 1 Gene to a Human Endocrine Pancreatic Tumor Cell Line Inhibits Cell Growth and Affects Expression of JunD, δ-Like Protein 1/Preadipocyte Factor-1, Proliferating Cell Nuclear Antigen, and QM/Jif-1. The Journal of Clinical Endocrinology & Metabolism **89**, 2326–2337 (2004).

[233] Vandamme, T. et al. Whole-exome characterization of pancreatic neuroendocrine tumor cell lines BON-1 and QGP-1. Journal of Molecular Endocrinology **54**, 137–147 (2015).

[234] Conemans, E. B. et al. Expression of p27 and p18 in human multiple endocrine neoplasia type 1-related pancreatic neuroendocrine tumors. Journal of Endocrinological Investigations **41**, 655–661 (2018).

[235] Kim, C. et al. Reduced RNA-binding protein HuD in pancreatic neuroendocrine tumors lowers p27^{Kip1} levels linked to poor prognosis. The Journal of Pathology **246**, 231–243 (2018).

[236] Liu, S. et al. Prognostic impact of p16 and p21 on gastorenteropancreatic neuroendocrine tumors. Oncology Letters **6**, 1641–1645 (2013).

[237] Chung, D. C. et al. Overexpression of Cyclin D1 Occurs Frequently in Human Pancreatic Endocrine Tumors1. The Journal of Clinical Endocrinology & Metabolism **85**, 4373–4378 (2000).

[238] Yamauchi, Y. et al. Rb and p53 Execute Distinct Roles in the Development of Pancreatic Neuroendocrine Tumors. Cancer Research **80**, 3620–3630 (2020).

[239] Cives, M. et al. DAXX mutations as potential genomic markers of malignant evolution in small nonfunctioning pancreatic neuroendocrine tumors. Scientific Reports 18614 (2019).

[240] Cingarlini, S. et al. Profiling mTOR Pathway in Neuroendocrine Tumors. In Raymond, E., Faivre, S. & Ruszniewski, P. (eds.) Management of Neuroendocrine Tumors of the Pancreas and Digestive Tract, 9–27 (Springer, Paris, 2014).

[241] Lopez-Aguiar, A. G. et al. STAT3 Inhibition for GAstorenteropancreatic Neuroendocrine Tumors: Potential for a New Therapeutic Target? Journal of Gastrointestinal Surgery **24**, 1138–1148 (2020).

[242] Jiang, X. et al. Targeting beta-catenin signaling for therapeutic intervention in MEN1-deficient pancreatic neuroendocrine tumours. Nature Communications **5** (2014).

[243] Pozas, J. et al. Targeting angiogenesis in pancreatic neuroendocrine tumors: Resistance mechanisms. International Journal of Molecular Sciences **20** (2019).

[244] Hackeng, W. M. et al. Non-functional pancreatic neuroendocrine tumours: Atrx/daxx and alternative lengthening of telomeres (alt) are prognostically independent from arx/pdx1 expression and tumour size. Gut **71**, 961–973 (2022).

[245] Roy, N. et al. PDX1 dynamically regulates pancreatic ductal adenocarcinoma initiation and maintenance. Genes & Development **30**, 2669–2683 (2016).
